# Supplementary material for: Effect of intermittent preventive treatment for malaria with dihydroartemisinin-piperaquine on immune responses to vaccines among rural Ugandan adolescents: randomised controlled trial protocol B for the ‘POPulation differences in VACcine responses’ (POPVAC) programme
Source: BMJ Open. 2021 Feb 16;11(2):e040427. doi: 10.1136/bmjopen-2020-040427 (PMC7893608; doi:10.1136/bmjopen-2020-040427)
Supplement: Supplementary data [file bmjopen-2020-040427supp001.pdf]

SUPPLEMENTARY INFORMATION

The effect of intermittent preventive treatment for malaria with dihydroartemisinin-piperaquine on immune responses to vaccines among rural Ugandan adolescents: randomised controlled trial protocol B for the 'POPulation differences in VACcine responses' (POPVAC) programme

Agnes Natukunda[1,¶], Ludoviko Zirimenya[1,¶], Gyaviira Nkurunungi[1,¶,*], Jacent Nassuuna[1], Gloria Oduru[1], Rebecca Amongin[1], Prossy N Kabuubi[1], Alex Mutebe[1], Caroline Onen[1], Susan Amongi[1], Esther Nakazibwe[1], Florence Akello[1], Samuel Kiwanuka[1], Fred Kiwudhu[1], Moses Sewankambo[1], Denis Nsubuga[1], Robert Kizindo[1], Sarah G Staedke[2,3], Stephen Cose[1,3], Emily L Webb[4], Alison M Elliott[1,3] **for the POPVAC trial team**

[1]Immunomodulation and Vaccines Programme, Medical Research Council/Uganda Virus Research Institute and London School of Hygiene and Tropical Medicine (MRC/UVRI and LSHTM) Uganda Research Unit, Entebbe, Uganda

[2]Infectious Diseases Research Collaboration, Kampala, Uganda

[3]Department of Clinical Research, London School of Hygiene and Tropical Medicine, London, United Kingdom

[4]MRC Tropical Epidemiology Group, Department of Infectious Disease Epidemiology, London School of Hygiene and Tropical Medicine, London, United Kingdom

[¶]These authors contributed equally

***Correspondence:** Gyaviira Nkurunungi; Gyaviira.Nkurunungi@mrcuganda.org

Natukunda A, *et al. BMJ Open* 2021; 11:e040427. doi: 10.1136/bmjopen-2020-040427

21 SUPPLEMENTARY INFORMATION

Table S1. POPVAC B: schedule of visits and procedures

VISIT NUMBER	1	2, 3	4	5	6[9]	6.2	7	8	9, 10, 11, 12, 13	14	15	16	17,18, 19, 20, 21	22
**WEEKS FROM 1ST IMMUNISATION**	**-8[1]**	**-6[10], -2**	**0**	**2**	**4**	**4 + 4 days**	**6**	**8**	**10, 14, 18, 22 , 26**	**28**	**30**	**32**	**34, 38, 42, 46, 50**	**52**
	**Screening**	**Treatment (Rx only)**	**Immunisation**	**Treatment (Rx only)**	**Immunisations**		**Rx only**	**Primary endpoint (PE)**	**Rx only**	**Immunisations**	**Rx only**	**PE[2]**	**Rx only**	**Secondary endpoint (SE)**
**RANDOMISED DIHYDROARTEMISININ-PIPERAQUINE INTERVENTION**														
DP treatment arm (x)		x		x			x		x		x		x	
DP control arm (o represents placebo)		o		o			o		o		o		o	
Albendazole								x[3]				x[3]		x[3]
Praziquantel								x[3,6]						x[3,6]
**VACCINES**														
BCG			x											
YF-17D					x									
Ty21a					x									
HPV					x			[x][4]		x				
Td										x				[x][5]
**INVESTIGATIONS/PROCEDURES**														
Inclusion/exclusion criteria	x													
Informed consent	x													
Questionnaire	x		x		x	x		x		x		x		x
Examination	x		(x)		(x)	(x)		(x)		(x)		(x)		(x)
Urine β-HCG test (female only) 1mL	x		x		x					x				
Urine YF viral load						x								
Stool for PCR and storage	x									x				x
Stool for coproantibody and storage	x							x						
**BLOOD SAMPLES**														
Malaria PCR (1ml)	x		x		x			x		x		x		x
Serology for HIV, prior malaria and *S. mansoni* (0.5ml)	x													
Mansonella perstans (1ml)	x													
Serum/plasma CAA (1ml)	x									x				x
Hb[8]/Full blood count (0.5ml)	x		x		x					x				
Assessments of pre-immunisation responses, and/or vaccine response outcomes and/or exploratory immunology; storage[7] (10-20ml)			x		x			x		x		x		x
Blood for gene expression (2ml)			x		x					x				
Blood vol (mL)	4		27		17			14-24		27		14		14

Cumulative blood vol (mL)[7]	4		31		48			72		99		113		127

PE: primary endpoint; **SE:** secondary endpoint; **Rx only**: treatment only
Immunisation days highlighted in green, primary end point days in red, days for treatment only in grey
(**x**) performed if clinically indicated

1. Screening and enrolment into Project B will take place at least 8 weeks before immunisation 0 to allow initiation of the DP intervention.
2. Week 32 is primary endpoint for responses to Td given at 28 weeks; secondary endpoint for HPV booster
3. Treatments given after sampling when schedules coincide
4. Week 8 HPV dose will be given for previously-unvaccinated girls aged ≥14 years
5. Week 52 Td booster dose will be provided as a service
6. Praziquantel if required in accordance with Ministry of Health policy for the selected schools
7. Exploratory immunology blood volume will be guided by guidelines from Harvard Mass General, where a maximum of 3ml/kg body weight is taken at any one time point and not more than 3ml/kg is taken over any 8 week period (ref http://www.drgreene.com/21_1616.html.) These guidelines have been followed in a previous study vaccinating adolescents with investigational tuberculosis vaccine MVA85A (in Uganda).[1] The total blood volume planned is 72 ml over the initial intensive sampling period of 8 weeks. Revision of sample volumes based on weight will only be required for participants who weigh less than 24 kg; the average weight of children aged 9 years is expected to be 28kg (with 21kg the 3rd centile) with greater weights for older children.[2]
8. At Baseline, it will only be Hb estimation by Haemocue
9. Oral typhoid vaccine doses will be administered on four alternate days namely visit 6, 6.1, 6.2 and 6.3.
10. The first DP and Placebo administration at week -6 will be administered at the end of the screening visit

Further information on the recruitment criteria

- Participants who are excluded from the trial because they have been discovered during screening procedures to be suffering from a previously undiagnosed condition thought to require further medical attention will be referred appropriately for further investigation and treatment.
- Participants discovered to have severe anaemia will be excluded from the trial and treated for anaemia and any evident underlying cause.
- Participants discovered to be HIV-positive will be counselled and offered a CD4 T-cell count and referred to a provider for further care and antiretroviral treatment (since this is indicated for all HIV-positive children under national guidelines).
- Participants discovered to be pregnant will be counselled and referred to an antenatal clinic of their choice.

This trial proposes to recruit all participants within a short time-frame. It will not, therefore, be possible to reconsider enrolment of potential participants with temporary exclusion criteria after treatment and resolution of the condition.

Further rationale for the selection of vaccines

Bacillus Calmette-Guérin (BCG)

BCG is a live, replicating parenteral vaccine, the only licensed vaccine against TB. BCG vaccine for these studies will be obtained from the Serum Institute of India either directly, or through a supplier in Uganda. The Serum Institute of India provides much of the BCG vaccine used in Uganda.

Worldwide, TB is among the top 10 causes of death; Uganda has an estimated incidence of 202/100,000 people.[3] Infectious, sputum positive, pulmonary TB classically emerges in adolescence, driving the on-going epidemic.[4] Thus adolescent booster immunisation is a key TB control strategy.[5] However, as discussed above, BCG vaccine response and efficacy are often impaired in tropical and rural settings[6-8] and new TB vaccines are similarly affected.[9] In the past, WHO has been hesitant to recommend BCG re-vaccination. However, in 2017 WHO's Strategic Advisory Group of Experts (SAGE) recommended, "Further research is warranted to explore whether certain sub-groups of age, geographic or *M. tuberculosis* exposure categories would benefit from re-vaccination."[10] Recent results suggest that, despite the variability of BCG efficacy between populations, BCG vaccination in adolescence offers benefit in some tropical settings, especially for individuals who are not yet infected with *Mycobacterium tuberculosis*, and may also be cost-effective.[7 11] Also, BCG vaccine is currently being used among adolescents in South Africa as a comparator in a trial of a novel TB vaccine (trial registration NCT02075203). To our knowledge, BCG efficacy in Ugandan adolescents, and differences in BCG vaccine responses between urban and rural Ugandan populations, have not been tested. Information obtained from this study is expected to further inform the use of BCG in adolescents, and also to inform the development of new vaccines for tuberculosis.

Yellow fever vaccine

Yellow fever vaccine YF-17D is a live replicating parenteral vaccine. The vaccine (Stamaril; Sanofi Pasteur) is available for purchase in Uganda. Yellow Fever (YF) causes outbreaks in Uganda and the wider region[12] and YF-17D is a candidate for Uganda's expanded programme on immunisation. Lower vaccine replication, lower neutralising antibody induction, and greater waning, are described in Uganda compared to Switzerland.[13] YF-17D is a potential vector for novel vaccine constructs,[14] adding relevance to vaccine development.

Typhoid vaccine Ty21a

Typhoid vaccine Ty21a is a live replicating oral vaccine and also a potential vector for new vaccine constructs.[15] Ty21a vaccine will be purchased from PaxVax, Redwood City, California. Substantial, multi-year typhoid outbreaks occur in Uganda and immunisation campaigns have been advocated as cost effective.[16] Schistosomiasis has been associated with prolonged S. typhi infection[17] and impaired antibody responses to killed typhoid vaccines.[18]

Ty21a was developed in the 1970s. Although not routinely used in Uganda, it has been (and is currently) registered in many countries. It was first registered in the United States and United Kingdom in the 1980s, and is recommended by the World Health Organisation for both endemic and epidemic settings.[19] It has comparable efficacy to the parenteral Vi polysaccharide typhoid vaccine, good durability and minimal adverse effects.[19] It is proposed for use in this study to model effects of study exposures and intervention on the response to a live oral vaccine.

The Ty21a vaccine is given as a three-dose regimen on alternate days.

Human Papilloma Virus (HPV) vaccine

Human Papilloma Virus (HPV) vaccine is a protein virus-like particle. The quadrivalent HPV Vaccine Gardasil (Merck) is available for purchase in Uganda and is the vaccine used by the national EPI programme. Studies after three vaccine doses have found somewhat enhanced responses in the presence of malaria, but no effect of helminths.[20] No study has previously investigated parasite effects on the priming response, but recent results for tetanus suggest that priming may be more susceptible than boosting to adverse effects.[21] This will be important if forthcoming trials support single-dose HPV immunisation (NCT02834637). HPV immunisation is being rolled out among girls to prevent cervical neoplasia, the commonest cancer among Ugandan women and we will coordinate provision with the national HPV immunisation programme.[22] HPV immunisation is also beneficial for boys since HPV infection is associated with anogenital warts, anal cancer and oropharyngeal cancers in both males and females, and with penile cancer in men,[23] and we will include boys in these studies.

BMJ Publishing Group Limited (BMJ) disclaims all liability and responsibility arising from any reliance placed on this supplemental material which has been supplied by the author(s)

Tetanus and diphtheria vaccines

Tetanus and diphtheria vaccines comprise inert toxoids (Td). Booster immunisation is recommended for young women to prevent maternal and neonatal tetanus. Recent evidence emphasises the need to protect young men also.[24] Uganda's EPI programme recommends tetanus boosters in adolescence and plans to change from tetanus alone to Td in 2018.

Additional considerations regarding the vaccine schedule

Live vaccines given in combination may influence the response to each other – a phenomenon described as "interference". Observations in the 1960s suggested that elevated circulating interferon (IFN)-γ after measles immunisation might interfere with the response to Vaccinia[25] and, to avoid such interference between live vaccines, it was recommended that live vaccines be given either together or three to four weeks apart.[26] However, with the introduction of new, live vaccines into use, Public Health England reviewed and revised this recommendation in 2014, limiting it to vaccines for which there was an evidence base (**Table S2**). We have adopted a four-week interval between BCG immunisation and the other proposed live vaccines (YF and Ty21a), which will be given together.[27]

Non-live vaccines can be given at the same time as live vaccines and there are no specific recommendations as to the number of non-live vaccines that can be given together. Two injections can be given into the same muscle although it is suggested that these should be at least 2.5 cm apart in case it is necessary to distinguish local adverse reactions to the two injections.[28] Our schedule avoids giving more than two injections on the same date but at week 4 it may be appropriate to give two into the deltoid muscle of the same arm if a BCG scar is developing on the other arm. Nevertheless, uncertainties remain regarding the effects of vaccines on responses to each other. Generalisations from this programme of work will need to take potential "interference" between vaccines into account.

Table S2: Public Health England recommendations for giving more than one live attenuated vaccine in current use in the UK[29]

Vaccine combinations	Recommendations
Yellow Fever and Measles, Mumps, Rubella (MMR)	A four week minimum interval period should be observed between the administration of these two vaccines. Yellow Fever and MMR should not be administered on the same day.
Varicella (and zoster) vaccine and MMR	If these vaccines are not administered on the same day, then a four week minimum interval should be observed between vaccines.
Tuberculin skin testing (Mantoux) and MMR	If a tuberculin skin test has already been initiated, then MMR should be delayed until the skin test has been read unless protection against measles is required urgently. If a child has had a recent MMR, and requires a tuberculin test, then a four week interval should be observed.
All currently used live vaccines (BCG, rotavirus, live attenuated influenza vaccine (LAIV), oral typhoid vaccine, yellow fever, varicella, zoster and MMR) and tuberculin (Mantoux) skin testing.	Apart from those combinations listed above, these live vaccines can be administered at any time before or after each other. This includes tuberculin (Mantoux) skin testing.

Immunisation Postponement Criteria

If any one of the following is identified at the time scheduled for immunisation, the participant may be immunised at a later date, or withdrawn, at the discretion of the Investigator. The participant must be followed until resolution of the event as with any adverse event:

- Acute disease at the time of immunisation. Acute disease is defined as the presence of a moderate or severe illness with or without fever. All vaccines can be administered to persons with a minor illness such as diarrhoea or mild upper respiratory infection with or without low-grade fever, i.e. temperature of ≤37.5°C (99.5°F)
- Temperature of >37.5°C (99.5°F) at the time of immunisation
- Taking antibiotics or antimalarials currently, or within the past 7 days, of the date of Ty21a administration (ascertained verbally)

Vaccine storage and transport

In order to maintain a reliable vaccine cold chain, the vaccines and diluents to be used will be stored and transported within the recommended temperature range of +2°C to +8°C. Care will be taken to ensure that the vaccines are not frozen. BCG, being sensitive to light, will be kept in the dark (normally within its secondary packaging) for as long as possible to protect it during storage and transportation. All vaccines will be kept in appropriate refrigeration equipment with a temperature monitoring device to ensure temperatures remain between +2°C and +8°C. Cold boxes/vaccines carriers with temperature monitors will be used to transport vaccines and the diluents from the MRC/UVRI and LSHTM Uganda Research Unit (Entebbe) to Jinja and while transporting vaccines to immunisation sessions. Designated staff will be given responsibility for managing the vaccine cold chain. All cold chain equipment including the temperature monitoring devices used for this project will comply with relevant technical specifications as defined by the EPI standards. Basic routine maintenance will be regularly carried out on all cold chain equipment.

Additional laboratory measurements

Additional assays will comprise HIV serology, pregnancy testing and full blood counts. HIV testing and pregnancy testing will be accompanied by appropriate counselling by trained staff.

HIV serology will be done on blood samples using rapid tests and according to prevailing national algorithms.[30] This will be done at baseline.

- Pregnancy testing will be done using urine samples and standard operating procedures for assessment of urine β-human chorionic gonadotropin (βhCG). This will be done at baseline and before immunisation on each immunisation day.
- Full blood counts will be conducted using a haematology analyser. Mild, moderate and severe anaemia will be defined according to WHO guidelines, by age.[31] This will be done at baseline to test

for anaemia as part of the eligibility assessment, and pre-immunisation as part of the assessment of immunological profile.

Individuals found to be HIV positive or pregnant will be referred to appropriate providers for further care. Individuals with severe anaemia (haemoglobin <82g/L) will be excluded from the randomised intervention (since the intervention might be beneficial in management of anaemia). They will be treated for anaemia and for any underlying cause identified.

Sample handling and archive

Blood and other samples will be processed according to local laboratory standard operating procedures (SOPs). All samples will reach the laboratory in anonymised form.

A sample archive will be developed. Although our current programme of work will address specific hypotheses regarding pathways of effects of parasites and interventions, the sample archive will provide a major asset for exploration of new leads arising from this work, or for an alternative, “systems biology” approach employing (for example) proteomic, genomic, epigenetic and transcriptomic analyses, and investigating the microbiome and virome. Information provided to participants, and consent forms, will include considerations of sample storage, and the possibility of sample analysis in laboratories within and outside Uganda. Participants will be able to decide if they will permit such future use of any leftover samples. We plan to store the samples for up to 20 years. If further storage is needed after that time, permission will be requested from the Uganda Virus Research Institute and London School of Hygiene and Tropical Medicine ethical review committees. If they elect not to permit this, all of those leftover samples will be discarded after the completion of the work included in the current protocol.

Operational considerations

Programme governance

A Programme Steering Committee has been set up to guide progress across all projects. This comprises the following:

- An independent chair
- Representatives from the Ministry of Health programmes for immunisation and for vector borne disease control
- Representatives of district authorities (Mukono and Jinja districts)
- Community representatives
- Principal investigator and co-investigators
- Project leader and post-doctoral immunologist
- Trial statistician
- Laboratory manager

- Medical Research Council observer

Informed consent

Both written informed assent from the participants and written informed consent from a parent or guardian will be required for participation, although these may not necessarily be obtained at the same time. Information will be provided in both English and the appropriate local language. For individuals who cannot speak the languages used, or who cannot read or write, a witness who can read the information sheet and translate the information to the participant or parent/guardian will be used. Two different types of age specific assent forms will be used for the group of participants aged 9 – 12 years and for the group aged 13 – 17 years. Informed consent by emancipated or mature minors will be obtained using a designated consent form for these categories of participants

The aims of the study, all tests, treatments and immunisations to be carried out and potential risks will be explained. The participant will be given the opportunity to ask about details of the trial, and will then have time to consider whether or not to participate. If they do decide to participate, they and their parent/guardian will sign and date two copies of the assent and consent forms, one for them to take away and keep, and one to be stored securely by the research team. Separate information and consent forms will be provided (i) for consent for storage of samples for future studies and for anonymous sharing of data from this study and (ii) for possible genetic studies; the information sheet will explain that these data may be used in analyses related to this protocol.

Screening and Eligibility Assessment

Once the informed consent process has been completed, and consent (and assent) given, a baseline medical history (including concomitant medication) will be collected. Vital signs will be checked and a physical examination will be performed. Inclusion and exclusion criteria will be checked.

Participants will undergo pre- and post-test counselling for HIV and (for girls) pregnancy testing by a trained and experienced nurse- or clinician-counsellor. Blood, urine and stool samples will be obtained, for tests as specified in the schedule of procedures. These tests are to exclude the major, immunomodulating co-infection, HIV, and conditions that might impact safety (anaemia, pregnancy).

Enrolment

Participants who consent/assent, complete the screening processes, satisfy all the inclusion criteria and meet none of the exclusion criteria will be enrolled.

Discontinuation / withdrawal criteria

In accordance with the principles of the current revision of the Declaration of Helsinki and any other applicable regulations, a participant has the right to withdraw from the study at any time and for any reason, and is not obliged to give his or her reasons for doing so. The Investigator may withdraw the

participant at any time in the interests of the participant's health and well-being. In addition, the participant may withdraw/be withdrawn for any of the following reasons:

- Ineligibility (either arising during the study or retrospectively, having been overlooked at screening)
- Administrative decision by the Investigator
- Significant protocol deviation
- Participant non-compliance with study requirements
- An adverse event which requires discontinuation of the study involvement or results in inability to continue to comply with study procedures.

Any participant who becomes pregnant during the trial will be followed up until the end of the pregnancy but no further immunisations will be given unless indicated during pregnancy (as is the case for tetanus toxoid). The trial allocation for this participant will be unblinded and the participant will only be given further treatment if clinically indicated. The babies will also be followed up and examined for any adverse effects. We will not routinely perform venepuncture in a pregnant participant.

The reason for withdrawal will be recorded in the case report form (CRF). If withdrawal is due to an AE, appropriate follow-up visits or medical care will be arranged, with the agreement of the participant, until the AE has resolved, stabilised or a non-trial related causality has been assigned.

If a participant withdraws from the study samples collected before their withdrawal from the trial will be used/ stored unless the participant specifically requests otherwise.

Trial discontinuation

The trial will be discontinued in the event of new scientific information that renders continuation futile or unethical, or for any other reason, at the discretion of the Programme Steering Committee.

End of study definition

The trial will be completed when the last participant enrolled into the trial has completed their final follow up visit.

Safety assessments and oversight

No new investigational drug or product will be used in the proposed trial. However, standard approaches for monitoring safety and reporting of serious adverse events will be followed.

Monitoring

The trial will be monitored by both internal and external monitors according to a pre-defined monitoring plan which will include a site initiation visit, monitoring visits at least annually, and a close-out visit. The monitors will assess patient safety, data integrity, and adherence to the protocol and to Good Clinical Research Practice procedures.

Considerations regarding standard of care

Malaria infection status will be determined retrospectively through assays conducted in bulk on stored samples (malaria PCR). These results will not, therefore, be useful to determine management of individual participants.

Participants in the placebo arms will receive lower levels of anti-malaria treatment. However, all trial arms will receive a minimum of well-implemented national standard of care.

Dihydroartemisinin/piperaquine is considered an attractive option for preventive treatment and preventive chemotherapy for malaria because of the long half-life of piperaquine (approximately 23 days).[32] Monthly treatment with DP has been shown to reduce the prevalence of anaemia and reduce episodes of clinical malaria in Ugandan schools[33] but has not been adopted as standard of care. This programme is expected to add further evidence regarding the potential benefits of monthly DP for school children by determining the effect on vaccine responses, thereby further contributing to policy debate in this field. To manage the expected differential benefits of the interventions for anaemia, a full blood count will be performed at baseline, as discussed above; anaemic children will be managed appropriately and severely anaemic children excluded.

Malaria standard of care will comprise provision of bed nets to minimise malaria exposure for all participants. Rapid diagnostic tests and treatment will be made readily available for participants who develop symptomatic malaria.

Albendazole will be provided twice a year to manage nematode infections (after collection of primary and secondary endpoint samples).

Procedures to be followed in the event of abnormal findings

Abnormal clinical findings from medical history, examination or blood tests will be assessed as to their clinical significance throughout the trials. If an abnormal test result is deemed clinically significant, it may be repeated. If a test remains clinically significant, the participant will be informed and appropriate medical care arranged as appropriate and with the permission of the participant. Specific details regarding findings, discussion with participants and resulting actions will be recorded in the clinical records. Decisions to exclude the participant from enrolling in the trial or to withdraw a participant from the trial will be at the discretion of the Investigator.

Data and Safety Monitoring Board (DSMB)

A data and safety monitoring board (DSMB) will be appointed to provide real-time safety oversight. The DSMB will be notified within 7 days of the Investigators' being aware of the occurrence of SAEs. The DSMB may recommend the Investigators to place the trial on hold if deemed necessary following an intervention-related SAE. The DSMB will be chaired by a clinician experienced in clinical trials. There will be a minimum

of two other appropriately qualified committee members. In the case of events related to a blinded intervention, the DSMB can request unblinding. Membership will include a statistician, and at least one Ugandan member. All correspondence between Investigators and the DSMB will be conveyed by the Principal Investigator to the trial Sponsor. The Chair of the DSMB will be contacted for advice and independent review by the Investigator or trial Sponsor in the following situations:

- The occurrence of any SAE
- Any other situation where the Investigator or trial Sponsor feels independent advice or review is important

Ethical and regulatory considerations

Further information regarding risks

The immunisations to be given have recognised side effects which are usually mild and resolve spontaneously in a few days to one week. Parenteral vaccines are likely to result in pain and swelling at the site of injection and mild fever; very occasionally pain and swelling can be severe and associated with difficulty in moving the shoulder. Sometimes headache and tiredness occur. Rarely a vaccine may cause a severe allergic reaction. For most vaccines this is estimated at less than one in a million doses (but 1 in 55,000 for Yellow Fever vaccine).[34] Individuals with a history of a possible allergic reaction to drugs or vaccines, or to vaccine components including eggs or chicken proteins, will be excluded from the studies. The research team will be trained and prepared to manage severe allergic reactions.

Adverse reactions to Yellow Fever vaccine include severe nervous system reaction (about 1 person in 125,000) and severe, life-threatening illness with organ failure (about 1 person in 250,000). The mortality for this severe, life-threatening adverse effect is reported as about 50%.[34]

BCG immunisation is likely to induce a scar in many cases. This may develop over several weeks, starting as a small papule at the injection site which may become ulcerated and then heal over a period of 2 to 5 months; and lymphadenopathy may develop. Occasionally a more severe local reaction occurs (estimated at 1 per 1,000-10,000 doses): for example, an abscess develops and scars may develop into keloids. Rarely BCG can cause disseminated disease (1 per 230,000 to 640,000 doses), or disease in sites remote from the immunisation site. Disseminated BCG disease usually occurs in immunocompromised people: HIV positive people will be excluded from these studies.[35] BCG “pre-immunisation” may interfere with the response to the subsequent live vaccines; indeed our hypothesis, and published results, suggest that it may suppress replication of YF 17D vaccine.[36] However, this reduced replication has not been shown to correlate with, or result in, reduced levels of neutralising antibody titres (which are the desired protective outcome).[13 36]

Oral typhoid vaccine (Ty21a) may occasionally be associated with stomach pain, nausea, vomiting and (rarely) rash.[34]

Dihydroartemisinin/piperaquine is generally well tolerated and has been used widely in practice, as well as in a substantial number of trials.[32] The main potential adverse effect of concern is cardiac toxicity, particularly dose-dependent prolongation of the QTc interval and associated increased risk of arrhythmias. There has been concern that provision of multiple doses might increase this risk. However, a recent meta-analysis involving 11 trials of DP (9 IPT, 2 treatment trials) involving 14,628 participants, 3,935 of whom received multiple treatments with DP found no evidence to suggest significant cardiac toxicity.[32] Few of the studies measured electrocardiographic changes, but those that did so found no increase in QTc intervals with increasing numbers of doses.

Benefits

All the treatments and vaccines to be given are licensed and regarded as safe. In general, the vaccines and treatments are expected to provide protection against infectious diseases.

With regard to BCG immunisation or revaccination in adolescence, benefits with respect to protection against tuberculosis among Ugandan adolescents are unknown and may, at best, be modest. There may be non-specific benefits. WHO's SAGE committee concluded, in their summary of October 2017,[37] that "BCG revaccination is safe in *Mycobacterium tuberculosis* infected and uninfected populations. There is a lack of evidence from randomized controlled trials and retrospective cohort and case-control studies demonstrating the efficacy and effectiveness of BCG revaccination in adolescents and adults after primary BCG vaccination in infancy for protection against TB disease. Due to absence of evidence, BCG revaccination is not considered cost-effective. Further research is warranted to explore whether certain sub-groups of age, geographic or M. tuberculosis exposure categories would benefit from revaccination." We hope, through this work, to contribute to this debate.

Participants and their families, schools and communities are expected to benefit from improved understanding of malaria, and of vaccines.

REFERENCES

1. Wajja A, Kizito D, Nassanga B, et al. The effect of current Schistosoma mansoni infection on the immunogenicity of a candidate TB vaccine, MVA85A, in BCG-vaccinated adolescents: An open-label trial. *PLoS neglected tropical diseases* 2017;11(5):e0005440. doi: 10.1371/journal.pntd.0005440 [published Online First: 2017/05/05]

2. WHO. Growth reference 5-19 years. 2007

3. WHO. Global tuberculosis report 2016 http://www.who.int/tb/publications/global_report/en/ (accessed 17 June 2017), 2016.

4. Alcais A, Fieschi C, Abel L, et al. Tuberculosis in children and adults: two distinct genetic diseases. *J Exp Med* 2005;202(12):1617-21. doi: 10.1084/jem.20052302

5. Weiner J, 3rd, Kaufmann SH. Recent advances towards tuberculosis control: vaccines and biomarkers. *J Intern Med* 2014;275(5):467-80. doi: 10.1111/joim.12212

6. Fine PE. Variation in protection by BCG: implications of and for heterologous immunity. *Lancet* 1995;346(8986):1339-45.

7. Barreto ML, Pilger D, Pereira SM, et al. Causes of variation in BCG vaccine efficacy: examining evidence from the BCG REVAC cluster randomized trial to explore the masking and the blocking hypotheses. *Vaccine* 2014;32(30):3759-64. doi: 10.1016/j.vaccine.2014.05.042

8. Black GF, Weir RE, Floyd S, et al. BCG-induced increase in interferon-gamma response to mycobacterial antigens and efficacy of BCG vaccination in Malawi and the UK: two randomised controlled studies. *Lancet* 2002;359(9315):1393-401. doi: 10.1016/S0140-6736(02)08353-8

9. Tanner R, Kakalacheva K, Miller E, et al. Serum indoleamine 2,3-dioxygenase activity is associated with reduced immunogenicity following vaccination with MVA85A. *BMC infectious diseases* 2014;14:660. doi: 10.1186/s12879-014-0660-7 [published Online First: 2014/12/04]

10. WHO. SAGE Evidence to recommendations framework. 2017. http://www.who.int/immunization/sage/meetings/2017/october/2_EvidencetoRecommendationFramework_BCG.pdf (accessed 16th March 2018).

11. Dye C. Making wider use of the world's most widely used vaccine: Bacille Calmette-Guerin revaccination reconsidered. *Journal of the Royal Society, Interface* 2013;10(87):20130365. doi: 10.1098/rsif.2013.0365 [published Online First: 2013/08/02]

12. Kraemer MU, Faria NR, Reiner RC, Jr., et al. Spread of yellow fever virus outbreak in Angola and the Democratic Republic of the Congo 2015-16: a modelling study. *The Lancet Infectious diseases* 2016 doi: 10.1016/s1473-3099(16)30513-8 [published Online First: 2016/12/27]

13. Muyanja E, Ssemaganda A, Ngauv P, et al. Immune activation alters cellular and humoral responses to yellow fever 17D vaccine. *The Journal of clinical investigation* 2014;124(7):3147-58. doi: 10.1172/jci75429 [published Online First: 2014/06/10]

14. Aguiar M, Stollenwerk N, Halstead SB. The Impact of the Newly Licensed Dengue Vaccine in Endemic Countries. *PLoS neglected tropical diseases* 2016;10(12):e0005179. doi: 10.1371/journal.pntd.0005179 [published Online First: 2016/12/22]

15. Dharmasena MN, Osorio M, Filipova S, et al. Stable expression of Shigella dysenteriae serotype 1 O-antigen genes integrated into the chromosome of live Salmonella oral vaccine vector Ty21a. *Pathogens and disease* 2016 doi: 10.1093/femspd/ftw098 [published Online First: 2016/09/23]

16. Carias C, Walters MS, Wefula E, et al. Economic evaluation of typhoid vaccination in a prolonged typhoid outbreak setting: the case of Kasese district in Uganda. *Vaccine* 2015;33(17):2079-85. doi: 10.1016/j.vaccine.2015.02.027 [published Online First: 2015/02/26]

17. Melhem RF, LoVerde PT. Mechanism of interaction of Salmonella and Schistosoma species. *Infection and immunity* 1984;44(2):274-81. [published Online First: 1984/05/01]

18. Muniz-Junqueira MI, Tavares-Neto J, Prata A, et al. Antibody response to Salmonella typhi in human schistosomiasis mansoni. *Revista da Sociedade Brasileira de Medicina Tropical* 1996;29(5):441-5. [published Online First: 1996/09/01]

19. WHO. Position Paper on Typhoid vaccines: WHO position paper – March 2018 2018

20. Brown J, Baisley K, Kavishe B, et al. Impact of malaria and helminth infections on immunogenicity of the human papillomavirus-16/18 AS04-adjuvanted vaccine in Tanzania. *Vaccine* 2014;32(5):611-7. doi: 10.1016/j.vaccine.2013.11.061

21. Riner DK, Ndombi EM, Carter JM, et al. Schistosoma mansoni Infection Can Jeopardize the Duration of Protective Levels of Antibody Responses to Immunizations against Hepatitis B and Tetanus Toxoid. *PLoS neglected tropical diseases* 2016;10(12):e0005180. doi: 10.1371/journal.pntd.0005180

22. Centre HI. HPV and related diseases report: Uganda. 2016. http://www.hpvcentre.net/statistics/reports/UGA.pdf (accessed 20.01.2017).

23. WHO. Human papillomavirus vaccines: WHO position paper, May 2017. *Releve epidemiologique hebdomadaire* 2017;92(19):241-68. [published Online First: 2017/05/23]

24. Nanteza B, Galukande M, Aceng J, et al. The burden of tetanus in Uganda. *SpringerPlus* 2016;5(1):705. doi: 10.1186/s40064-016-2309-z [published Online First: 2016/06/29]

25. Petralli JK, Merigan TC, Wilbur JR. ACTION OF ENDOGENOUS INTERFERON AGAINST VACCINIA INFECTION IN CHILDREN. *Lancet* 1965;2(7409):401-5. [published Online First: 1965/08/28]

26. C-A S. Vaccine Immunology. In: Plotkin SA, Orenstein W, Offit P, et al., eds. Vaccines: Elsevier 2017.

27. PHE. Public Health England: revised recommendations for the administration of more than one

live vaccine 2015

28. PHE. Immunization procedures. The Green Book. Public Health England. 2012

29. PHE. Public Health England: revised recommendations for the administration of more than one live vaccine 2015

30. Ministry of Health–Uganda. Uganda Clinical Guidelines 2016. http://apps.who.int/medicinedocs/documents/s23532en/s23532en.pdf

31. WHO. Haemoglobin concentrations for the diagnosis of anaemia and assessment of severity. Vitamin and Mineral Nutrition Information System. Geneva, World Health Organization, 2011 (WHO/NMH/NHD/MNM/11.1).

32. Gutman J, Kovacs S, Dorsey G, et al. Safety, tolerability, and efficacy of repeated doses of dihydroartemisinin-piperaquine for prevention and treatment of malaria: a systematic review and meta-analysis. *The Lancet Infectious diseases* 2017;17(2):184-93. doi: 10.1016/s1473-3099(16)30378-4 [published Online First: 2016/11/21]

33. Nankabirwa JI, Wandera B, Amuge P, et al. Impact of intermittent preventive treatment with dihydroartemisinin-piperaquine on malaria in Ugandan schoolchildren: a randomized, placebo-controlled trial. *Clinical infectious diseases : an official publication of the Infectious Diseases Society of America* 2014;58(10):1404-12. doi: 10.1093/cid/ciu150 [published Online First: 2014/03/14]

34. CDC. Centers for Disease Control and Prevention, vaccines and immunizations.

35. WHO. Information sheet observed rate of vaccine reactions Bacille Calmette-Guérin (BCG) vaccine. 2012

36. Arts RJW, Moorlag S, Novakovic B, et al. BCG Vaccination Protects against Experimental Viral Infection in Humans through the Induction of Cytokines Associated with Trained Immunity. *Cell host & microbe* 2018;23(1):89-100.e5. doi: 10.1016/j.chom.2017.12.010 [published Online First: 2018/01/13]

37. WHO. Immunization, vaccines and biologicals. Vaccine position papers. 2017
