# Supplementary material for: Effect of intermittent preventive treatment for malaria with dihydroartemisinin-piperaquine on immune responses to vaccines among rural Ugandan adolescents: randomised controlled trial protocol B for the ‘POPulation differences in VACcine responses’ (POPVAC) programme
Source: BMJ Open. 2021 Feb 16;11(2):e040427. doi: 10.1136/bmjopen-2020-040427 (PMC7893608; doi:10.1136/bmjopen-2020-040427)
Supplement: Author's manuscript [file bmjopen-2020-040427.draft_revisions.pdf]

BMJ Open is committed to open peer review. As part of this commitment we make the peer review history of every article we publish publicly available.

When an article is published we post the peer reviewers' comments and the authors' responses online. We also post the versions of the paper that were used during peer review. These are the versions that the peer review comments apply to.

The versions of the paper that follow are the versions that were submitted during the peer review process. They are not the versions of record or the final published versions. They should not be cited or distributed as the published version of this manuscript.

BMJ Open is an open access journal and the full, final, typeset and author-corrected version of record of the manuscript is available on our site with no access controls, subscription charges or pay-per-view fees (http://bmjopen.bmj.com).

If you have any questions on BMJ Open's open peer review process please email info.bmjopen@bmj.com

BMJ Open

The effect of intermittent preventive treatment for malaria with dihydroartemisinin-piperaquine on immune responses to vaccines among rural Ugandan adolescents: randomised controlled trial protocol B for the 'POPulation differences in VACcine responses' (POPVAC) programme

Journal:	*BMJ Open*
Manuscript ID	bmjopen-2020-040427
Article Type:	Protocol
Date Submitted by the Author:	13-May-2020

Complete List of Authors:	Natukunda , Agnes ; Medical Research Council/Uganda Virus Research Institute and London School of Hygiene and Tropical Medicine (MRC/UVRI and LSHTM) Uganda Research Unit, Immunomodulation and Vaccines Programme<br>Nkurunungi, Gyaviira; Medical Research Council/Uganda Virus Research Institute and London School of Hygiene and Tropical Medicine (MRC/UVRI and LSHTM) Uganda Research Unit, Immunomodulation and Vaccines Programme<br>Zirimenya , Ludoviko ; Medical Research Council/Uganda Virus Research Institute and London School of Hygiene and Tropical Medicine (MRC/UVRI and LSHTM) Uganda Research Unit, Immunomodulation and Vaccines Programme<br>Nassuuna , Jacent; Medical Research Council/Uganda Virus Research Institute and London School of Hygiene and Tropical Medicine (MRC/UVRI and LSHTM) Uganda Research Unit, Immunomodulation and Vaccines Programme<br>Oduru, Gloria; Medical Research Council/Uganda Virus Research Institute and London School of Hygiene and Tropical Medicine (MRC/UVRI and LSHTM) Uganda Research Unit, Immunomodulation and Vaccines Programme<br>Amongin , Rebecca ; Medical Research Council/Uganda Virus Research Institute and London School of Hygiene and Tropical Medicine (MRC/UVRI and LSHTM) Uganda Research Unit, Immunomodulation and Vaccines Programme<br>Kabuubi , Prossy ; Medical Research Council/Uganda Virus Research Institute and London School of Hygiene and Tropical Medicine (MRC/UVRI and LSHTM) Uganda Research Unit, Immunomodulation and Vaccines Programme<br>Mutebe , Alex ; Medical Research Council/Uganda Virus Research Institute and London School of Hygiene and Tropical Medicine (MRC/UVRI and LSHTM) Uganda Research Unit, Immunomodulation and Vaccines Programme<br>Onen, Caroline; Medical Research Council/Uganda Virus Research Institute and London School of Hygiene and Tropical Medicine (MRC/UVRI and LSHTM) Uganda Research Unit, Immunomodulation and Vaccines Programme<br>Amongi, Susan; Medical Research Council/Uganda Virus Research

Institute and London School of Hygiene and Tropical Medicine (MRC/UVRI and LSHTM) Uganda Research Unit, Immunomodulation and Vaccines Programme
Nakazibwe, Esther; Medical Research Council/Uganda Virus Research Institute and London School of Hygiene and Tropical Medicine (MRC/UVRI and LSHTM) Uganda Research Unit, Immunomodulation and Vaccines Programme
Akello , Florence ; Medical Research Council/Uganda Virus Research Institute and London School of Hygiene and Tropical Medicine (MRC/UVRI and LSHTM) Uganda Research Unit, Immunomodulation and Vaccines Programme
Kiwanuka, Samuel; Medical Research Council/Uganda Virus Research Institute and London School of Hygiene and Tropical Medicine (MRC/UVRI and LSHTM) Uganda Research Unit, Immunomodulation and Vaccines Programme
Kiwudhu, Fred; Medical Research Council/Uganda Virus Research Institute and London School of Hygiene and Tropical Medicine (MRC/UVRI and LSHTM) Uganda Research Unit, Immunomodulation and Vaccines Programme
Sewankambo, Moses; Medical Research Council/Uganda Virus Research Institute and London School of Hygiene and Tropical Medicine (MRC/UVRI and LSHTM) Uganda Research Unit, Immunomodulation and Vaccines Programme
Nsubuga, Denis; Medical Research Council/Uganda Virus Research Institute and London School of Hygiene and Tropical Medicine (MRC/UVRI and LSHTM) Uganda Research Unit, Immunomodulation and Vaccines Programme
Kizindo , Robert ; Medical Research Council/Uganda Virus Research Institute and London School of Hygiene and Tropical Medicine (MRC/UVRI and LSHTM) Uganda Research Unit, Immunomodulation and Vaccines Programme
Staedke, Sarah; London School of Hygiene and Tropical Medicine, Faculty of Infectious and Tropical Diseases; Infectious Disease Research Collaboration,
Cose , Stephen ; Medical Research Council/Uganda Virus Research Institute and London School of Hygiene and Tropical Medicine (MRC/UVRI and LSHTM) Uganda Research Unit, Immunomodulation and Vaccines Programme; London School of Hygiene and Tropical Medicine Department of Clinical Research
Webb, Emily; London School of Hygiene and Tropical Medicine, MRC Tropical Epidemiology Group, Department of Infectious Disease Epidemiology
Elliott, Alison ; Medical Research Council/Uganda Virus Research Institute and London School of Hygiene and Tropical Medicine (MRC/UVRI and LSHTM) Uganda Research Unit, Immunomodulation and Vaccines Programme; London School of Hygiene and Tropical Medicine Department of Clinical Research

Keywords: Infection control < INFECTIOUS DISEASES, PARASITOLOGY, Public health < INFECTIOUS DISEASES, Immunology < TROPICAL MEDICINE, Epidemiology < TROPICAL MEDICINE, Paediatric infectious disease & immunisation < PAEDIATRICS

SCHOLARONE™
Manuscripts

I, the Submitting Author has the right to grant and does grant on behalf of all authors of the Work (as defined in the below author licence), an exclusive licence and/or a non-exclusive licence for contributions from authors who are: i) UK Crown employees; ii) where BMJ has agreed a CC-BY licence shall apply, and/or iii) in accordance with the terms applicable for US Federal Government officers or employees acting as part of their official duties; on a worldwide, perpetual, irrevocable, royalty-free basis to BMJ Publishing Group Ltd ("BMJ") its licensees and where the relevant Journal is co-owned by BMJ to the co-owners of the Journal, to publish the Work in this journal and any other BMJ products and to exploit all rights, as set out in our licence.

The Submitting Author accepts and understands that any supply made under these terms is made by BMJ to the Submitting Author unless you are acting as an employee on behalf of your employer or a postgraduate student of an affiliated institution which is paying any applicable article publishing charge ("APC") for Open Access articles. Where the Submitting Author wishes to make the Work available on an Open Access basis (and intends to pay the relevant APC), the terms of reuse of such Open Access shall be governed by a Creative Commons licence – details of these licences and which Creative Commons licence will apply to this Work are set out in our licence referred to above.

Other than as permitted in any relevant BMJ Author's Self Archiving Policies, I confirm this Work has not been accepted for publication elsewhere, is not being considered for publication elsewhere and does not duplicate material already published. I confirm all authors consent to publication of this Work and authorise the granting of this licence.

For peer review only

The effect of intermittent preventive treatment for malaria with dihydroartemisinin-piperaquine on immune responses to vaccines among rural Ugandan adolescents: randomised controlled trial protocol B for the 'POPulation differences in VACcine responses' (POPVAC) programme

Agnes Natukunda[1,¶], Gyaviira Nkurunungi[1,¶,*], Ludoviko Zirimenya[1,¶], Jacent Nassuuna[1], Gloria Oduru[1], Rebecca Amongin[1], Prossy N Kabuubi[1], Alex Mutebe[1], Caroline Onen[1], Susan Amongi[1], Esther Nakazibwe[1], Florence Akello[1], Samuel Kiwanuka[1], Fred Kiwudhu[1], Moses Sewankambo[1], Denis Nsubuga[1], Robert Kizindo[1], Sarah G Staedke[2,3], Stephen Cose[1,3], Emily L Webb[4], Alison M Elliott[1,3] **for the POPVAC trial team**

[1]Immunomodulation and Vaccines Programme, Medical Research Council/Uganda Virus Research Institute and London School of Hygiene and Tropical Medicine (MRC/UVRI and LSHTM) Uganda Research Unit, Entebbe, Uganda

[2]Infectious Diseases Research Collaboration, Kampala, Uganda

[3]Department of Clinical Research, London School of Hygiene and Tropical Medicine, London, United Kingdom

[4]MRC Tropical Epidemiology Group, Department of Infectious Disease Epidemiology, London School of Hygiene and Tropical Medicine, London, United Kingdom

¶These authors contributed equally

***Correspondence:** Gyaviira Nkurunungi; gyaviira.nkurunungi@lshtm.ac.uk

For peer review only

Abstract

Introduction

Drivers of lower vaccine efficacy and impaired vaccine-specific immune responses in low-income versus high-income countries, and in rural, compared to urban settings, are not fully elucidated. Repeated exposure to and immunomodulation by parasite infections may be important. We focus on *Plasmodium falciparum* malaria, aiming to determine whether there are reversible effects of malaria infection on vaccine responses.

Methods and analysis

We have designed a randomised, double-blind, placebo-controlled, parallel group trial of preventive malaria treatment versus placebo, to determine effects on vaccine response outcomes among school-going adolescents (9 to 17 years) from malaria-endemic rural areas of Jinja district (Uganda). Vaccines to be studied comprise BCG vaccine on day 'zero'; yellow fever, oral typhoid and Human Papilloma Virus vaccines at week 4; and Tetanus/diphtheria booster vaccine at week 28. Participants in the preventive malaria treatment arm will receive dihydroartemisinin piperaquine (DP) dosed by weight, one month apart, prior to the first immunisation, followed by monthly treatment thereafter. We expect to enrol 640 adolescents.

Primary outcomes are BCG-specific IFN-γ ELISpot responses eight weeks after BCG immunisation and for other vaccines, antibody responses to key vaccine antigens at four weeks after immunisation. In secondary analyses, we will determine effects of monthly DP treatment (versus placebo) on correlates of protective immunity, on vaccine response waning, on whether there are differential effects on priming vs boosting immunisations, and on malaria infection prevalence. We will also conduct exploratory immunology assays among subsets of participants to further characterise effects of the intervention on vaccine responses.

Ethics and dissemination

Ethics approval has been obtained from relevant Ugandan and UK ethics committees. Results will be shared with Uganda Ministry of Health, relevant district councils, community leaders and study participants. Further dissemination will be done through conference proceedings and publications.

Trial registration

Current Controlled Trials identifier: ISRCTN62041885

Article summary

Strengths and limitations of this study

- This will be the first well-powered trial to evaluate the effect of repeated intermittent presumptive anti-malarial treatment on vaccine response outcomes in adolescents.

For peer review only - http://bmjopen.bmj.com/site/about/guidelines.xhtml

- Effects on both live-attenuated and inert vaccines will be studied.
- The use of an interventional research design will contribute strong evidence to determine whether malaria has causal and reversible effects on vaccine response outcomes, and our strong immunoepidemiological design and nested immunological studies will make it possible to address specific hypotheses regarding pathways of effects.
- The use of vaccines most of which are part of Uganda's national Expanded Programme on Immunisation (EPI), makes the findings from this study applicable and could inform the introduction of combined school health programmes for malaria control and vaccine provision.
- With the high prevalence of malaria in the proposed study area, participants in the placebo arm may notice more frequent episodes of malaria and this could lead to individual self-unblinding and self-treatment, compromising the design of the study.

Word count

3407

Keywords

Malaria; Vaccine; Dihydroartemisinin; Immunization

For peer review only

For peer review only - http://bmjopen.bmj.com/site/about/guidelines.xhtml

Introduction

Population differences in vaccine-specific immune responses and efficacy have been documented. In tropical low-income, rural settings, responses to both live[1-6] and non-live[7 8] vaccines are often impaired, compared to urban and high-income country settings. Studies have also shown that responses to candidate vaccines (such as those to tuberculosis,[9] malaria[10] and Ebola[11]) are lower in Africa than in Europe or America. Drivers of these population differences in vaccine response are incompletely understood. While genetic differences may play a role, they cannot fully explain this phenomenon: for example, migrant and native English populations have comparable BCG vaccine efficacy.[12] Previous exposure to the pathogen targeted by the vaccine, or to related organisms, may mask the benefit of the vaccine;[13 14] however, this mechanism cannot explain observations for vaccines against rare organisms such as Ebola.[11] The role of immunomodulating environmental factors has been discussed,[2] but evidence is inconclusive. Parasites, particularly, have long been proposed as modulators of vaccine responses,[15] but this has not been fully substantiated in well-powered trials aimed at evaluating reversibility of their effects in human populations.[16]

The focus of this POPVAC B trial is on infection with malaria parasites. Malaria remains highly prevalent in Africa; emerging insecticide and drug resistance threatens gains towards control.[17] Interventions like intermittent preventive treatment (IPT) are recommended by the World Health Organisation for use in preventing malaria in pregnant women, infants, and young children living in areas of seasonal malaria transmission.[17] In a recently concluded trial in Uganda, IPT in schoolchildren was shown to be beneficial in protecting individual children and reducing community transmission of malaria.[18 19] In a 2010 review, Cunnington and Riley showed that, despite long-standing interest, the impact of malaria on vaccine responses was not adequately understood.[20] Although there is little evidence that malaria impairs responses to protein vaccines given in multiple doses,[20-22] they found consistent evidence of an adverse effect on the response to polysaccharide vaccines.[20]

Malaria may impact vaccine responses due to acute immunological changes (associated with fever)[23], or by longer term effects (for example on T follicular helper cell and B cell function).[24] The extent to which infection with malaria impacts immunological characteristics associated with vaccine responses may best be determined by intervention studies. This trial protocol B of the 'Population differences in Vaccine responses" programme has been designed to evaluate the effect of preventive treatment of malaria on vaccine responses. We summarise the protocol here. This study is one of three parallel trials whose designs and cross-cutting analyses are described separately in this issue. Understanding the predictors of vaccine responses, and the factors that drive them, will contribute to finding ways of improving vaccine efficacy for rural, tropical settings.

For peer review only - http://bmjopen.bmj.com/site/about/guidelines.xhtml

99 **Hypothesis**

100 The overarching goal of the POPVAC programme is to understand population differences in vaccine
101 responses in Uganda, in order to identify strategies through which vaccine effectiveness can be optimised
102 for the low-income, tropical settings where they are especially needed. For this Trial B, we focus on the
103 hypothesis *that malaria infection suppresses responses to unrelated vaccines, and that this effect can be*
104 *reversed, at least in part, by monthly preventive treatment of malaria in schools in high transmission*
105 *settings.*

106 **Objective**

107 To determine whether there are reversible effects of malaria infection on vaccine response in adolescents,
108 using an intervention study.

For peer review only

For peer review only - http://bmjopen.bmj.com/site/about/guidelines.xhtml

Methods and analysis

Design, setting and participants

SPIRIT reporting guidelines[25] are used. We will conduct an individually randomised, double-blind, placebo-controlled, parallel group trial of monthly dihydroartemisinin/piperaquine (DP) versus placebo among primary school children in rural schools in Jinja district, Uganda. We will recruit 640 participants, randomising 320 to each trial arm. The study cohort will recruit participants aged 9 to 17 years from primary school year 1 up to 6 (to avoid primary leaving examinations in late year 7, and loss to follow up of children who leave from primary 7). Schools will be selected purposefully to assure malaria prevalence appropriate to our design. The study setting is remote from Lake Victoria and the River Nile, in order to minimise exposure to schistosomiasis.

Recruitment criteria

Inclusion criteria

i. Attending the selected school and planning to continue to attend the school for the duration of the study
ii. Aged 9 to 17 years and enrolled in primary 1 to 6
iii. Written informed assent by participant and consent by parent or guardian
iv. Agree to avoid pregnancy for the duration of the trial (female only)
v. Willing to provide locator information and to be contacted during the course of the trial
vi. Able and willing (in the investigator's opinion) to comply with all the study requirements

Exclusion criteria

i. Clinically significant history of immunodeficiency (including HIV), cancer, cardiovascular disease, gastrointestinal disease, liver disease, renal disease, endocrine disorder or neurological illness
ii. Moderate or severe acute illness characterised by any of the following symptoms: fever, impaired consciousness, convulsions, difficulty in breathing, vomiting; or as determined by the attending project clinician.
iii. Family history of sudden death attributable to heart condition in a first degree relative
iv. Family history of long QT syndrome
v. Know congenital prolongation of the corrected QT (QTc) interval
vi. History of known heart disease or fainting
vii. Known allergy or history of adverse reaction to DP or to artemether-lumefantrine
viii. History of serious psychiatric condition or disorder
ix. Previous immunisation with Yellow Fever (YF), oral typhoid or Human Papillomavirus (HPV) vaccine; previous immunisation with BCG or Tetanus and diphtheria vaccine (Td) at age ≥5 years

For peer review only

For peer review only - http://bmjopen.bmj.com/site/about/guidelines.xhtml

x. Concurrent oral or systemic steroid medication or the concurrent use of other immunosuppressive agents within 2 months prior to enrolment

xi. Current use of medications known to prolong the QT interval

xii. History of allergic reaction to immunisation or any allergy likely to be exacerbated by any component of the study vaccines including egg or chicken proteins

xiii. Tendency to develop keloid scars

xiv. Haemoglobin less than 80g/L

xv. Positive HIV serology

xvi. Positive pregnancy test

xvii. Female currently lactating, confirmed pregnancy or intention to become pregnant during the trial period

xviii. Use of an investigational medicinal product or non-registered drug, live vaccine, or medical device other than the study vaccines for 30 days prior to dosing with the study vaccine, or planned use during the study period

xix. Administration of immunoglobulins and/or any blood products within the three months preceding the planned trial immunisation date.

Further information on recruitment criteria can be found in Supplementary information.

Interventions

We will individually randomise participants in a 1:1 ratio to monthly dihydroartemisinin-piperaquine (DP) versus a DP-matching placebo in a double-blind, placebo-controlled trial (**Figure 1** and Supplementary **Table S1**). DP has been shown to reduce prevalence of asymptomatic malaria by 94% and malaria incidence by 96% among Ugandan school children.[26] The DP treatment arm will receive two doses, one month apart, prior to the first immunisation, followed by monthly treatment thereafter. Each dose will be calculated based on the participant's weight in kilograms.[27] DP (Dihydroartemisinin 40mg + Piperaquine Phosphate 320mg) tablets will be administered once a day for three consecutive days. The standard arm will receive placebo since, as yet, routine preventive malaria treatment in schools is not Uganda Ministry of Health policy.

Randomisation and allocation to treatment arm

An independent statistician will generate the randomisation code using a randomly permuted block size. This code will be embedded into a web-based randomisation system in REDCap (Research Electronic Data Capture) software.[28 29] At enrolment, eligibility criteria will be checked and eligible participants will be allocated sequentially to the next randomisation number, with the corresponding trial arm designated in REDCap. The randomisation code will be kept securely by the trial statistician with a second copy held by a

For peer review only

data manager or statistician not otherwise involved in the trial at the MRC/UVRI and LSHTM Uganda Research Unit.

Blinding

The trial will be double-blind, and will remain so until data collection and data cleaning are complete and data locked for analysis, unless unblinding is required for individual participants who become pregnant (for these cases unblinding can be requested by the Principal Investigator) or as a result of serious adverse events or reactions (at the request of the Data and Safety Monitoring Board).

Immunisations

We will study a portfolio of licensed vaccines (live and inert, oral and parental, priming and boosting) expected to be beneficial (in some cases, already given) to adolescents in Uganda. Our schedule (**Table 1** and **Table S1**) will comprise three main immunisation days (week 0, week 4 and week 28). Additional HPV immunisation will be provided for girls aged 14 years or above, and a second Tetanus/diphtheria boost will be given after completion of the study, to accord with the national Expanded Programme on Immunisation (EPI) routines but the response to these will not specifically be addressed. Further rationale for the selection of vaccines is detailed in Supplementary information. Our schedule has been developed in consultation with the EPI programme and is cognisant of potential interference between vaccines (Supplementary information).

Table 1. Immunisation schedule

	Immunisation week 0	Immunisation week 4	[Immunisation week 8]	Immunisation week 28	[Immunisation week 52]
**Live vaccines**	BCG vaccination / re-vaccination[1]	Yellow fever (YF-17D)<br>Oral typhoid (Ty21a)			
**Non-live vaccines**		HPV prime	HPV boost for girls aged $\geq$14 years[2,3]	HPV boost and Tetanus/ diphtheria (Td) boost	Tetanus/ diphtheria (Td) boost[3,4]

1. Prior BCG status may vary (data on history and documentation of prior BCG, and presence of a BCG scar, will be documented although these approaches have limitations for determining BCG status)
2. The National EPI programme recommends three doses of HPV vaccine for older girls
3. These doses will be given to comply with guidelines but outcomes specifically relating to these doses will not be assessed
4. Priming by immunisation in infancy is assumed

Schedule of immunisation and sampling

The schedule of immunisation and sampling is outlined in **Figure 1** and **Table S1**. While optimal timings for outcome measures vary between vaccines, sampling at 8 weeks post BCG and 4 weeks post YF-17D, Ty21a, HPV and Td is proposed for the primary endpoints, targeting the establishment of memory responses and approximate peak of antibody responses. A secondary endpoint at one year will assess waning. All analyses will take baseline measurements into account. Immunisation postponement criteria are detailed in Supplementary information.

For peer review only - http://bmjopen.bmj.com/site/about/guidelines.xhtml

Outcomes

Primary outcomes

These will be assessed in all participants.

i. **BCG:** BCG-specific IFN-γ ELISpot response eight weeks post BCG immunisation.
ii. **YF-17D:** neutralising antibody titres (plaque-reduction neutralisation test) at four weeks post YF immunisation.
iii. **Ty21a:** *Salmonella typhi* lipopolysaccharide (LPS)-specific immunoglobulin (Ig)G concentration at four weeks post Ty21a immunisation.
iv. **HPV:** IgG specific for L1-proteins of HPV-16/18 at four weeks post HPV priming immunisation.
v. **Td:** Tetanus and diphtheria toxoid-specific IgG concentration at four weeks post Td immunisation.

Secondary outcomes

These will be assessed in all participants and will further investigate estimates of protective immunity (for vaccines where these are available) and dynamics of the vaccine responses, as well as the impact of the interventions on parasite clearance.

i. **Protective immunity.** Proportions with protective neutralising antibody (YF); protective IgG levels (TT);[30] seroconversion rates (Ty21a) at four weeks post the corresponding immunisation.
ii. **Response waning.** Primary outcome measures (all vaccines) repeated at week 52, and area-under-the curve (AUC) analyses.
iii. **Priming versus boosting.** Effects on priming versus boosting will be examined for HPV only, comparing outcomes four weeks after the first, and four weeks after the second vaccine dose.
iv. **Current malaria infection status and intensity** will be assessed retrospectively by PCR on stored samples collected on immunisation days and at week 52.

Furthermore, our sample collection will offer opportunities for an array of exploratory immunological evaluations on stored samples, focusing mainly on vaccine antigen specific outcomes. Exploratory assessments will provide further detail on immune response characteristics over the study time-course, and on the role of immunological profiles in malaria-mediated modulation of vaccine-specific responses.

Additional evaluation of parasite infection

1. **Current *S. mansoni* infection status and intensity** will be determined by serum/plasma levels of circulating anodic antigen (CAA). This method is quantitative, highly specific for *Schistosoma* infection, and much more sensitive than the conventional Kato Katz method.[31] CAA will be assessed retrospectively on stored samples collected at baseline and at weeks 28 and 52.
2. **Prior exposure to schistosomiasis** will be evaluated by ELISA for IgG to schistosome egg antigen using stored blood samples collected at baseline.

For peer review only - http://bmjopen.bmj.com/site/about/guidelines.xhtml

3. **The presence of other helminth infections** will be determined retrospectively using stool PCR of samples collected at baseline and at weeks 28 and 52. In accord with national guidelines, all participants will be treated with albendazole or mebendazole after collection of samples for primary endpoints at week 8 and 28, and after collection of samples for secondary endpoints at week 52.
4. **Malarial fever:** Individuals presenting with fever will be investigated using rapid diagnostic tests for malaria and treated based on the results and according to prevailing national guidelines.
5. **Prior malaria exposure** will be evaluated by ELISA for IgG to malaria antigen using stored samples collected at baseline.

Sample size considerations

Based on the literature[6 32 33] and preliminary data, we anticipate that standard deviations (SDs) of primary outcome measures will lie between 0.3 and 0.6 log_{10}; that responses in rural, high-parasite settings may be 0.3-0.4 log_{10} smaller than in the urban setting, and that effective treatment of parasitic infections may restore responses by approximately 0.2 log_{10} (Tweyongyere *et al*.[34]). We therefore power our study to detect differences of this magnitude (0.2 log_{10}) or (in some cases) smaller. We assume malaria prevalence of $\geq$60% based on PCR.[18 19]

Based on these assumptions, we plan to include 640 participants in total (320 DP, 320 placebo); of whom 384 are expected to be malaria infected, giving 192 participants in each trial arm who are infected at baseline.

Table 2 shows power estimates, for 5% significance level and assuming 20% loss to follow-up.

Table 2. Power estimates (5% significance level)

	**Log_{10} difference**						
**Standard deviation (log_{10})**	**0.08**	**0.10**	**0.12**	**0.14**	**0.16**	**0.18**	**0.20**
192 DP vs 192 placebo (malaria infected only)							
0.3	65%	83%	94%	98%	>99%	>99%	>99%
0.4	42%	59%	75%	87%	94%	98%	99%
0.5	29%	42%	56%	69%	80%	88%	94%
0.6	21%	31%	42%	53%	65%	75%	83%
Cells highlighted in grey correspond to >80% power.							

Ethical and regulatory considerations

Ethical approval has been granted from the Research Ethics Committees of the Uganda Virus Research Institute (UVRI REC, reference: GC/127/19/05/681) and the London School of Hygiene and Tropical Medicine (LSHTM, reference: 16033), and from the Uganda National Council for Science and Technology (UNCST, reference: HS 2487) and the Uganda National Drug Authority (NDA, reference: CTC0117/2020). Any protocol

For peer review only

For peer review only - http://bmjopen.bmj.com/site/about/guidelines.xhtml

amendments will be submitted to ethics committees and regulatory bodies for approval before implementation.

Participants will be adolescents and therefore a vulnerable human population. Care will be taken to provide adequate, age and education-status appropriate information and to ensure that it is understood; and to emphasise that participation is voluntary. Participants will be enrolled only when they have given their own assent and when consent has been given by the parent or guardian. No major risks to the participants are anticipated since all the treatments and vaccines to be given are licensed and known to be safe. The main risk to participants will be time lost from school work: we will work with teachers and parents to minimise disruption to classes, and will avoid enrolment of primary 7 students since these classes are involved in national examinations. Further risks are discussed in Supplementary information.

For this trial we gave particular consideration to the differential provision of malaria treatment. Monthly treatment with DP has been shown to reduce the prevalence of anaemia and reduce episodes of clinical malaria in Ugandan schools[26] but has not been adopted as standard of care. To manage the expected differential benefits of the interventions for anaemia, a full blood count will be performed at baseline, as discussed above; anaemic children will be managed appropriately and severely anaemic children excluded. Additional safety monitoring during the trial will be done by the Data Safety and Monitoring Board (DSMB). Further information on the DSMB can be found in Supplementary information.

Malaria infection status will be determined retrospectively through assays conducted in bulk on stored samples (malaria PCR). These results will not, therefore, be useful to determine management of individual participants. Participants in the placebo arms will receive lower levels of anti-malaria treatment. However, all trial arms will receive a minimum of well-implemented national standard of care. Malaria standard of care will comprise provision of bed nets to minimise malaria exposure for all participants. Rapid diagnostic tests and treatment will be made readily available for participants who develop symptomatic malaria.

Participant and public involvement

Concepts involved in this work have been discussed with colleagues at the Vector Control Division and Expanded Programme on Immunisation in the Ministry of Health (Uganda). Before commencing the trial, discussions will be held with officials at Jinja District Council, community leaders and Village Health Teams from sub-counties in which selected schools are located. In addition, we will engage teachers and parents in planning the detailed standard operating procedures for the study. Prior to recruitment we will hold meetings to explain the proposed work to teachers, parents and students and to address their questions. At the end of the study, results will be shared with these stakeholders and with participants.

Dissemination

For peer review only - http://bmjopen.bmj.com/site/about/guidelines.xhtml

292 Study findings will be published through open access peer-reviewed journals, presentations at local, national and international conferences and to the local community through community meetings. Anonymised participant level datasets generated will be available upon request.

295 ***Data management and analysis***

296 Socio-demographic information and clinical and laboratory measurements will be recorded and managed using REDCap (Research Electronic Data Capture) tools,[28 29] with paper-based forms as back-up. All data will be recorded under a unique study ID number. When paper forms must be used, data will be double entered in a study-specific database, with standard checks for discrepancies. All data for analysis will be anonymised and stored on a secure and password-protected server, with access limited to essential research personnel.

302 The effect of monthly intermittent preventive treatment (IPT) for malaria versus no IPT on the outcomes will be analysed. Information on malaria infection status will only be available after randomisation. The primary analysis will be done on individuals identified as infected at baseline (through the randomisation, these will be balanced between treatment arms); this will test the hypothesis that treating the infection (and subsequent reinfections) removes parasite-induced effects on vaccine responses. Secondary analyses will include all randomised individuals; this will provide insight into the potential benefit of the interventions as public health measures.

For peer review only

Discussion

Parasites have long been proposed as modulators of vaccine responses; malaria is highly prevalent in settings with poor vaccine responses. Drivers of population differences in vaccine response are not fully elucidated; improved understanding is important for effective vaccine development and implementation. If treating malaria improves vaccine responses, combining parasite-control with immunisation programmes offers an attractive, practical public health intervention for schools and communities.

This study aims to determine whether treating malaria with DP before and after immunisation with unrelated vaccines can improve vaccine responses in adolescents. The use of DP in this study is considered an attractive option for preventive treatment and preventive chemotherapy for malaria because of the long half-life of piperaquine (approximately 23 days).[35] Despite identified benefits in reducing episodes of malaria and anaemia,[26] DP has not been adopted as standard of care either in Uganda or elsewhere. This study is expected to add further evidence regarding the potential benefits of monthly DP for school children by determining the effect on vaccine responses, as well as collecting data on malaria infection status, thereby further contributing to policy debate in this field.

A concern arises where treatment of malaria with DP may have potential adverse effects such as cardiac toxicity, particularly dose-dependent prolongation of the QTc interval and associated increased risk of arrhythmias. There has been concern that provision of multiple doses might increase this risk. However, a recent meta-analysis involving 11 trials of DP (9 IPT, 2 treatment trials) involving 14,628 participants, 3,935 of whom received multiple treatments with DP, found no evidence to suggest significant cardiac toxicity.[35] Few of the studies measured electrocardiographic changes, but those that did found no increase in QTc intervals with increasing numbers of doses. Therefore, the use of DP in this study where malaria prevalence is high is not expected to pose a significant risk to the participants.

We expect about 60% of the adolescents to have malaria infection at the outset in the proposed study area. This may vary by season and year, and the prevalence could be much lower depending on the timing of the start of the study. If this becomes the case, the two study arms will not be different enough to test our hypothesis. To mitigate this, we will work closely with colleagues monitoring trends in malaria prevalence in Uganda and adjust our study site to optimise the likelihood of achieving the expected prevalence.

This interventional study will help us to understand how malaria affects responses to commonly used vaccines, which will inform implementation of public health programmes for immunisation and infection control especially in low- and middle-income countries.

Study timeline

Applications for ethical approval were submitted in May 2018, with approval received in September 2018 (Uganda Virus Research Institute Research Ethics Committee), May 2019 (Uganda National Council for Science and Technology), June 2019 (London School of Hygiene and Tropical Medicine) and January 2020

For peer review only - http://bmjopen.bmj.com/site/about/guidelines.xhtml

(National Drug Authority). Collaborator/investigator/trial steering committee meetings were also held during the initial 12-month planning period. Recruitment to POPVAC B is scheduled to commence in February 2021. Intervention will be up to 12 months, with completion of the project scheduled for April 2022.

Competing interests

Alison Elliott reports a grant from the Medical research Council, UK (POPVAC programme funding). The rest of the authors declare that they have no conflicts of interest.

Author contributions

AME conceived the study. AME, AN, GN, ELW, SC, LZ and SS contributed to study design. LZ, GO, PNK, SA, CO, RA, EN, and FA are site clinicians/nurses/clinical laboratory technicians providing valuable input on clinical considerations of the intervention. SK, FK, DN, MS and RK are field workers handling the organisational integration of the intervention. AN, AM and ELW are involved in organisation of the databases, trial randomization, treatment allocation and drawing up of analytical plans. LZ, GN, JN, AN, SC, ELW and AME drafted the manuscript. All authors reviewed the manuscript, contributed to it and approved the final version.

Acknowledgements

We thank the Uganda National Expanded Programme for Immunisation, Sanofi Pasteur and PaxVax for providing the HPV, yellow fever and oral typhoid vaccines, respectively. The BCG and tetanus-diphtheria vaccines were kind donations from the Serum Institute of India. We thank the Jinja district local government for their support. We also thank members of the POPVAC programme steering committee (chaired by Prof. Richard Hayes) and the Data and Safety Monitoring Board (Dr David Meya, Prof Andrew Prendergast and Dr Elizabeth George).

Funding

The POPVAC programme of work is supported by the Medical Research Council of the United Kingdom (grant number MR/R02118X/1). SC and JN are supported in part by the Makerere University – Uganda Virus Research Institute Centre of Excellence for Infection and Immunity Research and Training (MUII-plus). MUII-plus is funded under the DELTAS Africa Initiative. The DELTAS Africa Initiative is an independent funding scheme of the African Academy of Sciences (AAS), Alliance for Accelerating Excellence in Science in Africa (AESA) and supported by the New Partnership for Africa's Development Planning and Coordinating Agency (NEPAD Agency) with funding from the Wellcome Trust (grant 107743) and the UK Government. The MRC/UVRI and LSHTM Uganda Research Unit is jointly funded by the UK Medical Research Council (MRC)

For peer review only

For peer review only - http://bmjopen.bmj.com/site/about/guidelines.xhtml

and the UK Department for International Development (DFID) under the MRC/DFID Concordat agreement and is also part of the EDCTP2 programme supported by the European Union.

The study sponsor (London School of Hygiene and Tropical Medicine) and funders had no role in study design; collection, management, analysis, and interpretation of data; writing of the protocol; and the decision to submit the protocol for publication.

POPVAC trial team

Principal investigator*:* Alison Elliott; ***Project leader***: Ludoviko Zirimenya; ***laboratory staff****:* Gyaviira Nkurunungi, Stephen Cose, Rebecca Amongin, Beatrice Nassanga, Jacent Nassuuna, Irene Nambuya, Prossy Kabuubi, Emmanuel Niwagaba, Gloria Oduru, Grace Kabami; ***statisticians and data managers****:* Emily Webb, Agnes Natukunda, Helen Akurut, Alex Mutebe; ***clinicians****:* Anne Wajja, Milly Namutebi, Christopher Zziwa; Joel Serubanja; ***nurses****:* Caroline Onen, Esther Nakazibwe, Josephine Tumusiime, Caroline Ninsiima, Susan Amongi, Florence Akello; ***internal monitor****:* Mirriam Akello; ***field workers****:* Robert Kizindo, Moses Sewankambo, Denis Nsubuga, Samuel Kiwanuka, Fred Kiwudhu; ***boatman****:* David Abiriga; ***administrative management****:* Moses Kizza, Samsi Nansukusa; ***internal and external collaborators:*** Pontiano Kaleebu, Hermelijn Smits, Maria Yazdanbakhsh, Govert van Dam, Paul Corstjens, Sarah Staedke, Henry Luzze, James Kaweesa, Edridah Tukahebwa, Elly Tumushabe, Moses Muwanga.

For peer review only

REFERENCES

1. Barreto ML, Pereira SM, Pilger D, et al. Evidence of an effect of BCG revaccination on incidence of tuberculosis in school-aged children in Brazil: second report of the BCG-REVAC cluster-randomised trial. *Vaccine* 2011;29(31):4875-7. doi: 10.1016/j.vaccine.2011.05.023

2. Barreto ML, Pilger D, Pereira SM, et al. Causes of variation in BCG vaccine efficacy: examining evidence from the BCG REVAC cluster randomized trial to explore the masking and the blocking hypotheses. *Vaccine* 2014;32(30):3759-64. doi: 10.1016/j.vaccine.2014.05.042

3. Black GF, Weir RE, Floyd S, et al. BCG-induced increase in interferon-gamma response to mycobacterial antigens and efficacy of BCG vaccination in Malawi and the UK: two randomised controlled studies. *Lancet* 2002;359(9315):1393-401. doi: 10.1016/S0140-6736(02)08353-8

4. Fine PE. Variation in protection by BCG: implications of and for heterologous immunity. *Lancet* 1995;346(8986):1339-45.

5. Taniuchi M, Platts-Mills JA, Begum S, et al. Impact of enterovirus and other enteric pathogens on oral polio and rotavirus vaccine performance in Bangladeshi infants. *Vaccine* 2016;34(27):3068-75. doi: 10.1016/j.vaccine.2016.04.080 [published Online First: 2016/05/08]

6. Muyanja E, Ssemaganda A, Ngauv P, et al. Immune activation alters cellular and humoral responses to yellow fever 17D vaccine. *The Journal of clinical investigation* 2014;124(7):3147-58. doi: 10.1172/jci75429 [published Online First: 2014/06/10]

7. van Riet E, Adegnika AA, Retra K, et al. Cellular and humoral responses to influenza in gabonese children living in rural and semi-urban areas. *The Journal of infectious diseases* 2007;196(11):1671-8. doi: 10.1086/522010 [published Online First: 2007/11/17]

8. van Riet E, Retra K, Adegnika AA, et al. Cellular and humoral responses to tetanus vaccination in Gabonese children. *Vaccine* 2008;26(29-30):3690-5. doi: 10.1016/j.vaccine.2008.04.067 [published Online First: 2008/06/10]

9. Tanner R, Kakalacheva K, Miller E, et al. Serum indoleamine 2,3-dioxygenase activity is associated with reduced immunogenicity following vaccination with MVA85A. *BMC infectious diseases* 2014;14:660. doi: 10.1186/s12879-014-0660-7 [published Online First: 2014/12/04]

10. Sissoko MS, Healy SA, Katile A, et al. Safety and efficacy of PfSPZ Vaccine against Plasmodium falciparum via direct venous inoculation in healthy malaria-exposed adults in Mali: a randomised, double-blind phase 1 trial. *The Lancet Infectious diseases* 2017;17(5):498-509. doi: 10.1016/s1473-3099(17)30104-4 [published Online First: 2017/02/22]

11. Venkatraman N, Ndiaye BP, Bowyer G, et al. Safety and Immunogenicity of a Heterologous Prime-Boost Ebola Virus Vaccine Regimen in Healthy Adults in the United Kingdom and Senegal. *The Journal of infectious diseases* 2019;219(8):1187-97. doi: 10.1093/infdis/jiy639

12. Rodrigues LC, Noel Gill O, Smith PG. BCG vaccination in the first year of life protects children of Indian subcontinent ethnic origin against tuberculosis in England. *Journal of epidemiology and community health* 1991;45(1):78-80. doi: 10.1136/jech.45.1.78 [published Online First: 1991/03/01]

13. Flaherty DK, Vesosky B, Beamer GL, et al. Exposure to Mycobacterium avium can modulate established immunity against Mycobacterium tuberculosis infection generated by Mycobacterium bovis BCG vaccination. *Journal of leukocyte biology* 2006;80(6):1262-71. doi: 10.1189/jlb.0606407 [published Online First: 2006/09/14]

14. Brandt L, Feino Cunha J, Weinreich Olsen A, et al. Failure of the Mycobacterium bovis BCG vaccine: some species of environmental mycobacteria block multiplication of BCG and induction of protective immunity to tuberculosis. *Infection and immunity* 2002;70(2):672-8. [published Online First: 2002/01/18]

15. Bentwich Z, Kalinkovich A, Weisman Z, et al. Can eradication of helminthic infections change the face of AIDS and tuberculosis? *Immunol Today* 1999;20(11):485-7.

For peer review only

For peer review only - http://bmjopen.bmj.com/site/about/guidelines.xhtml

16. Sanya RE, Nkurunungi G, Andia Biraro I, et al. A life without worms. *Transactions of the Royal Society of Tropical Medicine and Hygiene* 2017:1-9. doi: 10.1093/trstmh/trx010 [published Online First: 2017/03/25]

17. Geneva: World Health Organization. World Malaria Report 2019; https://www.who.int/publications-detail/world-malaria-report-2019.

18. Rehman AM, Maiteki-Sebuguzi C, Gonahasa S, et al. Intermittent preventive treatment of malaria delivered to primary schoolchildren provided effective individual protection in Jinja, Uganda: secondary outcomes of a cluster-randomized trial (START-IPT). *Malar J* 2019;18(1):318. doi: 10.1186/s12936-019-2954-0 [published Online First: 2019/09/20]

19. Staedke SG, Maiteki-Sebuguzi C, Rehman AM, et al. Assessment of community-level effects of intermittent preventive treatment for malaria in schoolchildren in Jinja, Uganda (START-IPT trial): a cluster-randomised trial. *The Lancet Global Health* 2018;6(6):e668-e79. doi: https://doi.org/10.1016/S2214-109X(18)30126-8

20. Cunnington AJ, Riley EM. Suppression of vaccine responses by malaria: insignificant or overlooked? *Expert review of vaccines* 2010;9(4):409-29. doi: 10.1586/erv.10.16 [published Online First: 2010/04/08]

21. Crawley J, Sismanidis C, Goodman T, et al. Effect of intermittent preventive treatment for malaria during infancy on serological responses to measles and other vaccines used in the Expanded Programme on Immunization: results from five randomised controlled trials. *Lancet* 2012;380(9846):1001-10. doi: 10.1016/s0140-6736(12)60775-2 [published Online First: 2012/08/02]

22. Brown J, Baisley K, Kavishe B, et al. Impact of malaria and helminth infections on immunogenicity of the human papillomavirus-16/18 AS04-adjuvanted vaccine in Tanzania. *Vaccine* 2014;32(5):611-7. doi: 10.1016/j.vaccine.2013.11.061

23. Usen S, Milligan P, Ethevenaux C, et al. Effect of fever on the serum antibody response of Gambian children to Haemophilus influenzae type b conjugate vaccine. *The Pediatric infectious disease journal* 2000;19(5):444-9. [published Online First: 2000/05/20]

24. Obeng-Adjei N, Portugal S, Tran TM, et al. Circulating Th1-Cell-type Tfh Cells that Exhibit Impaired B Cell Help Are Preferentially Activated during Acute Malaria in Children. *Cell reports* 2015;13(2):425-39. doi: 10.1016/j.celrep.2015.09.004 [published Online First: 2015/10/07]

25. Chan AW, Tetzlaff JM, Altman DG, et al. SPIRIT 2013 statement: defining standard protocol items for clinical trials. *Ann Intern Med* 2013;158(3):200-7. doi: 10.7326/0003-4819-158-3-201302050-00583 [published Online First: 2013/01/09]

26. Nankabirwa JI, Wandera B, Amuge P, et al. Impact of intermittent preventive treatment with dihydroartemisinin-piperaquine on malaria in Ugandan schoolchildren: a randomized, placebo-controlled trial. *Clinical infectious diseases : an official publication of the Infectious Diseases Society of America* 2014;58(10):1404-12. doi: 10.1093/cid/ciu150 [published Online First: 2014/03/14]

27. World Health Organization. Guidelines for the treatment of malaria – 3rd edition., 2015.

28. Harris PA, Taylor R, Minor BL, et al. The REDCap Consortium: Building an International Community of Software Platform Partners. *Journal of biomedical informatics* 2019:103208. doi: 10.1016/j.jbi.2019.103208 [published Online First: 2019/05/13]

29. Harris PA, Taylor R, Thielke R, et al. Research electronic data capture (REDCap)--a metadata-driven methodology and workflow process for providing translational research informatics support. *Journal of biomedical informatics* 2009;42(2):377-81. doi: 10.1016/j.jbi.2008.08.010 [published Online First: 2008/10/22]

30. Plotkin SA. Correlates of protection induced by vaccination. *Clinical and vaccine immunology : CVI* 2010;17(7):1055-65. doi: 10.1128/cvi.00131-10 [published Online First: 2010/05/14]

For peer review only

For peer review only - http://bmjopen.bmj.com/site/about/guidelines.xhtml

31. Corstjens PL, Nyakundi RK, de Dood CJ, et al. Improved sensitivity of the urine CAA lateral-flow assay for diagnosing active Schistosoma infections by using larger sample volumes. *Parasites & vectors* 2015;8:241. doi: 10.1186/s13071-015-0857-7 [published Online First: 2015/04/22]

32. Fletcher HA, Snowden MA, Landry B, et al. T-cell activation is an immune correlate of risk in BCG vaccinated infants. *Nat Commun* 2016;7:11290. doi: 10.1038/ncomms11290

33. Safaeian M, Porras C, Pan Y, et al. Durable antibody responses following one dose of the bivalent human papillomavirus L1 virus-like particle vaccine in the Costa Rica Vaccine Trial. *Cancer prevention research (Philadelphia, Pa)* 2013;6(11):1242-50. doi: 10.1158/1940-6207.capr-13-0203 [published Online First: 2013/11/06]

34. Tweyongyere R, Nassanga BR, Muhwezi A, et al. Effect of Schistosoma mansoni infection and its treatment on antibody responses to measles catch-up immunisation in pre-school children: A randomised trial. *PLoS neglected tropical diseases* 2019;13(2):e0007157. doi: 10.1371/journal.pntd.0007157 [published Online First: 2019/02/15]

35. Gutman J, Kovacs S, Dorsey G, et al. Safety, tolerability, and efficacy of repeated doses of dihydroartemisinin-piperaquine for prevention and treatment of malaria: a systematic review and meta-analysis. *The Lancet Infectious diseases* 2017;17(2):184-93. doi: 10.1016/s1473-3099(16)30378-4 [published Online First: 2016/11/21]

FIGURE LEGENDS

Figure 1. Outline of immunisations and interventions

[1]Primary endpoints will be at eight weeks post BCG and four weeks post Yellow fever (YF-17D), oral typhoid (Ty21a), Human Papilloma Virus (HPV) and Tetanus/diptheria (Td) vaccination.

[2]Primary endpoint for responses to Td given at 28 weeks.

For peer review only

For peer review only - http://bmjopen.bmj.com/site/about/guidelines.xhtml

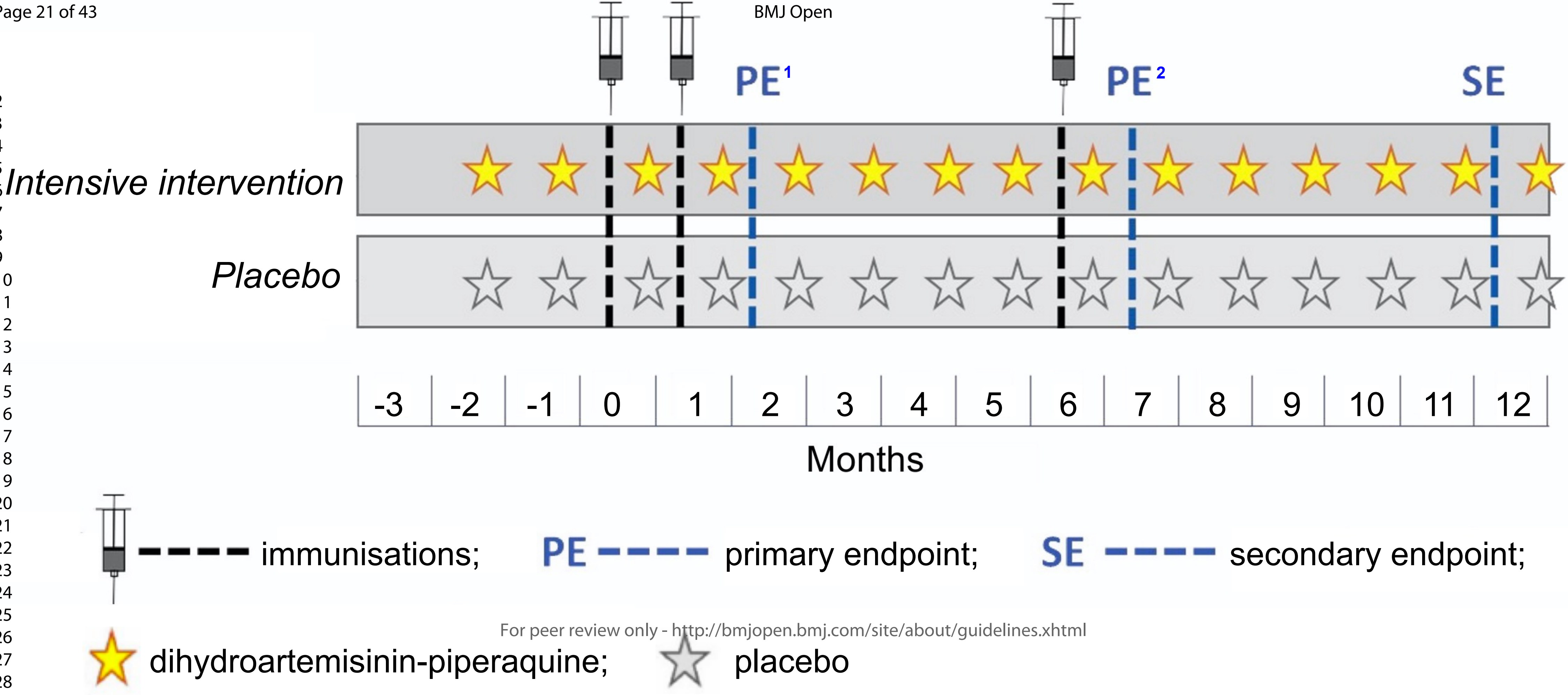

For peer review only - http://bmjopen.bmj.com/site/about/guidelines.xhtml

SUPPLEMENTARY INFORMATION

The effect of intermittent preventive treatment for malaria with dihydroartemisinin-piperaquine on immune responses to vaccines among rural Ugandan adolescents: randomised controlled trial protocol B for the 'POPulation differences in VACcine responses' (POPVAC) programme

Agnes Natukunda[1,¶], Ludoviko Zirimenya[1,¶], Gyaviira Nkurunungi[1,¶,*], Jacent Nassuuna[1], Gloria Oduru[1], Rebecca Amongin[1], Prossy N Kabuubi[1], Alex Mutebe[1], Caroline Onen[1], Susan Amongi[1], Esther Nakazibwe[1], Florence Akello[1], Samuel Kiwanuka[1], Fred Kiwudhu[1], Moses Sewankambo[1], Denis Nsubuga[1], Robert Kizindo[1], Sarah G Staedke[2,3], Stephen Cose[1,3], Emily L Webb[4], Alison M Elliott[1,3] **for the POPVAC trial team**

[1]Immunomodulation and Vaccines Programme, Medical Research Council/Uganda Virus Research Institute and London School of Hygiene and Tropical Medicine (MRC/UVRI and LSHTM) Uganda Research Unit, Entebbe, Uganda

[2]Infectious Diseases Research Collaboration, Kampala, Uganda

[3]Department of Clinical Research, London School of Hygiene and Tropical Medicine, London, United Kingdom

[4]MRC Tropical Epidemiology Group, Department of Infectious Disease Epidemiology, London School of Hygiene and Tropical Medicine, London, United Kingdom

[¶]These authors contributed equally

***Correspondence:** Gyaviira Nkurunungi; gyaviira.nkurunungi@lshtm.ac.uk

For peer review only

21 SUPPLEMENTARY INFORMATION

Table S1. POPVAC B: schedule of visits and procedures

VISIT NUMBER	1	2, 3	4	5	6[9]	6.2	7	8	9, 10, 11, 12, 13	14	15	16	17,18, 19, 20, 21	22
WEEKS FROM 1ST IMMUNISATION	-8[1]	-6[10], -2	0	2	4	4 + 4 days	6	8	10, 14, 18, 22 , 26	28	30	32	34, 38, 42, 46, 50	52
	Screening	Treatment (Rx only)	Immunisation	Treatment (Rx only)	Immunisations		Rx only	Primary endpoint (PE)	Rx only	Immunisations	Rx only	PE[2]	Rx only	Secondary endpoint (SE)
**RANDOMISED DIHYDROARTEMISININ-PIPERAQUINE INTERVENTION**														
DP treatment arm (x)		x		x			x		x		x		x	
DP control arm (o represents placebo)		o		o			o		o		o		o	
Albendazole								x[3]				x[3]		x[3]
Praziquantel								x[3,6]						x[3,6]
**VACCINES**														
BCG			x											
YF-17D					x									
Ty21a					x									
HPV					x			[x][4]		x				
Td										x				[x][5]
**INVESTIGATIONS/PROCEDURES**														
Inclusion/exclusion criteria	x													
Informed consent	x													
Questionnaire	x		x		x	x		x		x		x		x
Examination	x		(x)		(x)	(x)		(x)		(x)		(x)		(x)
Urine β-HCG test (female only) 1mL	x		x		x					x				
Urine YF viral load						x								
Stool for PCR and storage	x									x				x
Stool for coproantibody and storage	x							x						
**BLOOD SAMPLES**														
Malaria PCR (1ml)	x		x		x			x		x		x		x
Serology for HIV, prior malaria and *S. mansoni* (0.5ml)	x													
Mansonella perstans (1ml)	x													
Serum/plasma CAA (1ml)	x									x				x
Hb[8]/Full blood count (0.5ml)	x		x		x					x				
Assessments of pre-immunisation responses, and/or vaccine response outcomes and/or exploratory immunology; storage[7] (10-20ml)			x		x			x		x		x		x
Blood for gene expression (2ml)			x		x					x				
Blood vol (mL)	4		27		17			14-24		27		14		14

Cumulative blood vol (mL)[7]	4		31		48			72		99		113		127

PE: primary endpoint; **SE:** secondary endpoint; **Rx only**: treatment only
Immunisation days highlighted in green, primary end point days in red, days for treatment only in grey
(**x**) performed if clinically indicated

1. Screening and enrolment into Project B will take place at least 8 weeks before immunisation 0 to allow initiation of the DP intervention.
2. Week 32 is primary endpoint for responses to Td given at 28 weeks; secondary endpoint for HPV booster
3. Treatments given after sampling when schedules coincide
4. Week 8 HPV dose will be given for previously-unvaccinated girls aged ≥14 years
5. Week 52 Td booster dose will be provided as a service
6. Praziquantel if required in accordance with Ministry of Health policy for the selected schools
7. Exploratory immunology blood volume will be guided by guidelines from Harvard Mass General, where a maximum of 3ml/kg body weight is taken at any one time point and not more than 3ml/kg is taken over any 8 week period (ref http://www.drgreene.com/21_1616.html.) These guidelines have been followed in a previous study vaccinating adolescents with investigational tuberculosis vaccine MVA85A (in Uganda).[1] The total blood volume planned is 72 ml over the initial intensive sampling period of 8 weeks. Revision of sample volumes based on weight will only be required for participants who weigh less than 24 kg; the average weight of children aged 9 years is expected to be 28kg (with 21kg the 3rd centile) with greater weights for older children.[2]
8. At Baseline, it will only be Hb estimation by Haemocue
9. Oral typhoid vaccine doses will be administered on four alternate days namely visit 6, 6.1, 6.2 and 6.3.
10. The first DP and Placebo administration at week -6 will be administered at the end of the screening visit

For peer review only

Further information on the recruitment criteria

- Participants who are excluded from the trial because they have been discovered during screening procedures to be suffering from a previously undiagnosed condition thought to require further medical attention will be referred appropriately for further investigation and treatment.
- Participants discovered to have severe anaemia will be excluded from the trial and treated for anaemia and any evident underlying cause.
- Participants discovered to be HIV-positive will be counselled and offered a CD4 T-cell count and referred to a provider for further care and antiretroviral treatment (since this is indicated for all HIV-positive children under national guidelines).
- Participants discovered to be pregnant will be counselled and referred to an antenatal clinic of their choice.

This trial proposes to recruit all participants within a short time-frame. It will not, therefore, be possible to reconsider enrolment of potential participants with temporary exclusion criteria after treatment and resolution of the condition.

Further rationale for the selection of vaccines

Bacillus Calmette-Guérin (BCG)

BCG is a live, replicating parenteral vaccine, the only licensed vaccine against TB. BCG vaccine for these studies will be obtained from the Serum Institute of India either directly, or through a supplier in Uganda. The Serum Institute of India provides much of the BCG vaccine used in Uganda.

Worldwide, TB is among the top 10 causes of death; Uganda has an estimated incidence of 202/100,000 people.[3] Infectious, sputum positive, pulmonary TB classically emerges in adolescence, driving the on-going epidemic.[4] Thus adolescent booster immunisation is a key TB control strategy.[5] However, as discussed above, BCG vaccine response and efficacy are often impaired in tropical and rural settings[6-8] and new TB vaccines are similarly affected.[9] In the past, WHO has been hesitant to recommend BCG re-vaccination. However, in 2017 WHO's Strategic Advisory Group of Experts (SAGE) recommended, "Further research is warranted to explore whether certain sub-groups of age, geographic or *M. tuberculosis* exposure categories would benefit from re-vaccination."[10] Recent results suggest that, despite the variability of BCG efficacy between populations, BCG vaccination in adolescence offers benefit in some tropical settings, especially for individuals who are not yet infected with *Mycobacterium tuberculosis*, and may also be cost-effective.[7 11] Also, BCG vaccine is currently being used among adolescents in South Africa as a comparator in a trial of a novel TB vaccine (trial registration NCT02075203). To our knowledge, BCG efficacy in Ugandan adolescents, and differences in BCG vaccine responses between urban and rural Ugandan populations, have not been tested. Information obtained from this study is expected to further inform the use of BCG in adolescents, and also to inform the development of new vaccines for tuberculosis.

For peer review only

Yellow fever vaccine

Yellow fever vaccine YF-17D is a live replicating parenteral vaccine. The vaccine (Stamaril; Sanofi Pasteur) is available for purchase in Uganda. Yellow Fever (YF) causes outbreaks in Uganda and the wider region[12] and YF-17D is a candidate for Uganda's expanded programme on immunisation. Lower vaccine replication, lower neutralising antibody induction, and greater waning, are described in Uganda compared to Switzerland.[13] YF-17D is a potential vector for novel vaccine constructs,[14] adding relevance to vaccine development.

Typhoid vaccine Ty21a

Typhoid vaccine Ty21a is a live replicating oral vaccine and also a potential vector for new vaccine constructs.[15] Ty21a vaccine will be purchased from PaxVax, Redwood City, California. Substantial, multi-year typhoid outbreaks occur in Uganda and immunisation campaigns have been advocated as cost effective.[16] Schistosomiasis has been associated with prolonged S. typhi infection[17] and impaired antibody responses to killed typhoid vaccines.[18]

Ty21a was developed in the 1970s. Although not routinely used in Uganda, it has been (and is currently) registered in many countries. It was first registered in the United States and United Kingdom in the 1980s, and is recommended by the World Health Organisation for both endemic and epidemic settings.[19] It has comparable efficacy to the parenteral Vi polysaccharide typhoid vaccine, good durability and minimal adverse effects.[19] It is proposed for use in this study to model effects of study exposures and intervention on the response to a live oral vaccine.

The Ty21a vaccine is given as a three-dose regimen on alternate days.

Human Papilloma Virus (HPV) vaccine

Human Papilloma Virus (HPV) vaccine is a protein virus-like particle. The quadrivalent HPV Vaccine Gardasil (Merck) is available for purchase in Uganda and is the vaccine used by the national EPI programme. Studies after three vaccine doses have found somewhat enhanced responses in the presence of malaria, but no effect of helminths.[20] No study has previously investigated parasite effects on the priming response, but recent results for tetanus suggest that priming may be more susceptible than boosting to adverse effects.[21] This will be important if forthcoming trials support single-dose HPV immunisation (NCT02834637). HPV immunisation is being rolled out among girls to prevent cervical neoplasia, the commonest cancer among Ugandan women and we will coordinate provision with the national HPV immunisation programme.[22] HPV immunisation is also beneficial for boys since HPV infection is associated with anogenital warts, anal cancer and oropharyngeal cancers in both males and females, and with penile cancer in men,[23] and we will include boys in these studies.

For peer review only

Tetanus and diphtheria vaccines

Tetanus and diphtheria vaccines comprise inert toxoids (Td). Booster immunisation is recommended for young women to prevent maternal and neonatal tetanus. Recent evidence emphasises the need to protect young men also.[24] Uganda's EPI programme recommends tetanus boosters in adolescence and plans to change from tetanus alone to Td in 2018.

Additional considerations regarding the vaccine schedule

Live vaccines given in combination may influence the response to each other – a phenomenon described as "interference". Observations in the 1960s suggested that elevated circulating interferon (IFN)-γ after measles immunisation might interfere with the response to Vaccinia[25] and, to avoid such interference between live vaccines, it was recommended that live vaccines be given either together or three to four weeks apart.[26] However, with the introduction of new, live vaccines into use, Public Health England reviewed and revised this recommendation in 2014, limiting it to vaccines for which there was an evidence base (**Table S2**). We have adopted a four-week interval between BCG immunisation and the other proposed live vaccines (YF and Ty21a), which will be given together.[27]

Non-live vaccines can be given at the same time as live vaccines and there are no specific recommendations as to the number of non-live vaccines that can be given together. Two injections can be given into the same muscle although it is suggested that these should be at least 2.5 cm apart in case it is necessary to distinguish local adverse reactions to the two injections.[28] Our schedule avoids giving more than two injections on the same date but at week 4 it may be appropriate to give two into the deltoid muscle of the same arm if a BCG scar is developing on the other arm. Nevertheless, uncertainties remain regarding the effects of vaccines on responses to each other. Generalisations from this programme of work will need to take potential "interference" between vaccines into account.

For peer review only

Table S2: Public Health England recommendations for giving more than one live attenuated vaccine in current use in the UK[29]

Vaccine combinations	Recommendations
Yellow Fever and Measles, Mumps, Rubella (MMR)	A four week minimum interval period should be observed between the administration of these two vaccines. Yellow Fever and MMR should not be administered on the same day.
Varicella (and zoster) vaccine and MMR	If these vaccines are not administered on the same day, then a four week minimum interval should be observed between vaccines.
Tuberculin skin testing (Mantoux) and MMR	If a tuberculin skin test has already been initiated, then MMR should be delayed until the skin test has been read unless protection against measles is required urgently. If a child has had a recent MMR, and requires a tuberculin test, then a four week interval should be observed.
All currently used live vaccines (BCG, rotavirus, live attenuated influenza vaccine (LAIV), oral typhoid vaccine, yellow fever, varicella, zoster and MMR) and tuberculin (Mantoux) skin testing.	Apart from those combinations listed above, these live vaccines can be administered at any time before or after each other. This includes tuberculin (Mantoux) skin testing.

Immunisation Postponement Criteria

If any one of the following is identified at the time scheduled for immunisation, the participant may be immunised at a later date, or withdrawn, at the discretion of the Investigator. The participant must be followed until resolution of the event as with any adverse event:

- Acute disease at the time of immunisation. Acute disease is defined as the presence of a moderate or severe illness with or without fever. All vaccines can be administered to persons with a minor illness such as diarrhoea or mild upper respiratory infection with or without low-grade fever, i.e. temperature of ≤37.5°C (99.5°F)
- Temperature of >37.5°C (99.5°F) at the time of immunisation
- Taking antibiotics or antimalarials currently, or within the past 7 days, of the date of Ty21a administration (ascertained verbally)

Vaccine storage and transport

In order to maintain a reliable vaccine cold chain, the vaccines and diluents to be used will be stored and transported within the recommended temperature range of +2°C to +8°C. Care will be taken to ensure that the vaccines are not frozen. BCG, being sensitive to light, will be kept in the dark (normally within its secondary packaging) for as long as possible to protect it during storage and transportation. All vaccines will be kept in appropriate refrigeration equipment with a temperature monitoring device to ensure temperatures remain between +2°C and +8°C. Cold boxes/vaccines carriers with temperature monitors will be used to transport vaccines and the diluents from the MRC/UVRI and LSHTM Uganda Research Unit (Entebbe) to Jinja and while transporting vaccines to immunisation sessions. Designated staff will be given responsibility for managing the vaccine cold chain. All cold chain equipment including the temperature monitoring devices used for this project will comply with relevant technical specifications as defined by the EPI standards. Basic routine maintenance will be regularly carried out on all cold chain equipment.

Additional laboratory measurements

Additional assays will comprise HIV serology, pregnancy testing and full blood counts. HIV testing and pregnancy testing will be accompanied by appropriate counselling by trained staff.

HIV serology will be done on blood samples using rapid tests and according to prevailing national algorithms.[30] This will be done at baseline.

- Pregnancy testing will be done using urine samples and standard operating procedures for assessment of urine β-human chorionic gonadotropin (βhCG). This will be done at baseline and before immunisation on each immunisation day.
- Full blood counts will be conducted using a haematology analyser. Mild, moderate and severe anaemia will be defined according to WHO guidelines, by age.[31] This will be done at baseline to test

For peer review only

for anaemia as part of the eligibility assessment, and pre-immunisation as part of the assessment of immunological profile.

Individuals found to be HIV positive or pregnant will be referred to appropriate providers for further care. Individuals with severe anaemia (haemoglobin <82g/L) will be excluded from the randomised intervention (since the intervention might be beneficial in management of anaemia). They will be treated for anaemia and for any underlying cause identified.

Sample handling and archive

Blood and other samples will be processed according to local laboratory standard operating procedures (SOPs). All samples will reach the laboratory in anonymised form.

A sample archive will be developed. Although our current programme of work will address specific hypotheses regarding pathways of effects of parasites and interventions, the sample archive will provide a major asset for exploration of new leads arising from this work, or for an alternative, "systems biology" approach employing (for example) proteomic, genomic, epigenetic and transcriptomic analyses, and investigating the microbiome and virome. Information provided to participants, and consent forms, will include considerations of sample storage, and the possibility of sample analysis in laboratories within and outside Uganda. Participants will be able to decide if they will permit such future use of any leftover samples. We plan to store the samples for up to 20 years. If further storage is needed after that time, permission will be requested from the Uganda Virus Research Institute and London School of Hygiene and Tropical Medicine ethical review committees. If they elect not to permit this, all of those leftover samples will be discarded after the completion of the work included in the current protocol.

Operational considerations

Programme governance

A Programme Steering Committee has been set up to guide progress across all projects. This comprises the following:

- An independent chair
- Representatives from the Ministry of Health programmes for immunisation and for vector borne disease control
- Representatives of district authorities (Mukono and Jinja districts)
- Community representatives
- Principal investigator and co-investigators
- Project leader and post-doctoral immunologist
- Trial statistician
- Laboratory manager

For peer review only

- Medical Research Council observer

Informed consent

Both written informed assent from the participants and written informed consent from a parent or guardian will be required for participation, although these may not necessarily be obtained at the same time. Information will be provided in both English and the appropriate local language. For individuals who cannot speak the languages used, or who cannot read or write, a witness who can read the information sheet and translate the information to the participant or parent/guardian will be used. Two different types of age specific assent forms will be used for the group of participants aged 9 – 12 years and for the group aged 13 – 17 years. Informed consent by emancipated or mature minors will be obtained using a designated consent form for these categories of participants

The aims of the study, all tests, treatments and immunisations to be carried out and potential risks will be explained. The participant will be given the opportunity to ask about details of the trial, and will then have time to consider whether or not to participate. If they do decide to participate, they and their parent/guardian will sign and date two copies of the assent and consent forms, one for them to take away and keep, and one to be stored securely by the research team. Separate information and consent forms will be provided (i) for consent for storage of samples for future studies and for anonymous sharing of data from this study and (ii) for possible genetic studies; the information sheet will explain that these data may be used in analyses related to this protocol.

Screening and Eligibility Assessment

Once the informed consent process has been completed, and consent (and assent) given, a baseline medical history (including concomitant medication) will be collected. Vital signs will be checked and a physical examination will be performed. Inclusion and exclusion criteria will be checked.

Participants will undergo pre- and post-test counselling for HIV and (for girls) pregnancy testing by a trained and experienced nurse- or clinician-counsellor. Blood, urine and stool samples will be obtained, for tests as specified in the schedule of procedures. These tests are to exclude the major, immunomodulating co-infection, HIV, and conditions that might impact safety (anaemia, pregnancy).

Enrolment

Participants who consent/assent, complete the screening processes, satisfy all the inclusion criteria and meet none of the exclusion criteria will be enrolled.

Discontinuation / withdrawal criteria

In accordance with the principles of the current revision of the Declaration of Helsinki and any other applicable regulations, a participant has the right to withdraw from the study at any time and for any reason, and is not obliged to give his or her reasons for doing so. The Investigator may withdraw the

For peer review only

participant at any time in the interests of the participant's health and well-being. In addition, the participant may withdraw/be withdrawn for any of the following reasons:

- Ineligibility (either arising during the study or retrospectively, having been overlooked at screening)
- Administrative decision by the Investigator
- Significant protocol deviation
- Participant non-compliance with study requirements
- An adverse event which requires discontinuation of the study involvement or results in inability to continue to comply with study procedures.

Any participant who becomes pregnant during the trial will be followed up until the end of the pregnancy but no further immunisations will be given unless indicated during pregnancy (as is the case for tetanus toxoid). The trial allocation for this participant will be unblinded and the participant will only be given further treatment if clinically indicated. The babies will also be followed up and examined for any adverse effects. We will not routinely perform venepuncture in a pregnant participant.

The reason for withdrawal will be recorded in the case report form (CRF). If withdrawal is due to an AE, appropriate follow-up visits or medical care will be arranged, with the agreement of the participant, until the AE has resolved, stabilised or a non-trial related causality has been assigned.

If a participant withdraws from the study samples collected before their withdrawal from the trial will be used/ stored unless the participant specifically requests otherwise.

Trial discontinuation

The trial will be discontinued in the event of new scientific information that renders continuation futile or unethical, or for any other reason, at the discretion of the Programme Steering Committee.

End of study definition

The trial will be completed when the last participant enrolled into the trial has completed their final follow up visit.

Safety assessments and oversight

No new investigational drug or product will be used in the proposed trial. However, standard approaches for monitoring safety and reporting of serious adverse events will be followed.

Monitoring

The trial will be monitored by both internal and external monitors according to a pre-defined monitoring plan which will include a site initiation visit, monitoring visits at least annually, and a close-out visit. The monitors will assess patient safety, data integrity, and adherence to the protocol and to Good Clinical Research Practice procedures.

For peer review only

Considerations regarding standard of care

Malaria infection status will be determined retrospectively through assays conducted in bulk on stored samples (malaria PCR). These results will not, therefore, be useful to determine management of individual participants.

Participants in the placebo arms will receive lower levels of anti-malaria treatment. However, all trial arms will receive a minimum of well-implemented national standard of care.

Dihydroartemisinin/piperaquine is considered an attractive option for preventive treatment and preventive chemotherapy for malaria because of the long half-life of piperaquine (approximately 23 days).[32] Monthly treatment with DP has been shown to reduce the prevalence of anaemia and reduce episodes of clinical malaria in Ugandan schools[33] but has not been adopted as standard of care. This programme is expected to add further evidence regarding the potential benefits of monthly DP for school children by determining the effect on vaccine responses, thereby further contributing to policy debate in this field. To manage the expected differential benefits of the interventions for anaemia, a full blood count will be performed at baseline, as discussed above; anaemic children will be managed appropriately and severely anaemic children excluded.

Malaria standard of care will comprise provision of bed nets to minimise malaria exposure for all participants. Rapid diagnostic tests and treatment will be made readily available for participants who develop symptomatic malaria.

Albendazole will be provided twice a year to manage nematode infections (after collection of primary and secondary endpoint samples).

Procedures to be followed in the event of abnormal findings

Abnormal clinical findings from medical history, examination or blood tests will be assessed as to their clinical significance throughout the trials. If an abnormal test result is deemed clinically significant, it may be repeated. If a test remains clinically significant, the participant will be informed and appropriate medical care arranged as appropriate and with the permission of the participant. Specific details regarding findings, discussion with participants and resulting actions will be recorded in the clinical records. Decisions to exclude the participant from enrolling in the trial or to withdraw a participant from the trial will be at the discretion of the Investigator.

Data and Safety Monitoring Board (DSMB)

A data and safety monitoring board (DSMB) will be appointed to provide real-time safety oversight. The DSMB will be notified within 7 days of the Investigators' being aware of the occurrence of SAEs. The DSMB may recommend the Investigators to place the trial on hold if deemed necessary following an intervention-related SAE. The DSMB will be chaired by a clinician experienced in clinical trials. There will be a minimum

For peer review only

of two other appropriately qualified committee members. In the case of events related to a blinded intervention, the DSMB can request unblinding. Membership will include a statistician, and at least one Ugandan member. All correspondence between Investigators and the DSMB will be conveyed by the Principal Investigator to the trial Sponsor. The Chair of the DSMB will be contacted for advice and independent review by the Investigator or trial Sponsor in the following situations:

- The occurrence of any SAE
- Any other situation where the Investigator or trial Sponsor feels independent advice or review is important

Ethical and regulatory considerations

Further information regarding risks

The immunisations to be given have recognised side effects which are usually mild and resolve spontaneously in a few days to one week. Parenteral vaccines are likely to result in pain and swelling at the site of injection and mild fever; very occasionally pain and swelling can be severe and associated with difficulty in moving the shoulder. Sometimes headache and tiredness occur. Rarely a vaccine may cause a severe allergic reaction. For most vaccines this is estimated at less than one in a million doses (but 1 in 55,000 for Yellow Fever vaccine).[34] Individuals with a history of a possible allergic reaction to drugs or vaccines, or to vaccine components including eggs or chicken proteins, will be excluded from the studies. The research team will be trained and prepared to manage severe allergic reactions.

Adverse reactions to Yellow Fever vaccine include severe nervous system reaction (about 1 person in 125,000) and severe, life-threatening illness with organ failure (about 1 person in 250,000). The mortality for this severe, life-threatening adverse effect is reported as about 50%.[34]

BCG immunisation is likely to induce a scar in many cases. This may develop over several weeks, starting as a small papule at the injection site which may become ulcerated and then heal over a period of 2 to 5 months; and lymphadenopathy may develop. Occasionally a more severe local reaction occurs (estimated at 1 per 1,000-10,000 doses): for example, an abscess develops and scars may develop into keloids. Rarely BCG can cause disseminated disease (1 per 230,000 to 640,000 doses), or disease in sites remote from the immunisation site. Disseminated BCG disease usually occurs in immunocompromised people: HIV positive people will be excluded from these studies.[35] BCG "pre-immunisation" may interfere with the response to the subsequent live vaccines; indeed our hypothesis, and published results, suggest that it may suppress replication of YF 17D vaccine.[36] However, this reduced replication has not been shown to correlate with, or result in, reduced levels of neutralising antibody titres (which are the desired protective outcome).[13 36]

Oral typhoid vaccine (Ty21a) may occasionally be associated with stomach pain, nausea, vomiting and (rarely) rash.[34]

For peer review only

310 Dihydroartemisinin/piperaquine is generally well tolerated and has been used widely in practice, as well as in a substantial number of trials.[32] The main potential adverse effect of concern is cardiac toxicity, particularly dose-dependent prolongation of the QTc interval and associated increased risk of arrhythmias. There has been concern that provision of multiple doses might increase this risk. However, a recent meta-analysis involving 11 trials of DP (9 IPT, 2 treatment trials) involving 14,628 participants, 3,935 of whom received multiple treatments with DP found no evidence to suggest significant cardiac toxicity.[32] Few of the studies measured electrocardiographic changes, but those that did so found no increase in QTc intervals with increasing numbers of doses.

Benefits

All the treatments and vaccines to be given are licensed and regarded as safe. In general, the vaccines and treatments are expected to provide protection against infectious diseases.

With regard to BCG immunisation or revaccination in adolescence, benefits with respect to protection against tuberculosis among Ugandan adolescents are unknown and may, at best, be modest. There may be non-specific benefits. WHO's SAGE committee concluded, in their summary of October 2017,[37] that "BCG revaccination is safe in *Mycobacterium tuberculosis* infected and uninfected populations. There is a lack of evidence from randomized controlled trials and retrospective cohort and case-control studies demonstrating the efficacy and effectiveness of BCG revaccination in adolescents and adults after primary BCG vaccination in infancy for protection against TB disease. Due to absence of evidence, BCG revaccination is not considered cost-effective. Further research is warranted to explore whether certain sub-groups of age, geographic or M. tuberculosis exposure categories would benefit from revaccination." We hope, through this work, to contribute to this debate.

Participants and their families, schools and communities are expected to benefit from improved understanding of malaria, and of vaccines.

For peer review only

REFERENCES

1. Wajja A, Kizito D, Nassanga B, et al. The effect of current Schistosoma mansoni infection on the immunogenicity of a candidate TB vaccine, MVA85A, in BCG-vaccinated adolescents: An open-label trial. *PLoS neglected tropical diseases* 2017;11(5):e0005440. doi: 10.1371/journal.pntd.0005440 [published Online First: 2017/05/05]

2. WHO. Growth reference 5-19 years. 2007

3. WHO. Global tuberculosis report 2016 http://www.who.int/tb/publications/global_report/en/ (accessed 17 June 2017), 2016.

4. Alcais A, Fieschi C, Abel L, et al. Tuberculosis in children and adults: two distinct genetic diseases. *J Exp Med* 2005;202(12):1617-21. doi: 10.1084/jem.20052302

5. Weiner J, 3rd, Kaufmann SH. Recent advances towards tuberculosis control: vaccines and biomarkers. *J Intern Med* 2014;275(5):467-80. doi: 10.1111/joim.12212

6. Fine PE. Variation in protection by BCG: implications of and for heterologous immunity. *Lancet* 1995;346(8986):1339-45.

7. Barreto ML, Pilger D, Pereira SM, et al. Causes of variation in BCG vaccine efficacy: examining evidence from the BCG REVAC cluster randomized trial to explore the masking and the blocking hypotheses. *Vaccine* 2014;32(30):3759-64. doi: 10.1016/j.vaccine.2014.05.042

8. Black GF, Weir RE, Floyd S, et al. BCG-induced increase in interferon-gamma response to mycobacterial antigens and efficacy of BCG vaccination in Malawi and the UK: two randomised controlled studies. *Lancet* 2002;359(9315):1393-401. doi: 10.1016/S0140-6736(02)08353-8

9. Tanner R, Kakalacheva K, Miller E, et al. Serum indoleamine 2,3-dioxygenase activity is associated with reduced immunogenicity following vaccination with MVA85A. *BMC infectious diseases* 2014;14:660. doi: 10.1186/s12879-014-0660-7 [published Online First: 2014/12/04]

10. WHO. SAGE Evidence to recommendations framework. 2017. http://www.who.int/immunization/sage/meetings/2017/october/2_EvidencetoRecommendationFramework_BCG.pdf (accessed 16th March 2018).

11. Dye C. Making wider use of the world's most widely used vaccine: Bacille Calmette-Guerin revaccination reconsidered. *Journal of the Royal Society, Interface* 2013;10(87):20130365. doi: 10.1098/rsif.2013.0365 [published Online First: 2013/08/02]

12. Kraemer MU, Faria NR, Reiner RC, Jr., et al. Spread of yellow fever virus outbreak in Angola and the Democratic Republic of the Congo 2015-16: a modelling study. *The Lancet Infectious diseases* 2016 doi: 10.1016/s1473-3099(16)30513-8 [published Online First: 2016/12/27]

13. Muyanja E, Ssemaganda A, Ngauv P, et al. Immune activation alters cellular and humoral responses to yellow fever 17D vaccine. *The Journal of clinical investigation* 2014;124(7):3147-58. doi: 10.1172/jci75429 [published Online First: 2014/06/10]

14. Aguiar M, Stollenwerk N, Halstead SB. The Impact of the Newly Licensed Dengue Vaccine in Endemic Countries. *PLoS neglected tropical diseases* 2016;10(12):e0005179. doi: 10.1371/journal.pntd.0005179 [published Online First: 2016/12/22]

15. Dharmasena MN, Osorio M, Filipova S, et al. Stable expression of Shigella dysenteriae serotype 1 O-antigen genes integrated into the chromosome of live Salmonella oral vaccine vector Ty21a. *Pathogens and disease* 2016 doi: 10.1093/femspd/ftw098 [published Online First: 2016/09/23]

For peer review only

16. Carias C, Walters MS, Wefula E, et al. Economic evaluation of typhoid vaccination in a prolonged typhoid outbreak setting: the case of Kasese district in Uganda. *Vaccine* 2015;33(17):2079-85. doi: 10.1016/j.vaccine.2015.02.027 [published Online First: 2015/02/26]

17. Melhem RF, LoVerde PT. Mechanism of interaction of Salmonella and Schistosoma species. *Infection and immunity* 1984;44(2):274-81. [published Online First: 1984/05/01]

18. Muniz-Junqueira MI, Tavares-Neto J, Prata A, et al. Antibody response to Salmonella typhi in human schistosomiasis mansoni. *Revista da Sociedade Brasileira de Medicina Tropical* 1996;29(5):441-5. [published Online First: 1996/09/01]

19. WHO. Position Paper on Typhoid vaccines: WHO position paper – March 2018 2018

20. Brown J, Baisley K, Kavishe B, et al. Impact of malaria and helminth infections on immunogenicity of the human papillomavirus-16/18 AS04-adjuvanted vaccine in Tanzania. *Vaccine* 2014;32(5):611-7. doi: 10.1016/j.vaccine.2013.11.061

21. Riner DK, Ndombi EM, Carter JM, et al. Schistosoma mansoni Infection Can Jeopardize the Duration of Protective Levels of Antibody Responses to Immunizations against Hepatitis B and Tetanus Toxoid. *PLoS neglected tropical diseases* 2016;10(12):e0005180. doi: 10.1371/journal.pntd.0005180

22. Centre HI. HPV and related diseases report: Uganda. 2016. http://www.hpvcentre.net/statistics/reports/UGA.pdf (accessed 20.01.2017).

23. WHO. Human papillomavirus vaccines: WHO position paper, May 2017. *Releve epidemiologique hebdomadaire* 2017;92(19):241-68. [published Online First: 2017/05/23]

24. Nanteza B, Galukande M, Aceng J, et al. The burden of tetanus in Uganda. *SpringerPlus* 2016;5(1):705. doi: 10.1186/s40064-016-2309-z [published Online First: 2016/06/29]

25. Petralli JK, Merigan TC, Wilbur JR. ACTION OF ENDOGENOUS INTERFERON AGAINST VACCINIA INFECTION IN CHILDREN. *Lancet* 1965;2(7409):401-5. [published Online First: 1965/08/28]

26. C-A S. Vaccine Immunology. In: Plotkin SA, Orenstein W, Offit P, et al., eds. Vaccines: Elsevier 2017.

27. PHE. Public Health England: revised recommendations for the administration of more than one

live vaccine 2015

28. PHE. Immunization procedures. The Green Book. Public Health England. 2012

29. PHE. Public Health England: revised recommendations for the administration of more than one live vaccine 2015

30. Ministry of Health–Uganda. Uganda Clinical Guidelines 2016. http://apps.who.int/medicinedocs/documents/s23532en/s23532en.pdf

31. WHO. Haemoglobin concentrations for the diagnosis of anaemia and assessment of severity. Vitamin and Mineral Nutrition Information System. Geneva, World Health Organization, 2011 (WHO/NMH/NHD/MNM/11.1).

32. Gutman J, Kovacs S, Dorsey G, et al. Safety, tolerability, and efficacy of repeated doses of dihydroartemisinin-piperaquine for prevention and treatment of malaria: a systematic review and meta-analysis. *The Lancet Infectious diseases* 2017;17(2):184-93. doi: 10.1016/s1473-3099(16)30378-4 [published Online First: 2016/11/21]

33. Nankabirwa JI, Wandera B, Amuge P, et al. Impact of intermittent preventive treatment with dihydroartemisinin-piperaquine on malaria in Ugandan schoolchildren: a randomized, placebo-controlled trial. *Clinical infectious diseases : an official publication of the Infectious Diseases Society of America* 2014;58(10):1404-12. doi: 10.1093/cid/ciu150 [published Online First: 2014/03/14]

34. CDC. Centers for Disease Control and Prevention, vaccines and immunizations.

35. WHO. Information sheet observed rate of vaccine reactions Bacille Calmette-Guérin (BCG) vaccine. 2012

36. Arts RJW, Moorlag S, Novakovic B, et al. BCG Vaccination Protects against Experimental Viral Infection in Humans through the Induction of Cytokines Associated with Trained Immunity. *Cell host & microbe* 2018;23(1):89-100.e5. doi: 10.1016/j.chom.2017.12.010 [published Online First: 2018/01/13]

37. WHO. Immunization, vaccines and biologicals. Vaccine position papers. 2017

For peer review only

Reporting checklist for protocol of a clinical trial.

Based on the SPIRIT guidelines.

Instructions to authors

Complete this checklist by entering the page numbers from your manuscript where readers will find each of the items listed below.

Your article may not currently address all the items on the checklist. Please modify your text to include the missing information. If you are certain that an item does not apply, please write "n/a" and provide a short explanation.

Upload your completed checklist as an extra file when you submit to a journal.

In your methods section, say that you used the SPIRITreporting guidelines, and cite them as:

Chan A-W, Tetzlaff JM, Altman DG, Laupacis A, Gøtzsche PC, Krleža-Jerić K, Hróbjartsson A, Mann H, Dickersin K, Berlin J, Doré C, Parulekar W, Summerskill W, Groves T, Schulz K, Sox H, Rockhold FW, Rennie D, Moher D. SPIRIT 2013 Statement: Defining standard protocol items for clinical trials. Ann Intern Med. 2013;158(3):200-207

		Reporting Item	Page Number
**Administrative information**			
Title	#1	Descriptive title identifying the study design, population, interventions, and, if applicable, trial acronym	1
Trial registration	#2a	Trial identifier and registry name. If not yet registered, name of intended registry	2
Trial registration: data set	#2b	All items from the World Health Organization Trial Registration Data Set	n/a
Protocol version	#3	Date and version identifier	Information available at ISRCTN62041885.
Funding	#4	Sources and types of financial, material, and other support	14

For peer review only

Roles and responsibilities: contributorship	#5a	Names, affiliations, and roles of protocol contributors	14, 15
Roles and responsibilities: sponsor contact information	#5b	Name and contact information for the trial sponsor	Information available at ISRCTN62041885.
Roles and responsibilities: sponsor and funder	#5c	Role of study sponsor and funders, if any, in study design; collection, management, analysis, and interpretation of data; writing of the report; and the decision to submit the report for publication, including whether they will have ultimate authority over any of these activities	15
Roles and responsibilities: committees	#5d	Composition, roles, and responsibilities of the coordinating centre, steering committee, endpoint adjudication committee, data management team, and other individuals or groups overseeing the trial, if applicable (see Item 21a for data monitoring committee)	Information detailed in supplementary information file
**Introduction**			
Background and rationale	#6a	Description of research question and justification for undertaking the trial, including summary of relevant studies (published and unpublished) examining benefits and harms for each intervention	4
Background and rationale: choice of comparators	#6b	Explanation for choice of comparators	7
Objectives	#7	Specific objectives or hypotheses	5
Trial design	#8	Description of trial design including type of trial (eg, parallel group, crossover, factorial, single group), allocation ratio, and framework (eg, superiority, equivalence, non-inferiority, exploratory)	6

For peer review only

**Methods: Participants, interventions, and outcomes**			
Study setting	#9	Description of study settings (eg, community clinic, academic hospital) and list of countries where data will be collected. Reference to where list of study sites can be obtained	6
Eligibility criteria	#10	Inclusion and exclusion criteria for participants. If applicable, eligibility criteria for study centres and individuals who will perform the interventions (eg, surgeons, psychotherapists)	6
Interventions: description	#11a	Interventions for each group with sufficient detail to allow replication, including how and when they will be administered	7
Interventions: modifications	#11b	Criteria for discontinuing or modifying allocated interventions for a given trial participant (eg, drug dose change in response to harms, participant request, or improving / worsening disease)	Detailed in supplementary information file
Interventions: adherance	#11c	Strategies to improve adherence to intervention protocols, and any procedures for monitoring adherence (eg, drug tablet return; laboratory tests)	Detailed in supplementary information file
Interventions: concomitant care	#11d	Relevant concomitant care and interventions that are permitted or prohibited during the trial	Detailed in supplementary information file
Outcomes	#12	Primary, secondary, and other outcomes, including the specific measurement variable (eg, systolic blood pressure), analysis metric (eg, change from baseline, final value, time to event), method of aggregation (eg, median, proportion), and time point for each outcome. Explanation of the clinical relevance of chosen	9

For peer review only

		efficacy and harm outcomes is strongly recommended	
Participant timeline	#13	Time schedule of enrolment, interventions (including any run-ins and washouts), assessments, and visits for participants. A schematic diagram is highly recommended (see Figure)	Detailed in supplementary information file, Table S1
Sample size	#14	Estimated number of participants needed to achieve study objectives and how it was determined, including clinical and statistical assumptions supporting any sample size calculations	10
Recruitment	#15	Strategies for achieving adequate participant enrolment to reach target sample size	11
**Methods: Assignment of interventions (for controlled trials)**			
Allocation: sequence generation	#16a	Method of generating the allocation sequence (eg, computer-generated random numbers), and list of any factors for stratification. To reduce predictability of a random sequence, details of any planned restriction (eg, blocking) should be provided in a separate document that is unavailable to those who enrol participants or assign interventions	7
Allocation concealment mechanism	#16b	Mechanism of implementing the allocation sequence (eg, central telephone; sequentially numbered, opaque, sealed envelopes), describing any steps to conceal the sequence until interventions are assigned	7
Allocation: implementation	#16c	Who will generate the allocation sequence, who will enrol participants, and who will assign participants to interventions	7

For peer review only

Blinding (masking)	#17a	Who will be blinded after assignment to interventions (eg, trial participants, care providers, outcome assessors, data analysts), and how	8
Blinding (masking): emergency unblinding	#17b	If blinded, circumstances under which unblinding is permissible, and procedure for revealing a participant's allocated intervention during the trial	Detailed in supplementary information file
**Methods: Data collection, management, and analysis**			
Data collection plan	#18a	Plans for assessment and collection of outcome, baseline, and other trial data, including any related processes to promote data quality (eg, duplicate measurements, training of assessors) and a description of study instruments (eg, questionnaires, laboratory tests) along with their reliability and validity, if known. Reference to where data collection forms can be found, if not in the protocol	11
Data collection plan: retention	#18b	Plans to promote participant retention and complete follow-up, including list of any outcome data to be collected for participants who discontinue or deviate from intervention protocols	Details in supplementary information file
Data management	#19	Plans for data entry, coding, security, and storage, including any related processes to promote data quality (eg, double data entry; range checks for data values). Reference to where details of data management procedures can be found, if not in the protocol	Details in supplementary information file. These will also be detailed in a statistical analysis plan that will be uploaded to the online trial registration.
Statistics: outcomes	#20a	Statistical methods for analysing primary and secondary outcomes. Reference to where other	12

For peer review only

		details of the statistical analysis plan can be found, if not in the protocol	These will also be detailed in a statistical analysis plan that will be uploaded to the online trial registration.
Statistics: additional analyses	#20b	Methods for any additional analyses (eg, subgroup and adjusted analyses)	12.<br>These will also be detailed in a statistical analysis plan that will be uploaded to the online trial registration.
Statistics: analysis population and missing data	#20c	Definition of analysis population relating to protocol non-adherence (eg, as randomised analysis), and any statistical methods to handle missing data (eg, multiple imputation)	These will be detailed in a statistical analysis plan that will be uploaded to the online trial registration.
**Methods: Monitoring**			
Data monitoring: formal committee	#21a	Composition of data monitoring committee (DMC); summary of its role and reporting structure; statement of whether it is independent from the sponsor and competing interests; and reference to where further details about its charter can be found, if not in the protocol. Alternatively, an explanation of why a DMC is not needed	Detailed in supplementary information file
Data monitoring: interim analysis	#21b	Description of any interim analyses and stopping guidelines, including who will have access to these interim results and make the final decision to terminate the trial	Detailed in supplementary information file
Harms	#22	Plans for collecting, assessing, reporting, and managing solicited and spontaneously reported adverse events and other unintended effects of trial interventions or trial conduct	Detailed in supplementary information file
Auditing	#23	Frequency and procedures for auditing trial conduct, if any, and whether the process will be independent from investigators and the sponsor	Detailed in supplementary information file

For peer review only

**Ethics and dissemination**			
Research ethics approval	#24	Plans for seeking research ethics committee / institutional review board (REC / IRB) approval	13
Protocol amendments	#25	Plans for communicating important protocol modifications (eg, changes to eligibility criteria, outcomes, analyses) to relevant parties (eg, investigators, REC / IRBs, trial participants, trial registries, journals, regulators)	10, 11
Consent or assent	#26a	Who will obtain informed consent or assent from potential trial participants or authorised surrogates, and how (see Item 32)	10 and detailed in supplementary information file
Consent or assent: ancillary studies	#26b	Additional consent provisions for collection and use of participant data and biological specimens in ancillary studies, if applicable	Detailed in supplementary information file
Confidentiality	#27	How personal information about potential and enrolled participants will be collected, shared, and maintained in order to protect confidentiality before, during, and after the trial	11
Declaration of interests	#28	Financial and other competing interests for principal investigators for the overall trial and each study site	14
Data access	#29	Statement of who will have access to the final trial dataset, and disclosure of contractual agreements that limit such access for investigators	n/a
Ancillary and post trial care	#30	Provisions, if any, for ancillary and post-trial care, and for compensation to those who suffer harm from trial participation	Detailed in supplementary information file
Dissemination policy: trial results	#31a	Plans for investigators and sponsor to communicate trial results to participants,	2, 11, 12

For peer review only

		healthcare professionals, the public, and other relevant groups (eg, via publication, reporting in results databases, or other data sharing arrangements), including any publication restrictions	
Dissemination policy: authorship	#31b	Authorship eligibility guidelines and any intended use of professional writers	n/a
Dissemination policy: reproducible research	#31c	Plans, if any, for granting public access to the full protocol, participant-level dataset, and statistical code	12
**Appendices**			
Informed consent materials	#32	Model consent form and other related documentation given to participants and authorised surrogates	n/a
Biological specimens	#33	Plans for collection, laboratory evaluation, and storage of biological specimens for genetic or molecular analysis in the current trial and for future use in ancillary studies, if applicable	n/a

None The SPIRIT checklist is distributed under the terms of the Creative Commons Attribution License CC-BY-ND 3.0. This checklist can be completed online using https://www.goodreports.org/, a tool made by the EQUATOR Network in collaboration with Penelope.ai

For peer review only

BMJ Open

The effect of intermittent preventive treatment for malaria with dihydroartemisinin-piperaquine on immune responses to vaccines among rural Ugandan adolescents: randomised controlled trial protocol B for the 'POPulation differences in VACcine responses' (POPVAC) programme

Journal:	*BMJ Open*
Manuscript ID	bmjopen-2020-040427.R1
Article Type:	Protocol
Date Submitted by the Author:	18-Oct-2020
Complete List of Authors:	Natukunda , Agnes ; MRC/UVRI and LSHTM Uganda Research Unit, Immunomodulation and Vaccines Programme<br>Nkurunungi, Gyaviira; MRC/UVRI and LSHTM Uganda Research Unit, Immunomodulation and Vaccines Programme<br>Zirimenya , Ludoviko ; MRC/UVRI and LSHTM Uganda Research Unit, Immunomodulation and Vaccines Programme<br>Nassuuna , Jacent; MRC/UVRI and LSHTM Uganda Research Unit, Immunomodulation and Vaccines Programme<br>Oduru, Gloria; MRC/UVRI and LSHTM Uganda Research Unit, Immunomodulation and Vaccines Programme<br>Amongin , Rebecca ; MRC/UVRI and LSHTM Uganda Research Unit, Immunomodulation and Vaccines Programme<br>Kabuubi , Prossy ; MRC/UVRI and LSHTM Uganda Research Unit, Immunomodulation and Vaccines Programme<br>Mutebe , Alex ; MRC/UVRI and LSHTM Uganda Research Unit, Immunomodulation and Vaccines Programme<br>Onen, Caroline; MRC/UVRI and LSHTM Uganda Research Unit, Immunomodulation and Vaccines Programme<br>Amongi, Susan; MRC/UVRI and LSHTM Uganda Research Unit, Immunomodulation and Vaccines Programme<br>Nakazibwe, Esther; MRC/UVRI and LSHTM Uganda Research Unit, Immunomodulation and Vaccines Programme<br>Akello , Florence ; MRC/UVRI and LSHTM Uganda Research Unit, Immunomodulation and Vaccines Programme<br>Kiwanuka, Samuel; MRC/UVRI and LSHTM Uganda Research Unit, Immunomodulation and Vaccines Programme<br>Kiwudhu, Fred; MRC/UVRI and LSHTM Uganda Research Unit, Immunomodulation and Vaccines Programme<br>Sewankambo, Moses; MRC/UVRI and LSHTM Uganda Research Unit, Immunomodulation and Vaccines Programme<br>Nsubuga, Denis; MRC/UVRI and LSHTM Uganda Research Unit, Immunomodulation and Vaccines Programme<br>Kizindo , Robert ; MRC/UVRI and LSHTM Uganda Research Unit, Immunomodulation and Vaccines Programme<br>Staedke, Sarah; London School of Hygiene and Tropical Medicine Department of Clinical Research; Infectious Diseases Research Collaboration,

	Cose , Stephen ; MRC/UVRI and LSHTM Uganda Research Unit, Immunomodulation and Vaccines Programme; London School of Hygiene and Tropical Medicine Department of Clinical Research<br>Webb, Emily; London School of Hygiene & Tropical Medicine, MRC Tropical Epidemiology Group, Department of Infectious Disease Epidemiology<br>Elliott, Alison ; MRC/UVRI and LSHTM Uganda Research Unit, Immunomodulation and Vaccines Programme; London School of Hygiene and Tropical Medicine Department of Clinical Research
**Primary Subject Heading**:	Infectious diseases
Secondary Subject Heading:	Epidemiology, Immunology (including allergy), Infectious diseases, Public health
Keywords:	Infection control < INFECTIOUS DISEASES, PARASITOLOGY, Public health < INFECTIOUS DISEASES, Immunology < TROPICAL MEDICINE, Epidemiology < TROPICAL MEDICINE, Paediatric infectious disease & immunisation < PAEDIATRICS

SCHOLARONE™ Manuscripts

I, the Submitting Author has the right to grant and does grant on behalf of all authors of the Work (as defined in the below author licence), an exclusive licence and/or a non-exclusive licence for contributions from authors who are: i) UK Crown employees; ii) where BMJ has agreed a CC-BY licence shall apply, and/or iii) in accordance with the terms applicable for US Federal Government officers or employees acting as part of their official duties; on a worldwide, perpetual, irrevocable, royalty-free basis to BMJ Publishing Group Ltd ("BMJ") its licensees and where the relevant Journal is co-owned by BMJ to the co-owners of the Journal, to publish the Work in this journal and any other BMJ products and to exploit all rights, as set out in our licence.

The Submitting Author accepts and understands that any supply made under these terms is made by BMJ to the Submitting Author unless you are acting as an employee on behalf of your employer or a postgraduate student of an affiliated institution which is paying any applicable article publishing charge ("APC") for Open Access articles. Where the Submitting Author wishes to make the Work available on an Open Access basis (and intends to pay the relevant APC), the terms of reuse of such Open Access shall be governed by a Creative Commons licence – details of these licences and which Creative Commons licence will apply to this Work are set out in our licence referred to above.

Other than as permitted in any relevant BMJ Author's Self Archiving Policies, I confirm this Work has not been accepted for publication elsewhere, is not being considered for publication elsewhere and does not duplicate material already published. I confirm all authors consent to publication of this Work and authorise the granting of this licence.

For peer review only

The effect of intermittent preventive treatment for malaria with dihydroartemisinin-piperaquine on immune responses to vaccines among rural Ugandan adolescents: randomised controlled trial protocol B for the 'POPulation differences in VACcine responses' (POPVAC) programme

Agnes Natukunda[1,¶], Gyaviira Nkurunungi[1,¶,*], Ludoviko Zirimenya[1,¶], Jacent Nassuuna[1], Gloria Oduru[1], Rebecca Amongin[1], Prossy N Kabuubi[1], Alex Mutebe[1], Caroline Onen[1], Susan Amongi[1], Esther Nakazibwe[1], Florence Akello[1], Samuel Kiwanuka[1], Fred Kiwudhu[1], Moses Sewankambo[1], Denis Nsubuga[1], Robert Kizindo[1], Sarah G Staedke[2,3], Stephen Cose[1,3], Emily L Webb[4], Alison M Elliott[1,3] **for the POPVAC trial team**

[1]Immunomodulation and Vaccines Programme, Medical Research Council/Uganda Virus Research Institute and London School of Hygiene and Tropical Medicine (MRC/UVRI and LSHTM) Uganda Research Unit, Entebbe, Uganda

[2]Infectious Diseases Research Collaboration, Kampala, Uganda

[3]Department of Clinical Research, London School of Hygiene and Tropical Medicine, London, United Kingdom

[4]MRC Tropical Epidemiology Group, Department of Infectious Disease Epidemiology, London School of Hygiene and Tropical Medicine, London, United Kingdom

[¶]These authors contributed equally

***Correspondence:** Gyaviira Nkurunungi; Gyaviira.Nkurunungi@mrcuganda.org

For peer review only

For peer review only - http://bmjopen.bmj.com/site/about/guidelines.xhtml

Abstract

Introduction

Drivers of lower vaccine efficacy and impaired vaccine-specific immune responses in low-income versus high-income countries, and in rural, compared to urban settings, are not fully elucidated. Repeated exposure to and immunomodulation by parasite infections may be important. We focus on *Plasmodium falciparum* malaria, aiming to determine whether there are reversible effects of malaria infection on vaccine responses.

Methods and analysis

We have designed a randomised, double-blind, placebo-controlled, parallel group trial of intermittent preventive malaria treatment versus placebo, to determine effects on vaccine response outcomes among school-going adolescents (9 to 17 years) from malaria-endemic rural areas of Jinja district (Uganda). Vaccines to be studied comprise BCG vaccine on day 'zero'; yellow fever, oral typhoid and Human Papilloma Virus vaccines at week 4; and Tetanus/diphtheria booster vaccine at week 28. Participants in the intermittent preventive malaria treatment arm will receive dihydroartemisinin piperaquine (DP) dosed by weight, one month apart, prior to the first immunisation, followed by monthly treatment thereafter. We expect to enrol 640 adolescents.

Primary outcomes are BCG-specific IFN-γ ELISpot responses eight weeks after BCG immunisation and for other vaccines, antibody responses to key vaccine antigens at four weeks after immunisation. In secondary analyses, we will determine effects of monthly DP treatment (versus placebo) on correlates of protective immunity, on vaccine response waning, on whether there are differential effects on priming vs boosting immunisations, and on malaria infection prevalence. We will also conduct exploratory immunology assays among subsets of participants to further characterise effects of the intervention on vaccine responses.

Ethics and dissemination

Ethics approval has been obtained from relevant Ugandan and UK ethics committees. Results will be shared with Uganda Ministry of Health, relevant district councils, community leaders and study participants. Further dissemination will be done through conference proceedings and publications.

Trial registration

Current Controlled Trials identifier: ISRCTN62041885

Article summary

Strengths and limitations of this study

For peer review only - http://bmjopen.bmj.com/site/about/guidelines.xhtml

- This will be the first well-powered trial to evaluate the effect of repeated intermittent presumptive anti-malarial treatment on vaccine response outcomes in adolescents.
- Effects on both live-attenuated and inert vaccines will be studied.
- The use of an interventional research design will contribute strong evidence to determine whether malaria has causal and reversible effects on vaccine response outcomes, and our strong immunoepidemiological design and nested immunological studies will make it possible to address specific hypotheses regarding pathways of effects.
- The use of vaccines most of which are part of Uganda's national Expanded Programme on Immunisation (EPI), makes the findings from this study applicable and could inform the introduction of combined school health programmes for malaria control and vaccine provision.
- With the high prevalence of malaria in the proposed study area, participants in the placebo arm may notice more frequent episodes of malaria and this could lead to individual self-unblinding and self-treatment, compromising the design of the study.

Word count

3757

Keywords

Malaria; Vaccine; Dihydroartemisinin; Immunization

For peer review only

For peer review only - http://bmjopen.bmj.com/site/about/guidelines.xhtml

Introduction

Population differences in vaccine-specific immune responses and efficacy have been documented. In tropical low-income, rural settings, responses to both live[1-6] and non-live[7 8] vaccines are often impaired, compared to urban and high-income country settings. Studies have also shown that responses to candidate vaccines (such as those to tuberculosis,[9] malaria[10] and Ebola[11]) are lower in Africa than in Europe or America. Drivers of these population differences in vaccine response are incompletely understood. While genetic differences may play a role, they cannot fully explain this phenomenon: for example, migrant and native English populations have comparable BCG vaccine efficacy.[12] Previous exposure to the pathogen targeted by the vaccine, or to related organisms, may mask the benefit of the vaccine;[13 14] however, this mechanism cannot explain observations for vaccines against rare organisms such as Ebola.[11] The role of immunomodulating environmental factors has been discussed,[2] but evidence is inconclusive. Parasites, particularly, have long been proposed as modulators of vaccine responses,[15 16] but this has not been fully substantiated in well-powered trials aimed at evaluating reversibility of their effects in human populations.[17]

This study is one of three parallel trials whose designs and cross-cutting analyses are described separately in this journal (bmjopen-2020-040425, bmjopen-2020-040426 and bmjopen-2020-040430). The focus of this POPVAC B trial is on infection with malaria parasites. Malaria remains highly prevalent in Africa and Uganda is among the six countries with the highest prevalence of malaria parasites on the continent.[18] A recent nationally representative survey estimated parasite prevalence (based on microscopy) of 32% in 8-10 year olds with wide variability by region.[19] Emerging insecticide and drug resistance threatens gains towards control.[20] Interventions like intermittent preventive treatment (IPT) are recommended by the World Health Organisation (WHO) for use in preventing malaria in pregnant women, infants, and young children living in areas of seasonal malaria transmission.[20] In a recently concluded trial in Uganda, IPT in schoolchildren was shown to be beneficial in protecting individual children and reducing community transmission of malaria.[21 22]

Several studies have previously reported impaired meningococcal, tetanus and typhoid vaccine responses in children infected with malaria parasites.[23-26] Another study showed that children receiving malaria chemoprophylaxis had better responses to meningococcal vaccine than children not receiving malaria chemoprophylaxis.[27] In a Ugandan study, malaria infection in mothers during pregnancy, and in their infants, was associated with reduced antibody responses to measles immunisation in infancy.[28] We have previously observed that infant malaria infection is associated with reduction in BCG and tetanus vaccine-specific responses.[29] On the other hand, malaria infection could improve responses to some vaccines and a study by Brown et al reported increased responses to an HPV vaccine among individuals with malaria parasitaemia.[30] In a 2010 review, Cunnington and Riley showed that, despite long-standing interest, the

For peer review only - http://bmjopen.bmj.com/site/about/guidelines.xhtml

impact of malaria on vaccine responses was not adequately understood.[16] Although there is little evidence that malaria impairs responses to protein vaccines given in multiple doses,[16 31 32] they found consistent evidence of an adverse effect on the response to polysaccharide vaccines.[16]

Malaria may impact vaccine responses due to acute immunological changes (associated with fever)[33], or by longer term effects (for example on T follicular helper cell and B cell function).[34] The extent to which infection with malaria impacts immunological characteristics associated with vaccine responses may best be determined by intervention studies. This trial protocol B of the 'Population differences in Vaccine responses" programme has been designed to evaluate the effect of intermittent preventive treatment of malaria on vaccine responses. We summarise the protocol here. This study is one of three parallel trials whose designs and cross-cutting analyses are described separately in this issue. Understanding the predictors of vaccine responses, and the factors that drive them, will contribute to finding ways of improving vaccine efficacy for rural, tropical settings.

For peer review only

For peer review only - http://bmjopen.bmj.com/site/about/guidelines.xhtml

114 **Hypothesis**

115 The overarching goal of the POPVAC programme is to understand population differences in vaccine
116 responses in Uganda, in order to identify strategies through which vaccine effectiveness can be optimised
117 for the low-income, tropical settings where they are especially needed. For this Trial B, we focus on the
118 hypothesis *that malaria infection suppresses responses to unrelated vaccines, and that this effect can be*
119 *reversed, at least in part, by intermittent preventive treatment of malaria in schools in high transmission*
120 *settings.*

121 **Objective**

122 To determine whether there are reversible effects of malaria infection on vaccine response in adolescents,
123 using an intervention study.

For peer review only

For peer review only - http://bmjopen.bmj.com/site/about/guidelines.xhtml

Methods and analysis

Design, setting and participants

SPIRIT reporting guidelines[35] are used. We will conduct an individually randomised, double-blind, placebo-controlled, parallel group trial of monthly dihydroartemisinin/piperaquine (DP) versus placebo among primary school children in rural schools in Jinja district, Uganda. We will recruit 640 participants, randomising 320 to each trial arm. The study cohort will recruit participants aged 9 to 17 years from primary school year 1 up to 6 (to avoid primary leaving examinations in late year 7, and loss to follow up of children who leave from primary 7). Schools will be selected purposefully to assure malaria prevalence appropriate to our design. The study setting is remote from Lake Victoria and the River Nile, in order to minimise exposure to schistosomiasis.

Recruitment criteria

Inclusion criteria

i. Attending the selected school and planning to continue to attend the school for the duration of the study
ii. Aged 9 to 17 years and enrolled in primary 1 to 6
iii. Written informed assent by participant and consent by parent or guardian
iv. Agree to avoid pregnancy for the duration of the trial (female only)
v. Willing to provide locator information and to be contacted during the course of the trial
vi. Able and willing (in the investigator's opinion) to comply with all the study requirements

Exclusion criteria

i. Clinically significant history of immunodeficiency (including HIV), cancer, cardiovascular disease, gastrointestinal disease, liver disease, renal disease, endocrine disorder or neurological illness
ii. Moderate or severe acute illness characterised by any of the following symptoms: fever, impaired consciousness, convulsions, difficulty in breathing, vomiting; or as determined by the attending project clinician.
iii. Family history of sudden death attributable to heart condition in a first degree relative
iv. Family history of long QT syndrome
v. Know congenital prolongation of the corrected QT (QTc) interval
vi. History of known heart disease or fainting
vii. Known allergy or history of adverse reaction to DP or to artemether-lumefantrine
viii. History of serious psychiatric condition or disorder
ix. Previous immunisation with Yellow Fever (YF), oral typhoid or Human Papillomavirus (HPV) vaccine; previous immunisation with BCG or Tetanus and diphtheria vaccine (Td) at age $\geq$5 years

For peer review only

For peer review only - http://bmjopen.bmj.com/site/about/guidelines.xhtml

x. Concurrent oral or systemic steroid medication or the concurrent use of other immunosuppressive agents within 2 months prior to enrolment
xi. Current use of medications known to prolong the QT interval
xii. History of allergic reaction to immunisation or any allergy likely to be exacerbated by any component of the study vaccines including egg or chicken proteins
xiii. Tendency to develop keloid scars
xiv. Haemoglobin less than 80g/L
xv. Positive HIV serology
xvi. Positive pregnancy test
xvii. Female currently lactating, confirmed pregnancy or intention to become pregnant during the trial period
xviii. Use of an investigational medicinal product or non-registered drug, live vaccine, or medical device other than the study vaccines for 30 days prior to dosing with the study vaccine, or planned use during the study period
xix. Administration of immunoglobulins and/or any blood products within the three months preceding the planned trial immunisation date.

Further information on recruitment criteria can be found in Supplementary information.

Interventions

We will individually randomise participants in a 1:1 ratio to an experimental intervention of monthly dihydroartemisinin-piperaquine (DP) versus a DP-matching placebo in a double-blind, placebo-controlled trial (**Figure 1** and Supplementary **Table S1**). DP has been shown to reduce prevalence of asymptomatic malaria by 94% and malaria incidence by 96% among Ugandan school children.[36] The DP treatment arm will receive two doses, one month apart, prior to the first immunisation, followed by monthly treatment thereafter. Each dose will be calculated based on the participant's weight in kilograms.[37] DP (Dihydroartemisinin 40mg + Piperaquine Phosphate 320mg) tablets will be administered once a day for three consecutive days. The standard arm will receive placebo since, as yet, routine preventive malaria treatment in schools is not Uganda Ministry of Health policy.

Randomisation and allocation to treatment arm

An independent statistician will generate the randomisation code using a randomly permuted block size. This code will be embedded into a web-based randomisation system in REDCap (Research Electronic Data Capture) software.[38 39] At enrolment, eligibility criteria will be checked and eligible participants will be allocated sequentially to the next randomisation number, with the corresponding trial arm designated in REDCap. The randomisation code will be kept securely by the trial statistician with a second copy held by a

For peer review only - http://bmjopen.bmj.com/site/about/guidelines.xhtml

190 data manager or statistician not otherwise involved in the trial at the MRC/UVRI and LSHTM Uganda
191 Research Unit.

192 ***Blinding***

193 The trial will be double-blind, and will remain so until data collection and data cleaning are complete and
194 data locked for analysis, unless unblinding is required for individual participants who become pregnant (for
195 these cases unblinding can be requested by the Principal Investigator) or as a result of serious adverse
196 events or reactions (at the request of the Data and Safety Monitoring Board).

197 ***Immunisations***

198 We will study a portfolio of licensed vaccines (live and inert, oral and parental, priming and boosting)
199 expected to be beneficial (in some cases, already given) to adolescents in Uganda. Our schedule (**Table 1**
200 and **Table S1**) will comprise three main immunisation days (week 0, week 4 and week 28). Additional HPV
201 immunisation will be provided for girls aged 14 years or above, and a second Tetanus/diphtheria boost will
202 be given after completion of the study, to accord with the national Expanded Programme on Immunisation
203 (EPI) routines but the response to these will not specifically be addressed. Further rationale for the
204 selection of vaccines is detailed in Supplementary information. Our schedule has been developed in
205 consultation with the EPI programme and is cognisant of potential interference between vaccines
206 (Supplementary information).

207

Table 1. Immunisation schedule

	Immunisation week 0	Immunisation week 4	[Immunisation week 8]	Immunisation week 28	[Immunisation week 52]
**Live vaccines**	BCG vaccination / re-vaccination[1]	Yellow fever (YF-17D)<br>Oral typhoid (Ty21a)			
**Non-live vaccines**		HPV prime[2]	HPV boost for girls aged ≥14 years[3,4]	HPV boost[2] and Tetanus/ diphtheria (Td) boost	Tetanus/ diphtheria (Td) boost[4,5]

1. Prior BCG status may vary (data on history and documentation of prior BCG, and presence of a BCG scar, will be documented although these approaches have limitations for determining BCG status)
2. Both girls and boys will receive the HPV vaccine
3. The National EPI programme recommends three doses of HPV vaccine for older girls
4. These doses will be given to comply with guidelines but outcomes specifically relating to these doses will not be assessed
5. Priming by immunisation in infancy is assumed

208

209 ***Schedule of immunisation and sampling***

210 The schedule of immunisation and sampling is outlined in **Figure 1** and **Table S1**. While optimal timings for
211 outcome measures vary between vaccines, sampling at 8 weeks post BCG and 4 weeks post YF-17D, Ty21a,
212 HPV and Td is proposed for the primary endpoints, targeting the establishment of memory responses and
213 approximate peak of antibody responses.[40-44] A secondary endpoint at one year will assess waning. All

For peer review only - http://bmjopen.bmj.com/site/about/guidelines.xhtml

analyses will take baseline measurements into account. Immunisation postponement criteria are detailed in Supplementary information.

Outcomes

Primary outcomes

These will be assessed in all participants.

i. **BCG:** BCG-specific IFN-γ ELISpot response eight weeks post BCG immunisation.
ii. **YF-17D:** neutralising antibody titres (plaque-reduction neutralisation test) at four weeks post YF immunisation.
iii. **Ty21a:** *Salmonella typhi* lipopolysaccharide (LPS)-specific immunoglobulin (Ig)G concentration at four weeks post Ty21a immunisation.
iv. **HPV:** IgG specific for L1-proteins of HPV-16/18 at four weeks post HPV priming immunisation.
v. **Td:** Tetanus and diphtheria toxoid-specific IgG concentration at four weeks post Td immunisation.

Secondary outcomes

These will be assessed in all participants and will further investigate estimates of protective immunity (for vaccines where these are available) and dynamics of the vaccine responses, as well as the impact of the interventions on parasite clearance.

i. **Protective immunity.** Proportions with protective neutralising antibody (YF); protective IgG levels (TT);[45] seroconversion rates (Ty21a) at four weeks post the corresponding immunisation.
ii. **Response waning.** Primary outcome measures (all vaccines) repeated at week 52, and area-under-the curve (AUC) analyses.
iii. **Priming versus boosting.** Effects on priming versus boosting will be examined for HPV only, comparing outcomes four weeks after the first, and four weeks after the second vaccine dose.
iv. **Current malaria infection status and intensity** will be assessed retrospectively by PCR on stored samples collected on immunisation days and at week 52.

Furthermore, our sample collection will offer opportunities for an array of exploratory immunological evaluations on stored samples, focusing mainly on vaccine antigen specific outcomes. Exploratory assessments will provide further detail on immune response characteristics over the study time-course, and on the role of immunological profiles in malaria-mediated modulation of vaccine-specific responses.

Additional evaluation of parasite infection

1. **Current *S. mansoni* infection status and intensity** will be determined by serum/plasma levels of circulating anodic antigen (CAA). This method is quantitative, highly specific for *Schistosoma*

For peer review only

For peer review only - http://bmjopen.bmj.com/site/about/guidelines.xhtml

infection, and much more sensitive than the conventional Kato Katz method.[46] CAA will be assessed retrospectively on stored samples collected at baseline and at weeks 28 and 52.

2. **Prior exposure to schistosomiasis** will be evaluated by ELISA for IgG to schistosome egg antigen using stored blood samples collected at baseline.
3. **The presence of other helminth infections** will be determined retrospectively using stool PCR of samples collected at baseline and at weeks 28 and 52. In accord with national guidelines, all participants will be treated with albendazole or mebendazole after collection of samples for primary endpoints at week 8 and 28, and after collection of samples for secondary endpoints at week 52.
4. **Malarial fever:** Individuals presenting with fever will be investigated using rapid diagnostic tests for malaria and treated based on the results and according to prevailing national guidelines.
5. **Prior malaria exposure** will be evaluated by ELISA for IgG to malaria antigen using stored samples collected at baseline.

Sample size considerations

Based on the literature[6 47 48] and preliminary data, we anticipate that standard deviations (SDs) of primary outcome measures will lie between 0.3 and 0.6 log_{10}; that responses in rural, high-parasite settings may be 0.3-0.4 log_{10} smaller than in the urban setting, and that effective treatment of parasitic infections may restore responses by approximately 0.2 log_{10} (Tweyongyere *et al*.[49]). We therefore power our study to detect differences of this magnitude (0.2 log_{10}) or (in some cases) smaller. Among 5 to 15 year olds in this study setting, previous studies have reported malaria prevalence of >50% based on microscopy;[21 22] we assume malaria prevalence of $\geq$60% based on PCR.

Based on these assumptions, we plan to include 640 participants in total (320 DP, 320 placebo); of whom 384 are expected to be malaria infected, giving 192 participants in each trial arm who are infected at baseline.

Table 2 shows power estimates, for 5% significance level and assuming 20% loss to follow-up.

Table 2. Power estimates (5% significance level)

	**Log_{10} difference**						
**Standard deviation (log_{10})**	**0.08**	**0.10**	**0.12**	**0.14**	**0.16**	**0.18**	**0.20**
192 DP vs 192 placebo (malaria infected only)							
0.3	65%	83%	94%	98%	>99%	>99%	>99%
0.4	42%	59%	75%	87%	94%	98%	99%
0.5	29%	42%	56%	69%	80%	88%	94%
0.6	21%	31%	42%	53%	65%	75%	83%
Cells highlighted in grey correspond to >80% power.							

Ethics and dissemination

For peer review only - http://bmjopen.bmj.com/site/about/guidelines.xhtml

Ethical approval has been granted from the Research Ethics Committees of the Uganda Virus Research Institute (UVRI REC, reference: GC/127/19/05/681) and the London School of Hygiene and Tropical Medicine (LSHTM, reference: 16033), and from the Uganda National Council for Science and Technology (UNCST, reference: HS 2487) and the Uganda National Drug Authority (NDA, reference: CTC0117/2020). Any protocol amendments will be submitted to ethics committees and regulatory bodies for approval before implementation.

Participants will be adolescents and therefore a vulnerable human population. Care will be taken to provide adequate, age and education-status appropriate information and to ensure that it is understood; and to emphasise that participation is voluntary. Participants will be enrolled only when they have given their own assent and when consent has been given by the parent or guardian. No major risks to the participants are anticipated since all the treatments and vaccines to be given are licensed and known to be safe. The main risk to participants will be time lost from school work: we will work with teachers and parents to minimise disruption to classes, and will avoid enrolment of primary 7 students since these classes are involved in national examinations. Further risks are discussed in Supplementary information.

For this trial we gave particular consideration to the differential provision of malaria treatment. Intermittent preventive treatment with DP is an experimental intervention that has been shown to reduce the prevalence of anaemia and reduce episodes of clinical malaria in Ugandan schools[36] and was shown to be safe under suitable exclusion criteria[50] but has not been adopted as standard of care. To manage the expected differential benefits of the interventions for anaemia, a full blood count will be performed at baseline, as discussed above; anaemic children will be managed appropriately and severely anaemic children excluded. Additional safety monitoring during the trial will be done by the Data Safety and Monitoring Board (DSMB). Further information on the DSMB can be found in Supplementary information.

Malaria infection status will be determined retrospectively through assays conducted in bulk on stored samples (malaria PCR). These results will not, therefore, be useful to determine management of individual participants. Participants in the placebo arms will receive lower levels of anti-malaria treatment. However, all trial arms will receive a minimum of well-implemented national standard of care. Malaria standard of care will comprise provision of bed nets to minimise malaria exposure for all participants. Rapid diagnostic tests and treatment will be made readily available for participants who develop symptomatic malaria.

Study findings will be published through open-access peer-reviewed journals, presentations at local, national and international conferences and to the local community through community meetings. Anonymised participant level datasets generated will be available upon request.

Participant and public involvement

For peer review only - http://bmjopen.bmj.com/site/about/guidelines.xhtml

Concepts involved in this work have been discussed with colleagues at the Vector Control Division and Expanded Programme on Immunisation in the Ministry of Health (Uganda). Before commencing the trial, discussions will be held with officials at Jinja District Council, community leaders and Village Health Teams from sub-counties in which selected schools are located. In addition, we will engage teachers and parents in planning the detailed standard operating procedures for the study. Prior to recruitment we will hold meetings to explain the proposed work to teachers, parents and students and to address their questions. At the end of the study, results will be shared with these stakeholders and with participants.

Data management and analysis

Socio-demographic information and clinical and laboratory measurements will be recorded and managed using REDCap (Research Electronic Data Capture) tools,[38 39] with paper-based forms as back-up. All data will be recorded under a unique study ID number. When paper forms must be used, data will be double entered in a study-specific database, with standard checks for discrepancies. All data for analysis will be anonymised and stored on a secure and password-protected server, with access limited to essential research personnel.

The effect of monthly intermittent preventive treatment (IPT) for malaria versus no IPT on the outcomes will be analysed using unpaired t-tests, with results presented as a mean difference in vaccine response measure together with 95% confidence interval and p-value. In the event of any imbalance in key characteristics between trial arms, multivariable regression models will be used to adjust for these potential confounders. We anticipate that outcomes will be positively skewed, and will apply log transformations to normalise distributions before analysis is conducted. Information on malaria infection status will only be available after randomisation. The primary analysis will be done on individuals identified as infected at baseline (through the randomisation, these will be balanced between treatment arms); this will test the hypothesis that treating the infection (and subsequent reinfections) removes parasite-induced effects on vaccine responses. Secondary analyses will include all randomised individuals; this will provide insight into the potential benefit of the interventions as public health measures.

For peer review only - http://bmjopen.bmj.com/site/about/guidelines.xhtml

Discussion

Parasites have long been proposed as modulators of vaccine responses;[15 16] malaria is highly prevalent in settings with poor vaccine responses. Drivers of population differences in vaccine response are not fully elucidated; improved understanding is important for effective vaccine development and implementation. If treating malaria improves vaccine responses, combining parasite-control with immunisation programmes offers an attractive, practical public health intervention for schools and communities.

This study aims to determine whether treating malaria with DP before and after immunisation with unrelated vaccines can improve vaccine responses in adolescents. The use of DP in this study is considered an attractive option for preventive treatment and preventive chemotherapy for malaria because of the long half-life of piperaquine (approximately 23 days).[50] Despite identified benefits in reducing episodes of malaria and anaemia,[36] DP has not been adopted as standard of care either in Uganda or elsewhere. Although we hypothesise that treatment of malaria will improve vaccine responses, it is possible that it will negatively impact immune responses, or that effects will differ between types of vaccines. This study is expected to add further evidence regarding the potential benefits of monthly DP for school children by determining the effect, whether beneficial or negative, on vaccine responses, as well as collecting data on malaria infection status, thereby further contributing to policy debate in this field.

A concern arises where treatment of malaria with DP may have potential adverse effects such as cardiac toxicity, particularly dose-dependent prolongation of the QTc interval and associated increased risk of arrhythmias. There has been concern that provision of multiple doses might increase this risk especially when taken with food. However, a recent meta-analysis involving 11 trials of DP (9 IPT, 2 treatment trials) involving 14,628 participants, 3,935 of whom received multiple treatments with DP, found no evidence to suggest significant cardiac toxicity.[50] Few of the studies measured electrocardiographic changes, but those that did found no increase in QTc intervals with increasing numbers of doses. Therefore, the use of DP in this study where malaria prevalence is high is not expected to pose a significant risk to the participants. Also, although the use of DP necessitates strict exclusion criteria, based on previous studies in Uganda[22] it is not anticipated that many participants will meet these exclusion criteria.

We expect about 60% of the adolescents to have malaria infection at the outset in the proposed study area. This may vary by season and year, and the prevalence could be much lower depending on the timing of the start of the study. If this becomes the case, the two study arms will not be different enough to test our hypothesis. To mitigate this, we will work closely with colleagues monitoring trends in malaria prevalence in Uganda and adjust our study site to optimise the likelihood of achieving the expected prevalence.

This interventional study will help us to understand how malaria affects responses to commonly used vaccines, which will inform implementation of public health programmes for immunisation and infection control especially in low- and middle-income countries.

For peer review only - http://bmjopen.bmj.com/site/about/guidelines.xhtml

Study timeline

Applications for ethical approval were submitted in May 2018, with approval received in September 2018 (Uganda Virus Research Institute Research Ethics Committee), May 2019 (Uganda National Council for Science and Technology), June 2019 (London School of Hygiene and Tropical Medicine) and January 2020 (National Drug Authority). Collaborator/investigator/trial steering committee meetings were also held during the initial 12-month planning period. Recruitment to POPVAC B is scheduled to commence in February 2021. Intervention will be up to 12 months, with completion of the project scheduled for April 2022.

Competing interests

Alison Elliott reports a grant from the Medical research Council, UK (POPVAC programme funding). The rest of the authors declare that they have no conflicts of interest.

Author contributions

AME conceived the study. AME, AN, GN, ELW, SC, LZ and SGS contributed to study design. LZ, GO, PNK, SA, CO, RA, EN, and FA are site clinicians/nurses/clinical laboratory technicians providing valuable input on clinical considerations of the intervention. SK, FK, DN, MS and RK are field workers handling the organisational integration of the intervention. AN, AM and ELW are involved in organisation of the databases, trial randomization, treatment allocation and drawing up of analytical plans. AN, GN, LZ, JN, SC, ELW and AME drafted the manuscript. All authors reviewed the manuscript, contributed to it and approved the final version.

Acknowledgements

We thank the Uganda National Expanded Programme for Immunisation, Sanofi Pasteur and PaxVax for providing the HPV, yellow fever and oral typhoid vaccines, respectively. The BCG and tetanus-diphtheria vaccines were kind donations from the Serum Institute of India. We thank the Jinja district local government for their support. We also thank members of the POPVAC programme steering committee (chaired by Prof. Richard Hayes) and the Data and Safety Monitoring Board (Dr David Meya, Prof Andrew Prendergast and Dr Elizabeth George).

Funding

The POPVAC programme of work is supported by the Medical Research Council of the United Kingdom (grant number MR/R02118X/1). SC and JN are supported in part by the Makerere University – Uganda Virus Research Institute Centre of Excellence for Infection and Immunity Research and Training (MUII-plus). MUII-plus is funded under the DELTAS Africa Initiative. The DELTAS Africa Initiative is an independent funding scheme of the African Academy of Sciences (AAS), Alliance for Accelerating Excellence in Science in Africa

For peer review only

For peer review only - http://bmjopen.bmj.com/site/about/guidelines.xhtml

(AESA) and supported by the New Partnership for Africa's Development Planning and Coordinating Agency (NEPAD Agency) with funding from the Wellcome Trust (grant 107743) and the UK Government. The MRC/UVRI and LSHTM Uganda Research Unit is jointly funded by the UK Medical Research Council (MRC) and the UK Department for International Development (DFID) under the MRC/DFID Concordat agreement and is also part of the EDCTP2 programme supported by the European Union.

The study sponsor (London School of Hygiene and Tropical Medicine) and funders had no role in study design; collection, management, analysis, and interpretation of data; writing of the protocol; and the decision to submit the protocol for publication.

Data Sharing

Data sharing is not applicable as no datasets have been generated and/or analysed. When trial results become available, the de-identified individual participant data that underlie the results reported in journal articles will be stored in a non-publically available repository (LSHTM Data Compass, https://datacompass.lshtm.ac.uk), together with a data dictionary. This will be done at the time of publication. Each dataset will be allocated a unique digital object identifier (DOI). Researchers who would like to access the data may submit a request through LSHTM Data Compass, detailing the data requested, the intended use for the data, and evidence of relevant experience and other information to support the request. The request will be reviewed by the Principal Investigator in consultation with the POPVAC Steering Committee, with oversight from the UVRI and LSHTM ethics committees. In line with the MRC policy on Data Sharing, there will have to be a good reason for turning down a request. Patient Information Sheets and consent forms specifically referenced making anonymised data available and this has been approved by the relevant ethics committees. Researchers given access to the data will sign data sharing agreements which will restrict the use to answering pre-specified research questions.

POPVAC trial team

Principal investigator: Alison Elliott; ***Project leader***: Ludoviko Zirimenya; ***laboratory staff***: Gyaviira Nkurunungi, Stephen Cose, Rebecca Amongin, Beatrice Nassanga, Jacent Nassuuna, Irene Nambuya, Prossy Kabuubi, Emmanuel Niwagaba, Gloria Oduru, Grace Kabami; ***statisticians and data managers***: Emily Webb, Agnes Natukunda, Helen Akurut, Alex Mutebe; ***clinicians***: Anne Wajja, Milly Namutebi, Christopher Zziwa; Joel Serubanja; ***nurses***: Caroline Onen, Esther Nakazibwe, Josephine Tumusiime, Caroline Ninsiima, Susan Amongi, Florence Akello; ***internal monitor***: Mirriam Akello; ***field workers***: Robert Kizindo, Moses Sewankambo, Denis Nsubuga, Samuel Kiwanuka, Fred Kiwudhu; ***boatman***: David Abiriga; ***administrative management***: Moses Kizza, Samsi Nansukusa; ***internal and external collaborators:*** Pontiano Kaleebu,

For peer review only - http://bmjopen.bmj.com/site/about/guidelines.xhtml

For peer review only

431 Hermelijn Smits, Maria Yazdanbakhsh, Govert van Dam, Paul Corstjens, Sarah Staedke, Henry Luzze, James
432 Kaweesa, Edridah Tukahebwa, Elly Tumushabe, Moses Muwanga.

For peer review only

For peer review only - http://bmjopen.bmj.com/site/about/guidelines.xhtml

REFERENCES

1. Barreto ML, Pereira SM, Pilger D, et al. Evidence of an effect of BCG revaccination on incidence of tuberculosis in school-aged children in Brazil: second report of the BCG-REVAC cluster-randomised trial. *Vaccine* 2011;29(31):4875-7. doi: 10.1016/j.vaccine.2011.05.023

2. Barreto ML, Pilger D, Pereira SM, et al. Causes of variation in BCG vaccine efficacy: examining evidence from the BCG REVAC cluster randomized trial to explore the masking and the blocking hypotheses. *Vaccine* 2014;32(30):3759-64. doi: 10.1016/j.vaccine.2014.05.042

3. Black GF, Weir RE, Floyd S, et al. BCG-induced increase in interferon-gamma response to mycobacterial antigens and efficacy of BCG vaccination in Malawi and the UK: two randomised controlled studies. *Lancet* 2002;359(9315):1393-401. doi: 10.1016/S0140-6736(02)08353-8

4. Fine PE. Variation in protection by BCG: implications of and for heterologous immunity. *Lancet* 1995;346(8986):1339-45.

5. Taniuchi M, Platts-Mills JA, Begum S, et al. Impact of enterovirus and other enteric pathogens on oral polio and rotavirus vaccine performance in Bangladeshi infants. *Vaccine* 2016;34(27):3068-75. doi: 10.1016/j.vaccine.2016.04.080 [published Online First: 2016/05/08]

6. Muyanja E, Ssemaganda A, Ngauv P, et al. Immune activation alters cellular and humoral responses to yellow fever 17D vaccine. *The Journal of clinical investigation* 2014;124(7):3147-58. doi: 10.1172/jci75429 [published Online First: 2014/06/10]

7. van Riet E, Adegnika AA, Retra K, et al. Cellular and humoral responses to influenza in gabonese children living in rural and semi-urban areas. *The Journal of infectious diseases* 2007;196(11):1671-8. doi: 10.1086/522010 [published Online First: 2007/11/17]

8. van Riet E, Retra K, Adegnika AA, et al. Cellular and humoral responses to tetanus vaccination in Gabonese children. *Vaccine* 2008;26(29-30):3690-5. doi: 10.1016/j.vaccine.2008.04.067 [published Online First: 2008/06/10]

9. Tanner R, Kakalacheva K, Miller E, et al. Serum indoleamine 2,3-dioxygenase activity is associated with reduced immunogenicity following vaccination with MVA85A. *BMC infectious diseases* 2014;14:660. doi: 10.1186/s12879-014-0660-7 [published Online First: 2014/12/04]

10. Sissoko MS, Healy SA, Katile A, et al. Safety and efficacy of PfSPZ Vaccine against Plasmodium falciparum via direct venous inoculation in healthy malaria-exposed adults in Mali: a randomised, double-blind phase 1 trial. *The Lancet Infectious diseases* 2017;17(5):498-509. doi: 10.1016/s1473-3099(17)30104-4 [published Online First: 2017/02/22]

11. Venkatraman N, Ndiaye BP, Bowyer G, et al. Safety and Immunogenicity of a Heterologous Prime-Boost Ebola Virus Vaccine Regimen in Healthy Adults in the United Kingdom and Senegal. *The Journal of infectious diseases* 2019;219(8):1187-97. doi: 10.1093/infdis/jiy639

12. Rodrigues LC, Noel Gill O, Smith PG. BCG vaccination in the first year of life protects children of Indian subcontinent ethnic origin against tuberculosis in England. *Journal of epidemiology and community health* 1991;45(1):78-80. doi: 10.1136/jech.45.1.78 [published Online First: 1991/03/01]

13. Flaherty DK, Vesosky B, Beamer GL, et al. Exposure to Mycobacterium avium can modulate established immunity against Mycobacterium tuberculosis infection generated by Mycobacterium bovis BCG vaccination. *Journal of leukocyte biology* 2006;80(6):1262-71. doi: 10.1189/jlb.0606407 [published Online First: 2006/09/14]

14. Brandt L, Feino Cunha J, Weinreich Olsen A, et al. Failure of the Mycobacterium bovis BCG vaccine: some species of environmental mycobacteria block multiplication of BCG and induction of protective immunity to tuberculosis. *Infection and immunity* 2002;70(2):672-8. [published Online First: 2002/01/18]

15. Bentwich Z, Kalinkovich A, Weisman Z, et al. Can eradication of helminthic infections change the face of AIDS and tuberculosis? *Immunol Today* 1999;20(11):485-7.

For peer review only

For peer review only - http://bmjopen.bmj.com/site/about/guidelines.xhtml

16. Cunnington AJ, Riley EM. Suppression of vaccine responses by malaria: insignificant or overlooked? *Expert review of vaccines* 2010;9(4):409-29. doi: 10.1586/erv.10.16 [published Online First: 2010/04/08]

17. Sanya RE, Nkurunungi G, Andia Biraro I, et al. A life without worms. *Transactions of the Royal Society of Tropical Medicine and Hygiene* 2017:1-9. doi: 10.1093/trstmh/trx010 [published Online First: 2017/03/25]

18. WHO. World Malaria Report. France, 2019.

19. Rugnao S, Gonahasa S, Maiteki-Sebuguzi C, et al. LLIN Evaluation in Uganda Project (LLINEUP): factors associated with childhood parasitaemia and anaemia 3 years after a national long-lasting insecticidal net distribution campaign: a cross-sectional survey. *Malaria journal* 2019;18(1):207-07. doi: 10.1186/s12936-019-2838-3

20. Geneva: World Health Organization. World Malaria Report 2019; https://www.who.int/publications-detail/world-malaria-report-2019.

21. Rehman AM, Maiteki-Sebuguzi C, Gonahasa S, et al. Intermittent preventive treatment of malaria delivered to primary schoolchildren provided effective individual protection in Jinja, Uganda: secondary outcomes of a cluster-randomized trial (START-IPT). *Malar J* 2019;18(1):318. doi: 10.1186/s12936-019-2954-0 [published Online First: 2019/09/20]

22. Staedke SG, Maiteki-Sebuguzi C, Rehman AM, et al. Assessment of community-level effects of intermittent preventive treatment for malaria in schoolchildren in Jinja, Uganda (START-IPT trial): a cluster-randomised trial. *The Lancet Global Health* 2018;6(6):e668-e79. doi: https://doi.org/10.1016/S2214-109X(18)30126-8

23. Greenwood BM, Bradley AK, Blakebrough IS, et al. The immune response to a meningococcal polysaccharide vaccine in an African village. *Transactions of the Royal Society of Tropical Medicine and Hygiene* 1980;74(3):340-6. doi: 10.1016/0035-9203(80)90095-4 [published Online First: 1980/01/01]

24. McGregor IA, Barr M. Antibody response to tetanus toxoid inoculation in malarious and non-malarious Gambian children. *Transactions of the Royal Society of Tropical Medicine and Hygiene* 1962;56(5):364-67. doi: 10.1016/0035-9203(62)90005-6

25. Greenwood AM, Greenwood BM, Bradley AK, et al. Enhancement of the immune response to meningococcal polysaccharide vaccine in a malaria endemic area by administration of chloroquine. *Annals of Tropical Medicine and Parasitology* 1981;75(2):261-63.

26. Williamson WA, Greenwood BM. IMPAIRMENT OF THE IMMUNE RESPONSE TO VACCINATION AFTER ACUTE MALARIA. *The Lancet* 1978;311(8078):1328-29. doi: 10.1016/S0140-6736(78)92403-0

27. Bradley-Moore AM, Greenwood BM, Bradley AK, et al. Malaria chemoprophylaxis with chloroquine in young Nigerian children. II. Effect on the immune response to vaccination. *Ann Trop Med Parasitol* 1985;79(6):563-73. doi: 10.1080/00034983.1985.11811963 [published Online First: 1985/12/01]

28. Kizito D, Tweyongyere R, Namatovu A, et al. Factors affecting the infant antibody response to measles immunisation in Entebbe-Uganda. *BMC Public Health* 2013;13(1):619. doi: 10.1186/1471-2458-13-619

29. Elliott AM, Mawa PA, Webb EL, et al. Effects of maternal and infant co-infections, and of maternal immunisation, on the infant response to BCG and tetanus immunisation. *Vaccine* 2010;29(2):247-55. doi: 10.1016/j.vaccine.2010.10.047

30. Brown J, Baisley K, Kavishe B, et al. Impact of malaria and helminth infections on immunogenicity of the human papillomavirus-16/18 AS04-adjuvanted vaccine in Tanzania. *Vaccine* 2014;32(5):611-17. doi: https://doi.org/10.1016/j.vaccine.2013.11.061

31. Crawley J, Sismanidis C, Goodman T, et al. Effect of intermittent preventive treatment for malaria during infancy on serological responses to measles and other vaccines used in the Expanded Programme on Immunization: results from five randomised controlled trials. *Lancet* 2012;380(9846):1001-10. doi: 10.1016/s0140-6736(12)60775-2 [published Online First: 2012/08/02]

For peer review only

For peer review only - http://bmjopen.bmj.com/site/about/guidelines.xhtml

518 32. Brown J, Baisley K, Kavishe B, et al. Impact of malaria and helminth infections on immunogenicity of the human
519 papillomavirus-16/18 AS04-adjuvanted vaccine in Tanzania. *Vaccine* 2014;32(5):611-7. doi:
520 10.1016/j.vaccine.2013.11.061

521 33. Usen S, Milligan P, Ethevenaux C, et al. Effect of fever on the serum antibody response of Gambian children to
522 Haemophilus influenzae type b conjugate vaccine. *The Pediatric infectious disease journal* 2000;19(5):444-9.
523 [published Online First: 2000/05/20]

524 34. Obeng-Adjei N, Portugal S, Tran TM, et al. Circulating Th1-Cell-type Tfh Cells that Exhibit Impaired B Cell Help Are
525 Preferentially Activated during Acute Malaria in Children. *Cell reports* 2015;13(2):425-39. doi:
526 10.1016/j.celrep.2015.09.004 [published Online First: 2015/10/07]

527 35. Chan AW, Tetzlaff JM, Altman DG, et al. SPIRIT 2013 statement: defining standard protocol items for clinical trials.
528 *Ann Intern Med* 2013;158(3):200-7. doi: 10.7326/0003-4819-158-3-201302050-00583 [published Online First:
529 2013/01/09]

530 36. Nankabirwa JI, Wandera B, Amuge P, et al. Impact of intermittent preventive treatment with dihydroartemisinin-
531 piperaquine on malaria in Ugandan schoolchildren: a randomized, placebo-controlled trial. *Clinical infectious diseases*
532 *: an official publication of the Infectious Diseases Society of America* 2014;58(10):1404-12. doi: 10.1093/cid/ciu150
533 [published Online First: 2014/03/14]

534 37. World Health Organization. Guidelines for the treatment of malaria – 3rd edition., 2015.

535 38. Harris PA, Taylor R, Minor BL, et al. The REDCap Consortium: Building an International Community of Software
536 Platform Partners. *Journal of biomedical informatics* 2019:103208. doi: 10.1016/j.jbi.2019.103208 [published Online
537 First: 2019/05/13]

538 39. Harris PA, Taylor R, Thielke R, et al. Research electronic data capture (REDCap)--a metadata-driven methodology
539 and workflow process for providing translational research informatics support. *Journal of biomedical informatics*
540 2009;42(2):377-81. doi: 10.1016/j.jbi.2008.08.010 [published Online First: 2008/10/22]

541 40. Forrest BD. Identification of an intestinal immune response using peripheral blood lymphocytes. *Lancet*
542 1988;1(8577):81-3. doi: 10.1016/s0140-6736(88)90284-x [published Online First: 1988/01/16]

543 41. Lubyayi L, Mawa PA, Nabakooza G, et al. Maternal Latent Mycobacterium tuberculosis Does Not Affect the Infant
544 Immune Response Following BCG at Birth: An Observational Longitudinal Study in Uganda. *Frontiers in Immunology*
545 2020;11(929) doi: 10.3389/fimmu.2020.00929

546 42. Santos APd, Bertho ÁL, Dias DC, et al. Lymphocyte subset analyses in healthy adults vaccinated with yellow fever
547 17DD virus. *Memórias do Instituto Oswaldo Cruz* 2005;100:331-37.

548 43. Soares AP, Kwong Chung CKC, Choice T, et al. Longitudinal changes in CD4(+) T-cell memory responses induced by
549 BCG vaccination of newborns. *The Journal of infectious diseases* 2013;207(7):1084-94. doi: 10.1093/infdis/jis941
550 [published Online First: 2013/01/04]

551 44. Valentini D, Rao M, Rane L, et al. Peptide microarray-based characterization of antibody responses to host proteins
552 after bacille Calmette–Guérin vaccination. *International Journal of Infectious Diseases* 2017;56:140-54. doi:
553 https://doi.org/10.1016/j.ijid.2017.01.027

554 45. Plotkin SA. Correlates of protection induced by vaccination. *Clinical and vaccine immunology : CVI* 2010;17(7):1055-
555 65. doi: 10.1128/cvi.00131-10 [published Online First: 2010/05/14]

556 46. Corstjens PL, Nyakundi RK, de Dood CJ, et al. Improved sensitivity of the urine CAA lateral-flow assay for diagnosing
557 active Schistosoma infections by using larger sample volumes. *Parasites & vectors* 2015;8:241. doi: 10.1186/s13071-
558 015-0857-7 [published Online First: 2015/04/22]

559 47. Fletcher HA, Snowden MA, Landry B, et al. T-cell activation is an immune correlate of risk in BCG vaccinated infants.
560 *Nat Commun* 2016;7:11290. doi: 10.1038/ncomms11290

For peer review only - http://bmjopen.bmj.com/site/about/guidelines.xhtml

561 48. Safaeian M, Porras C, Pan Y, et al. Durable antibody responses following one dose of the bivalent human papillomavirus L1 virus-like particle vaccine in the Costa Rica Vaccine Trial. *Cancer prevention research (Philadelphia, Pa)* 2013;6(11):1242-50. doi: 10.1158/1940-6207.capr-13-0203 [published Online First: 2013/11/06]

564 49. Tweyongyere R, Nassanga BR, Muhwezi A, et al. Effect of Schistosoma mansoni infection and its treatment on antibody responses to measles catch-up immunisation in pre-school children: A randomised trial. *PLoS neglected tropical diseases* 2019;13(2):e0007157. doi: 10.1371/journal.pntd.0007157 [published Online First: 2019/02/15]

567 50. Gutman J, Kovacs S, Dorsey G, et al. Safety, tolerability, and efficacy of repeated doses of dihydroartemisinin-piperaquine for prevention and treatment of malaria: a systematic review and meta-analysis. *The Lancet Infectious diseases* 2017;17(2):184-93. doi: 10.1016/s1473-3099(16)30378-4 [published Online First: 2016/11/21]

572 **FIGURE LEGENDS**

573 **Figure 1. Outline of immunisations and interventions**

574 [1]Primary endpoints will be at eight weeks post BCG and four weeks post Yellow fever (YF-17D), oral typhoid (Ty21a), Human Papilloma Virus (HPV) and Tetanus/diptheria (Td) vaccination.

576 [2]Primary endpoint for responses to Td given at 28 weeks.

For peer review only

For peer review only - http://bmjopen.bmj.com/site/about/guidelines.xhtml

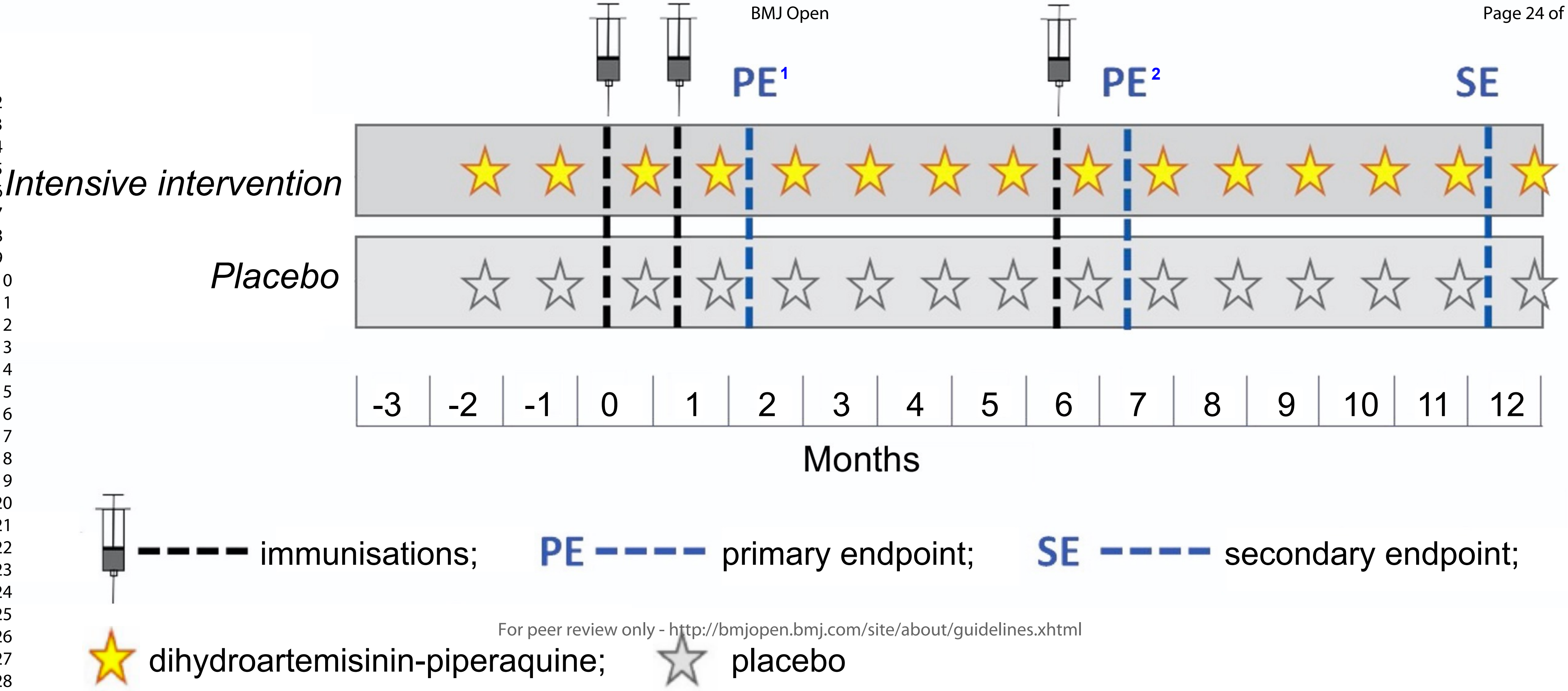
PE1
PE2
SE
Intensive intervention
Placebo
-3
-2
-1
0
1
2
3
4
5
6
7
8
9
10
11
12
Months
immunisations;
PE primary endpoint;
SE secondary endpoint;
dihydroartemisinin-piperaquine;
placebo

SUPPLEMENTARY INFORMATION

The effect of intermittent preventive treatment for malaria with dihydroartemisinin-piperaquine on immune responses to vaccines among rural Ugandan adolescents: randomised controlled trial protocol B for the 'POPulation differences in VACcine responses' (POPVAC) programme

Agnes Natukunda[1,¶], Ludoviko Zirimenya[1,¶], Gyaviira Nkurunungi[1,¶,*], Jacent Nassuuna[1], Gloria Oduru[1], Rebecca Amongin[1], Prossy N Kabuubi[1], Alex Mutebe[1], Caroline Onen[1], Susan Amongi[1], Esther Nakazibwe[1], Florence Akello[1], Samuel Kiwanuka[1], Fred Kiwudhu[1], Moses Sewankambo[1], Denis Nsubuga[1], Robert Kizindo[1], Sarah G Staedke[2,3], Stephen Cose[1,3], Emily L Webb[4], Alison M Elliott[1,3] **for the POPVAC trial team**

[1]Immunomodulation and Vaccines Programme, Medical Research Council/Uganda Virus Research Institute and London School of Hygiene and Tropical Medicine (MRC/UVRI and LSHTM) Uganda Research Unit, Entebbe, Uganda

[2]Infectious Diseases Research Collaboration, Kampala, Uganda

[3]Department of Clinical Research, London School of Hygiene and Tropical Medicine, London, United Kingdom

[4]MRC Tropical Epidemiology Group, Department of Infectious Disease Epidemiology, London School of Hygiene and Tropical Medicine, London, United Kingdom

[¶]These authors contributed equally

***Correspondence:** Gyaviira Nkurunungi; Gyaviira.Nkurunungi@mrcuganda.org

For peer review only

21 SUPPLEMENTARY INFORMATION

Table S1. POPVAC B: schedule of visits and procedures

VISIT NUMBER	1	2, 3	4	5	6[9]	6.2	7	8	9, 10, 11, 12, 13	14	15	16	17,18, 19, 20, 21	22
WEEKS FROM 1ST IMMUNISATION	-8[1]	-6[10], -2	0	2	4	4 + 4 days	6	8	10, 14, 18, 22 , 26	28	30	32	34, 38, 42, 46, 50	52
	Screening	Treatment (Rx only)	Immunisation	Treatment (Rx only)	Immunisations		Rx only	Primary endpoint (PE)	Rx only	Immunisations	Rx only	PE[2]	Rx only	Secondary endpoint (SE)
**RANDOMISED DIHYDROARTEMISININ-PIPERAQUINE INTERVENTION**														
DP treatment arm (x)		x		x			x		x		x		x	
DP control arm (o represents placebo)		o		o			o		o		o		o	
Albendazole								x[3]				x[3]		x[3]
Praziquantel								x[3,6]						x[3,6]
**VACCINES**														
BCG			x											
YF-17D					x									
Ty21a					x									
HPV					x			[x][4]		x				
Td										x				[x][5]
**INVESTIGATIONS/PROCEDURES**														
Inclusion/exclusion criteria	x													
Informed consent	x													
Questionnaire	x		x		x	x		x		x		x		x
Examination	x		(x)		(x)	(x)		(x)		(x)		(x)		(x)
Urine β-HCG test (female only) 1mL	x		x		x					x				
Urine YF viral load						x								
Stool for PCR and storage	x									x				x
Stool for coproantibody and storage	x							x						
**BLOOD SAMPLES**														
Malaria PCR (1ml)	x		x		x			x		x		x		x
Serology for HIV, prior malaria and *S. mansoni* (0.5ml)	x													
Mansonella perstans (1ml)	x													
Serum/plasma CAA (1ml)	x									x				x
Hb[8]/Full blood count (0.5ml)	x		x		x					x				
Assessments of pre-immunisation responses, and/or vaccine response outcomes and/or exploratory immunology; storage[7] (10-20ml)			x		x			x		x		x		x
Blood for gene expression (2ml)			x		x					x				
Blood vol (mL)	4		27		17			14-24		27		14		14

Cumulative blood vol (mL)[7]	4		31		48			72		99		113		127

PE: primary endpoint; **SE:** secondary endpoint; **Rx only**: treatment only
Immunisation days highlighted in green, primary end point days in red, days for treatment only in grey
(**x**) performed if clinically indicated

1. Screening and enrolment into Project B will take place at least 8 weeks before immunisation 0 to allow initiation of the DP intervention.
2. Week 32 is primary endpoint for responses to Td given at 28 weeks; secondary endpoint for HPV booster
3. Treatments given after sampling when schedules coincide
4. Week 8 HPV dose will be given for previously-unvaccinated girls aged $\geq$14 years
5. Week 52 Td booster dose will be provided as a service
6. Praziquantel if required in accordance with Ministry of Health policy for the selected schools
7. Exploratory immunology blood volume will be guided by guidelines from Harvard Mass General, where a maximum of 3ml/kg body weight is taken at any one time point and not more than 3ml/kg is taken over any 8 week period (ref http://www.drgreene.com/21_1616.html.) These guidelines have been followed in a previous study vaccinating adolescents with investigational tuberculosis vaccine MVA85A (in Uganda).[1] The total blood volume planned is 72 ml over the initial intensive sampling period of 8 weeks. Revision of sample volumes based on weight will only be required for participants who weigh less than 24 kg; the average weight of children aged 9 years is expected to be 28kg (with 21kg the 3rd centile) with greater weights for older children.[2]
8. At Baseline, it will only be Hb estimation by Haemocue
9. Oral typhoid vaccine doses will be administered on four alternate days namely visit 6, 6.1, 6.2 and 6.3.
10. The first DP and Placebo administration at week -6 will be administered at the end of the screening visit

For peer review only

Further information on the recruitment criteria

- Participants who are excluded from the trial because they have been discovered during screening procedures to be suffering from a previously undiagnosed condition thought to require further medical attention will be referred appropriately for further investigation and treatment.
- Participants discovered to have severe anaemia will be excluded from the trial and treated for anaemia and any evident underlying cause.
- Participants discovered to be HIV-positive will be counselled and offered a CD4 T-cell count and referred to a provider for further care and antiretroviral treatment (since this is indicated for all HIV-positive children under national guidelines).
- Participants discovered to be pregnant will be counselled and referred to an antenatal clinic of their choice.

This trial proposes to recruit all participants within a short time-frame. It will not, therefore, be possible to reconsider enrolment of potential participants with temporary exclusion criteria after treatment and resolution of the condition.

Further rationale for the selection of vaccines

Bacillus Calmette-Guérin (BCG)

BCG is a live, replicating parenteral vaccine, the only licensed vaccine against TB. BCG vaccine for these studies will be obtained from the Serum Institute of India either directly, or through a supplier in Uganda. The Serum Institute of India provides much of the BCG vaccine used in Uganda.

Worldwide, TB is among the top 10 causes of death; Uganda has an estimated incidence of 202/100,000 people.[3] Infectious, sputum positive, pulmonary TB classically emerges in adolescence, driving the on-going epidemic.[4] Thus adolescent booster immunisation is a key TB control strategy.[5] However, as discussed above, BCG vaccine response and efficacy are often impaired in tropical and rural settings[6-8] and new TB vaccines are similarly affected.[9] In the past, WHO has been hesitant to recommend BCG re-vaccination. However, in 2017 WHO's Strategic Advisory Group of Experts (SAGE) recommended, "Further research is warranted to explore whether certain sub-groups of age, geographic or *M. tuberculosis* exposure categories would benefit from re-vaccination."[10] Recent results suggest that, despite the variability of BCG efficacy between populations, BCG vaccination in adolescence offers benefit in some tropical settings, especially for individuals who are not yet infected with *Mycobacterium tuberculosis*, and may also be cost-effective.[7 11] Also, BCG vaccine is currently being used among adolescents in South Africa as a comparator in a trial of a novel TB vaccine (trial registration NCT02075203). To our knowledge, BCG efficacy in Ugandan adolescents, and differences in BCG vaccine responses between urban and rural Ugandan populations, have not been tested. Information obtained from this study is expected to further inform the use of BCG in adolescents, and also to inform the development of new vaccines for tuberculosis.

For peer review only

Yellow fever vaccine

Yellow fever vaccine YF-17D is a live replicating parenteral vaccine. The vaccine (Stamaril; Sanofi Pasteur) is available for purchase in Uganda. Yellow Fever (YF) causes outbreaks in Uganda and the wider region[12] and YF-17D is a candidate for Uganda's expanded programme on immunisation. Lower vaccine replication, lower neutralising antibody induction, and greater waning, are described in Uganda compared to Switzerland.[13] YF-17D is a potential vector for novel vaccine constructs,[14] adding relevance to vaccine development.

Typhoid vaccine Ty21a

Typhoid vaccine Ty21a is a live replicating oral vaccine and also a potential vector for new vaccine constructs.[15] Ty21a vaccine will be purchased from PaxVax, Redwood City, California. Substantial, multi-year typhoid outbreaks occur in Uganda and immunisation campaigns have been advocated as cost effective.[16] Schistosomiasis has been associated with prolonged S. typhi infection[17] and impaired antibody responses to killed typhoid vaccines.[18]

Ty21a was developed in the 1970s. Although not routinely used in Uganda, it has been (and is currently) registered in many countries. It was first registered in the United States and United Kingdom in the 1980s, and is recommended by the World Health Organisation for both endemic and epidemic settings.[19] It has comparable efficacy to the parenteral Vi polysaccharide typhoid vaccine, good durability and minimal adverse effects.[19] It is proposed for use in this study to model effects of study exposures and intervention on the response to a live oral vaccine.

The Ty21a vaccine is given as a three-dose regimen on alternate days.

Human Papilloma Virus (HPV) vaccine

Human Papilloma Virus (HPV) vaccine is a protein virus-like particle. The quadrivalent HPV Vaccine Gardasil (Merck) is available for purchase in Uganda and is the vaccine used by the national EPI programme. Studies after three vaccine doses have found somewhat enhanced responses in the presence of malaria, but no effect of helminths.[20] No study has previously investigated parasite effects on the priming response, but recent results for tetanus suggest that priming may be more susceptible than boosting to adverse effects.[21] This will be important if forthcoming trials support single-dose HPV immunisation (NCT02834637). HPV immunisation is being rolled out among girls to prevent cervical neoplasia, the commonest cancer among Ugandan women and we will coordinate provision with the national HPV immunisation programme.[22] HPV immunisation is also beneficial for boys since HPV infection is associated with anogenital warts, anal cancer and oropharyngeal cancers in both males and females, and with penile cancer in men,[23] and we will include boys in these studies.

For peer review only

Tetanus and diphtheria vaccines

Tetanus and diphtheria vaccines comprise inert toxoids (Td). Booster immunisation is recommended for young women to prevent maternal and neonatal tetanus. Recent evidence emphasises the need to protect young men also.[24] Uganda's EPI programme recommends tetanus boosters in adolescence and plans to change from tetanus alone to Td in 2018.

Additional considerations regarding the vaccine schedule

Live vaccines given in combination may influence the response to each other – a phenomenon described as "interference". Observations in the 1960s suggested that elevated circulating interferon (IFN)-γ after measles immunisation might interfere with the response to Vaccinia[25] and, to avoid such interference between live vaccines, it was recommended that live vaccines be given either together or three to four weeks apart.[26] However, with the introduction of new, live vaccines into use, Public Health England reviewed and revised this recommendation in 2014, limiting it to vaccines for which there was an evidence base (**Table S2**). We have adopted a four-week interval between BCG immunisation and the other proposed live vaccines (YF and Ty21a), which will be given together.[27]

Non-live vaccines can be given at the same time as live vaccines and there are no specific recommendations as to the number of non-live vaccines that can be given together. Two injections can be given into the same muscle although it is suggested that these should be at least 2.5 cm apart in case it is necessary to distinguish local adverse reactions to the two injections.[28] Our schedule avoids giving more than two injections on the same date but at week 4 it may be appropriate to give two into the deltoid muscle of the same arm if a BCG scar is developing on the other arm. Nevertheless, uncertainties remain regarding the effects of vaccines on responses to each other. Generalisations from this programme of work will need to take potential "interference" between vaccines into account.

For peer review only

Table S2: Public Health England recommendations for giving more than one live attenuated vaccine in current use in the UK[29]

Vaccine combinations	Recommendations
Yellow Fever and Measles, Mumps, Rubella (MMR)	A four week minimum interval period should be observed between the administration of these two vaccines. Yellow Fever and MMR should not be administered on the same day.
Varicella (and zoster) vaccine and MMR	If these vaccines are not administered on the same day, then a four week minimum interval should be observed between vaccines.
Tuberculin skin testing (Mantoux) and MMR	If a tuberculin skin test has already been initiated, then MMR should be delayed until the skin test has been read unless protection against measles is required urgently. If a child has had a recent MMR, and requires a tuberculin test, then a four week interval should be observed.
All currently used live vaccines (BCG, rotavirus, live attenuated influenza vaccine (LAIV), oral typhoid vaccine, yellow fever, varicella, zoster and MMR) and tuberculin (Mantoux) skin testing.	Apart from those combinations listed above, these live vaccines can be administered at any time before or after each other. This includes tuberculin (Mantoux) skin testing.

Immunisation Postponement Criteria

If any one of the following is identified at the time scheduled for immunisation, the participant may be immunised at a later date, or withdrawn, at the discretion of the Investigator. The participant must be followed until resolution of the event as with any adverse event:

- Acute disease at the time of immunisation. Acute disease is defined as the presence of a moderate or severe illness with or without fever. All vaccines can be administered to persons with a minor illness such as diarrhoea or mild upper respiratory infection with or without low-grade fever, i.e. temperature of ≤37.5°C (99.5°F)
- Temperature of >37.5°C (99.5°F) at the time of immunisation
- Taking antibiotics or antimalarials currently, or within the past 7 days, of the date of Ty21a administration (ascertained verbally)

Vaccine storage and transport

In order to maintain a reliable vaccine cold chain, the vaccines and diluents to be used will be stored and transported within the recommended temperature range of +2°C to +8°C. Care will be taken to ensure that the vaccines are not frozen. BCG, being sensitive to light, will be kept in the dark (normally within its secondary packaging) for as long as possible to protect it during storage and transportation. All vaccines will be kept in appropriate refrigeration equipment with a temperature monitoring device to ensure temperatures remain between +2°C and +8°C. Cold boxes/vaccines carriers with temperature monitors will be used to transport vaccines and the diluents from the MRC/UVRI and LSHTM Uganda Research Unit (Entebbe) to Jinja and while transporting vaccines to immunisation sessions. Designated staff will be given responsibility for managing the vaccine cold chain. All cold chain equipment including the temperature monitoring devices used for this project will comply with relevant technical specifications as defined by the EPI standards. Basic routine maintenance will be regularly carried out on all cold chain equipment.

Additional laboratory measurements

Additional assays will comprise HIV serology, pregnancy testing and full blood counts. HIV testing and pregnancy testing will be accompanied by appropriate counselling by trained staff.

HIV serology will be done on blood samples using rapid tests and according to prevailing national algorithms.[30] This will be done at baseline.

- Pregnancy testing will be done using urine samples and standard operating procedures for assessment of urine β-human chorionic gonadotropin (βhCG). This will be done at baseline and before immunisation on each immunisation day.
- Full blood counts will be conducted using a haematology analyser. Mild, moderate and severe anaemia will be defined according to WHO guidelines, by age.[31] This will be done at baseline to test

For peer review only

for anaemia as part of the eligibility assessment, and pre-immunisation as part of the assessment of immunological profile.

Individuals found to be HIV positive or pregnant will be referred to appropriate providers for further care. Individuals with severe anaemia (haemoglobin <82g/L) will be excluded from the randomised intervention (since the intervention might be beneficial in management of anaemia). They will be treated for anaemia and for any underlying cause identified.

Sample handling and archive

Blood and other samples will be processed according to local laboratory standard operating procedures (SOPs). All samples will reach the laboratory in anonymised form.

A sample archive will be developed. Although our current programme of work will address specific hypotheses regarding pathways of effects of parasites and interventions, the sample archive will provide a major asset for exploration of new leads arising from this work, or for an alternative, “systems biology” approach employing (for example) proteomic, genomic, epigenetic and transcriptomic analyses, and investigating the microbiome and virome. Information provided to participants, and consent forms, will include considerations of sample storage, and the possibility of sample analysis in laboratories within and outside Uganda. Participants will be able to decide if they will permit such future use of any leftover samples. We plan to store the samples for up to 20 years. If further storage is needed after that time, permission will be requested from the Uganda Virus Research Institute and London School of Hygiene and Tropical Medicine ethical review committees. If they elect not to permit this, all of those leftover samples will be discarded after the completion of the work included in the current protocol.

For peer review only

Operational considerations

Programme governance

A Programme Steering Committee has been set up to guide progress across all projects. This comprises the following:

- An independent chair
- Representatives from the Ministry of Health programmes for immunisation and for vector borne disease control
- Representatives of district authorities (Mukono and Jinja districts)
- Community representatives
- Principal investigator and co-investigators
- Project leader and post-doctoral immunologist
- Trial statistician
- Laboratory manager

179 • Medical Research Council observer

Informed consent

Both written informed assent from the participants and written informed consent from a parent or guardian will be required for participation, although these may not necessarily be obtained at the same time. Information will be provided in both English and the appropriate local language. For individuals who cannot speak the languages used, or who cannot read or write, a witness who can read the information sheet and translate the information to the participant or parent/guardian will be used. Two different types of age specific assent forms will be used for the group of participants aged 9 – 12 years and for the group aged 13 – 17 years. Informed consent by emancipated or mature minors will be obtained using a designated consent form for these categories of participants

The aims of the study, all tests, treatments and immunisations to be carried out and potential risks will be explained. The participant will be given the opportunity to ask about details of the trial, and will then have time to consider whether or not to participate. If they do decide to participate, they and their parent/guardian will sign and date two copies of the assent and consent forms, one for them to take away and keep, and one to be stored securely by the research team. Separate information and consent forms will be provided (i) for consent for storage of samples for future studies and for anonymous sharing of data from this study and (ii) for possible genetic studies; the information sheet will explain that these data may be used in analyses related to this protocol.

Screening and Eligibility Assessment

Once the informed consent process has been completed, and consent (and assent) given, a baseline medical history (including concomitant medication) will be collected. Vital signs will be checked and a physical examination will be performed. Inclusion and exclusion criteria will be checked.

Participants will undergo pre- and post-test counselling for HIV and (for girls) pregnancy testing by a trained and experienced nurse- or clinician-counsellor. Blood, urine and stool samples will be obtained, for tests as specified in the schedule of procedures. These tests are to exclude the major, immunomodulating co-infection, HIV, and conditions that might impact safety (anaemia, pregnancy).

Enrolment

Participants who consent/assent, complete the screening processes, satisfy all the inclusion criteria and meet none of the exclusion criteria will be enrolled.

Discontinuation / withdrawal criteria

In accordance with the principles of the current revision of the Declaration of Helsinki and any other applicable regulations, a participant has the right to withdraw from the study at any time and for any reason, and is not obliged to give his or her reasons for doing so. The Investigator may withdraw the

For peer review only

participant at any time in the interests of the participant's health and well-being. In addition, the participant may withdraw/be withdrawn for any of the following reasons:

- Ineligibility (either arising during the study or retrospectively, having been overlooked at screening)
- Administrative decision by the Investigator
- Significant protocol deviation
- Participant non-compliance with study requirements
- An adverse event which requires discontinuation of the study involvement or results in inability to continue to comply with study procedures.

Any participant who becomes pregnant during the trial will be followed up until the end of the pregnancy but no further immunisations will be given unless indicated during pregnancy (as is the case for tetanus toxoid). The trial allocation for this participant will be unblinded and the participant will only be given further treatment if clinically indicated. The babies will also be followed up and examined for any adverse effects. We will not routinely perform venepuncture in a pregnant participant.

The reason for withdrawal will be recorded in the case report form (CRF). If withdrawal is due to an AE, appropriate follow-up visits or medical care will be arranged, with the agreement of the participant, until the AE has resolved, stabilised or a non-trial related causality has been assigned.

If a participant withdraws from the study samples collected before their withdrawal from the trial will be used/ stored unless the participant specifically requests otherwise.

Trial discontinuation

The trial will be discontinued in the event of new scientific information that renders continuation futile or unethical, or for any other reason, at the discretion of the Programme Steering Committee.

End of study definition

The trial will be completed when the last participant enrolled into the trial has completed their final follow up visit.

Safety assessments and oversight

No new investigational drug or product will be used in the proposed trial. However, standard approaches for monitoring safety and reporting of serious adverse events will be followed.

Monitoring

The trial will be monitored by both internal and external monitors according to a pre-defined monitoring plan which will include a site initiation visit, monitoring visits at least annually, and a close-out visit. The monitors will assess patient safety, data integrity, and adherence to the protocol and to Good Clinical Research Practice procedures.

For peer review only

Considerations regarding standard of care

Malaria infection status will be determined retrospectively through assays conducted in bulk on stored samples (malaria PCR). These results will not, therefore, be useful to determine management of individual participants.

Participants in the placebo arms will receive lower levels of anti-malaria treatment. However, all trial arms will receive a minimum of well-implemented national standard of care.

Dihydroartemisinin/piperaquine is considered an attractive option for preventive treatment and preventive chemotherapy for malaria because of the long half-life of piperaquine (approximately 23 days).[32] Monthly treatment with DP has been shown to reduce the prevalence of anaemia and reduce episodes of clinical malaria in Ugandan schools[33] but has not been adopted as standard of care. This programme is expected to add further evidence regarding the potential benefits of monthly DP for school children by determining the effect on vaccine responses, thereby further contributing to policy debate in this field. To manage the expected differential benefits of the interventions for anaemia, a full blood count will be performed at baseline, as discussed above; anaemic children will be managed appropriately and severely anaemic children excluded.

Malaria standard of care will comprise provision of bed nets to minimise malaria exposure for all participants. Rapid diagnostic tests and treatment will be made readily available for participants who develop symptomatic malaria.

Albendazole will be provided twice a year to manage nematode infections (after collection of primary and secondary endpoint samples).

Procedures to be followed in the event of abnormal findings

Abnormal clinical findings from medical history, examination or blood tests will be assessed as to their clinical significance throughout the trials. If an abnormal test result is deemed clinically significant, it may be repeated. If a test remains clinically significant, the participant will be informed and appropriate medical care arranged as appropriate and with the permission of the participant. Specific details regarding findings, discussion with participants and resulting actions will be recorded in the clinical records. Decisions to exclude the participant from enrolling in the trial or to withdraw a participant from the trial will be at the discretion of the Investigator.

Data and Safety Monitoring Board (DSMB)

A data and safety monitoring board (DSMB) will be appointed to provide real-time safety oversight. The DSMB will be notified within 7 days of the Investigators' being aware of the occurrence of SAEs. The DSMB may recommend the Investigators to place the trial on hold if deemed necessary following an intervention-related SAE. The DSMB will be chaired by a clinician experienced in clinical trials. There will be a minimum

For peer review only

of two other appropriately qualified committee members. In the case of events related to a blinded intervention, the DSMB can request unblinding. Membership will include a statistician, and at least one Ugandan member. All correspondence between Investigators and the DSMB will be conveyed by the Principal Investigator to the trial Sponsor. The Chair of the DSMB will be contacted for advice and independent review by the Investigator or trial Sponsor in the following situations:

- The occurrence of any SAE
- Any other situation where the Investigator or trial Sponsor feels independent advice or review is important

Ethical and regulatory considerations

Further information regarding risks

The immunisations to be given have recognised side effects which are usually mild and resolve spontaneously in a few days to one week. Parenteral vaccines are likely to result in pain and swelling at the site of injection and mild fever; very occasionally pain and swelling can be severe and associated with difficulty in moving the shoulder. Sometimes headache and tiredness occur. Rarely a vaccine may cause a severe allergic reaction. For most vaccines this is estimated at less than one in a million doses (but 1 in 55,000 for Yellow Fever vaccine).[34] Individuals with a history of a possible allergic reaction to drugs or vaccines, or to vaccine components including eggs or chicken proteins, will be excluded from the studies. The research team will be trained and prepared to manage severe allergic reactions.

Adverse reactions to Yellow Fever vaccine include severe nervous system reaction (about 1 person in 125,000) and severe, life-threatening illness with organ failure (about 1 person in 250,000). The mortality for this severe, life-threatening adverse effect is reported as about 50%.[34]

BCG immunisation is likely to induce a scar in many cases. This may develop over several weeks, starting as a small papule at the injection site which may become ulcerated and then heal over a period of 2 to 5 months; and lymphadenopathy may develop. Occasionally a more severe local reaction occurs (estimated at 1 per 1,000-10,000 doses): for example, an abscess develops and scars may develop into keloids. Rarely BCG can cause disseminated disease (1 per 230,000 to 640,000 doses), or disease in sites remote from the immunisation site. Disseminated BCG disease usually occurs in immunocompromised people: HIV positive people will be excluded from these studies.[35] BCG “pre-immunisation” may interfere with the response to the subsequent live vaccines; indeed our hypothesis, and published results, suggest that it may suppress replication of YF 17D vaccine.[36] However, this reduced replication has not been shown to correlate with, or result in, reduced levels of neutralising antibody titres (which are the desired protective outcome).[13 36]

Oral typhoid vaccine (Ty21a) may occasionally be associated with stomach pain, nausea, vomiting and (rarely) rash.[34]

For peer review only

Dihydroartemisinin/piperaquine is generally well tolerated and has been used widely in practice, as well as in a substantial number of trials.[32] The main potential adverse effect of concern is cardiac toxicity, particularly dose-dependent prolongation of the QTc interval and associated increased risk of arrhythmias. There has been concern that provision of multiple doses might increase this risk. However, a recent meta-analysis involving 11 trials of DP (9 IPT, 2 treatment trials) involving 14,628 participants, 3,935 of whom received multiple treatments with DP found no evidence to suggest significant cardiac toxicity.[32] Few of the studies measured electrocardiographic changes, but those that did so found no increase in QTc intervals with increasing numbers of doses.

Benefits

All the treatments and vaccines to be given are licensed and regarded as safe. In general, the vaccines and treatments are expected to provide protection against infectious diseases.

With regard to BCG immunisation or revaccination in adolescence, benefits with respect to protection against tuberculosis among Ugandan adolescents are unknown and may, at best, be modest. There may be non-specific benefits. WHO's SAGE committee concluded, in their summary of October 2017,[37] that "BCG revaccination is safe in *Mycobacterium tuberculosis* infected and uninfected populations. There is a lack of evidence from randomized controlled trials and retrospective cohort and case-control studies demonstrating the efficacy and effectiveness of BCG revaccination in adolescents and adults after primary BCG vaccination in infancy for protection against TB disease. Due to absence of evidence, BCG revaccination is not considered cost-effective. Further research is warranted to explore whether certain sub-groups of age, geographic or M. tuberculosis exposure categories would benefit from revaccination." We hope, through this work, to contribute to this debate.

Participants and their families, schools and communities are expected to benefit from improved understanding of malaria, and of vaccines.

For peer review only

REFERENCES

1. Wajja A, Kizito D, Nassanga B, et al. The effect of current Schistosoma mansoni infection on the immunogenicity of a candidate TB vaccine, MVA85A, in BCG-vaccinated adolescents: An open-label trial. *PLoS neglected tropical diseases* 2017;11(5):e0005440. doi: 10.1371/journal.pntd.0005440 [published Online First: 2017/05/05]

2. WHO. Growth reference 5-19 years. 2007

3. WHO. Global tuberculosis report 2016 http://www.who.int/tb/publications/global_report/en/ (accessed 17 June 2017), 2016.

4. Alcais A, Fieschi C, Abel L, et al. Tuberculosis in children and adults: two distinct genetic diseases. *J Exp Med* 2005;202(12):1617-21. doi: 10.1084/jem.20052302

5. Weiner J, 3rd, Kaufmann SH. Recent advances towards tuberculosis control: vaccines and biomarkers. *J Intern Med* 2014;275(5):467-80. doi: 10.1111/joim.12212

6. Fine PE. Variation in protection by BCG: implications of and for heterologous immunity. *Lancet* 1995;346(8986):1339-45.

7. Barreto ML, Pilger D, Pereira SM, et al. Causes of variation in BCG vaccine efficacy: examining evidence from the BCG REVAC cluster randomized trial to explore the masking and the blocking hypotheses. *Vaccine* 2014;32(30):3759-64. doi: 10.1016/j.vaccine.2014.05.042

8. Black GF, Weir RE, Floyd S, et al. BCG-induced increase in interferon-gamma response to mycobacterial antigens and efficacy of BCG vaccination in Malawi and the UK: two randomised controlled studies. *Lancet* 2002;359(9315):1393-401. doi: 10.1016/S0140-6736(02)08353-8

9. Tanner R, Kakalacheva K, Miller E, et al. Serum indoleamine 2,3-dioxygenase activity is associated with reduced immunogenicity following vaccination with MVA85A. *BMC infectious diseases* 2014;14:660. doi: 10.1186/s12879-014-0660-7 [published Online First: 2014/12/04]

10. WHO. SAGE Evidence to recommendations framework. 2017. http://www.who.int/immunization/sage/meetings/2017/october/2_EvidencetoRecommendationFramework_BCG.pdf (accessed 16th March 2018).

11. Dye C. Making wider use of the world's most widely used vaccine: Bacille Calmette-Guerin revaccination reconsidered. *Journal of the Royal Society, Interface* 2013;10(87):20130365. doi: 10.1098/rsif.2013.0365 [published Online First: 2013/08/02]

12. Kraemer MU, Faria NR, Reiner RC, Jr., et al. Spread of yellow fever virus outbreak in Angola and the Democratic Republic of the Congo 2015-16: a modelling study. *The Lancet Infectious diseases* 2016 doi: 10.1016/s1473-3099(16)30513-8 [published Online First: 2016/12/27]

13. Muyanja E, Ssemaganda A, Ngauv P, et al. Immune activation alters cellular and humoral responses to yellow fever 17D vaccine. *The Journal of clinical investigation* 2014;124(7):3147-58. doi: 10.1172/jci75429 [published Online First: 2014/06/10]

14. Aguiar M, Stollenwerk N, Halstead SB. The Impact of the Newly Licensed Dengue Vaccine in Endemic Countries. *PLoS neglected tropical diseases* 2016;10(12):e0005179. doi: 10.1371/journal.pntd.0005179 [published Online First: 2016/12/22]

15. Dharmasena MN, Osorio M, Filipova S, et al. Stable expression of Shigella dysenteriae serotype 1 O-antigen genes integrated into the chromosome of live Salmonella oral vaccine vector Ty21a. *Pathogens and disease* 2016 doi: 10.1093/femspd/ftw098 [published Online First: 2016/09/23]

For peer review only

16. Carias C, Walters MS, Wefula E, et al. Economic evaluation of typhoid vaccination in a prolonged typhoid outbreak setting: the case of Kasese district in Uganda. *Vaccine* 2015;33(17):2079-85. doi: 10.1016/j.vaccine.2015.02.027 [published Online First: 2015/02/26]

17. Melhem RF, LoVerde PT. Mechanism of interaction of Salmonella and Schistosoma species. *Infection and immunity* 1984;44(2):274-81. [published Online First: 1984/05/01]

18. Muniz-Junqueira MI, Tavares-Neto J, Prata A, et al. Antibody response to Salmonella typhi in human schistosomiasis mansoni. *Revista da Sociedade Brasileira de Medicina Tropical* 1996;29(5):441-5. [published Online First: 1996/09/01]

19. WHO. Position Paper on Typhoid vaccines: WHO position paper – March 2018 2018

20. Brown J, Baisley K, Kavishe B, et al. Impact of malaria and helminth infections on immunogenicity of the human papillomavirus-16/18 AS04-adjuvanted vaccine in Tanzania. *Vaccine* 2014;32(5):611-7. doi: 10.1016/j.vaccine.2013.11.061

21. Riner DK, Ndombi EM, Carter JM, et al. Schistosoma mansoni Infection Can Jeopardize the Duration of Protective Levels of Antibody Responses to Immunizations against Hepatitis B and Tetanus Toxoid. *PLoS neglected tropical diseases* 2016;10(12):e0005180. doi: 10.1371/journal.pntd.0005180

22. Centre HI. HPV and related diseases report: Uganda. 2016. http://www.hpvcentre.net/statistics/reports/UGA.pdf (accessed 20.01.2017).

23. WHO. Human papillomavirus vaccines: WHO position paper, May 2017. *Releve epidemiologique hebdomadaire* 2017;92(19):241-68. [published Online First: 2017/05/23]

24. Nanteza B, Galukande M, Aceng J, et al. The burden of tetanus in Uganda. *SpringerPlus* 2016;5(1):705. doi: 10.1186/s40064-016-2309-z [published Online First: 2016/06/29]

25. Petralli JK, Merigan TC, Wilbur JR. ACTION OF ENDOGENOUS INTERFERON AGAINST VACCINIA INFECTION IN CHILDREN. *Lancet* 1965;2(7409):401-5. [published Online First: 1965/08/28]

26. C-A S. Vaccine Immunology. In: Plotkin SA, Orenstein W, Offit P, et al., eds. Vaccines: Elsevier 2017.

27. PHE. Public Health England: revised recommendations for the administration of more than one

live vaccine 2015

28. PHE. Immunization procedures. The Green Book. Public Health England. 2012

29. PHE. Public Health England: revised recommendations for the administration of more than one live vaccine 2015

30. Ministry of Health–Uganda. Uganda Clinical Guidelines 2016. http://apps.who.int/medicinedocs/documents/s23532en/s23532en.pdf

31. WHO. Haemoglobin concentrations for the diagnosis of anaemia and assessment of severity. Vitamin and Mineral Nutrition Information System. Geneva, World Health Organization, 2011 (WHO/NMH/NHD/MNM/11.1).

32. Gutman J, Kovacs S, Dorsey G, et al. Safety, tolerability, and efficacy of repeated doses of dihydroartemisinin-piperaquine for prevention and treatment of malaria: a systematic review and meta-analysis. *The Lancet Infectious diseases* 2017;17(2):184-93. doi: 10.1016/s1473-3099(16)30378-4 [published Online First: 2016/11/21]

For peer review only

33. Nankabirwa JI, Wandera B, Amuge P, et al. Impact of intermittent preventive treatment with dihydroartemisinin-piperaquine on malaria in Ugandan schoolchildren: a randomized, placebo-controlled trial. *Clinical infectious diseases : an official publication of the Infectious Diseases Society of America* 2014;58(10):1404-12. doi: 10.1093/cid/ciu150 [published Online First: 2014/03/14]

34. CDC. Centers for Disease Control and Prevention, vaccines and immunizations.

35. WHO. Information sheet observed rate of vaccine reactions Bacille Calmette-Guérin (BCG) vaccine. 2012

36. Arts RJW, Moorlag S, Novakovic B, et al. BCG Vaccination Protects against Experimental Viral Infection in Humans through the Induction of Cytokines Associated with Trained Immunity. *Cell host & microbe* 2018;23(1):89-100.e5. doi: 10.1016/j.chom.2017.12.010 [published Online First: 2018/01/13]

37. WHO. Immunization, vaccines and biologicals. Vaccine position papers. 2017

For peer review only

Reporting checklist for protocol of a clinical trial.

Based on the SPIRIT guidelines.

Instructions to authors

Complete this checklist by entering the page numbers from your manuscript where readers will find each of the items listed below.

Your article may not currently address all the items on the checklist. Please modify your text to include the missing information. If you are certain that an item does not apply, please write "n/a" and provide a short explanation.

Upload your completed checklist as an extra file when you submit to a journal.

In your methods section, say that you used the SPIRITreporting guidelines, and cite them as:

Chan A-W, Tetzlaff JM, Altman DG, Laupacis A, Gøtzsche PC, Krleža-Jerić K, Hróbjartsson A, Mann H, Dickersin K, Berlin J, Doré C, Parulekar W, Summerskill W, Groves T, Schulz K, Sox H, Rockhold FW, Rennie D, Moher D. SPIRIT 2013 Statement: Defining standard protocol items for clinical trials. Ann Intern Med. 2013;158(3):200-207

		Reporting Item	Page Number
**Administrative information**			
Title	#1	Descriptive title identifying the study design, population, interventions, and, if applicable, trial acronym	1
Trial registration	#2a	Trial identifier and registry name. If not yet registered, name of intended registry	2
Trial registration: data set	#2b	All items from the World Health Organization Trial Registration Data Set	n/a
Protocol version	#3	Date and version identifier	Information available at ISRCTN62041885
Funding	#4	Sources and types of financial, material, and other support	15

For peer review only

Roles and responsibilities: contributorship	#5a	Names, affiliations, and roles of protocol contributors	15
Roles and responsibilities: sponsor contact information	#5b	Name and contact information for the trial sponsor	Information available at ISRCTN62041885.
Roles and responsibilities: sponsor and funder	#5c	Role of study sponsor and funders, if any, in study design; collection, management, analysis, and interpretation of data; writing of the report; and the decision to submit the report for publication, including whether they will have ultimate authority over any of these activities	16
Roles and responsibilities: committees	#5d	Composition, roles, and responsibilities of the coordinating centre, steering committee, endpoint adjudication committee, data management team, and other individuals or groups overseeing the trial, if applicable (see Item 21a for data monitoring committee)	Information detailed in supplementary information file
**Introduction**			
Background and rationale	#6a	Description of research question and justification for undertaking the trial, including summary of relevant studies (published and unpublished) examining benefits and harms for each intervention	4
Background and rationale: choice of comparators	#6b	Explanation for choice of comparators	8
Objectives	#7	Specific objectives or hypotheses	6
Trial design	#8	Description of trial design including type of trial (eg, parallel group, crossover, factorial, single group), allocation ratio, and framework (eg, superiority, equivalence, non-inferiority, exploratory)	7
**Methods: Participants,**			

For peer review only

**interventions, and outcomes**			
Study setting	#9	Description of study settings (eg, community clinic, academic hospital) and list of countries where data will be collected. Reference to where list of study sites can be obtained	7
Eligibility criteria	#10	Inclusion and exclusion criteria for participants. If applicable, eligibility criteria for study centres and individuals who will perform the interventions (eg, surgeons, psychotherapists)	7
Interventions: description	#11a	Interventions for each group with sufficient detail to allow replication, including how and when they will be administered	8
Interventions: modifications	#11b	Criteria for discontinuing or modifying allocated interventions for a given trial participant (eg, drug dose change in response to harms, participant request, or improving / worsening disease)	Detailed in supplementary information file
Interventions: adherance	#11c	Strategies to improve adherence to intervention protocols, and any procedures for monitoring adherence (eg, drug tablet return; laboratory tests)	Detailed in supplementary information file
Interventions: concomitant care	#11d	Relevant concomitant care and interventions that are permitted or prohibited during the trial	Detailed in supplementary information file
Outcomes	#12	Primary, secondary, and other outcomes, including the specific measurement variable (eg, systolic blood pressure), analysis metric (eg, change from baseline, final value, time to event), method of aggregation (eg, median, proportion), and time point for each outcome. Explanation of the clinical relevance of chosen efficacy and harm outcomes is strongly recommended	10
Participant timeline	#13	Time schedule of enrolment, interventions (including any run-ins and washouts), assessments, and visits for participants. A schematic diagram is highly recommended (see Figure)	Detailed in supplementary information file, Table S1

For peer review only

Sample size	#14	Estimated number of participants needed to achieve study objectives and how it was determined, including clinical and statistical assumptions supporting any sample size calculations	11
Recruitment	#15	Strategies for achieving adequate participant enrolment to reach target sample size	12
**Methods: Assignment of interventions (for controlled trials)**			
Allocation: sequence generation	#16a	Method of generating the allocation sequence (eg, computer-generated random numbers), and list of any factors for stratification. To reduce predictability of a random sequence, details of any planned restriction (eg, blocking) should be provided in a separate document that is unavailable to those who enrol participants or assign interventions	8
Allocation concealment mechanism	#16b	Mechanism of implementing the allocation sequence (eg, central telephone; sequentially numbered, opaque, sealed envelopes), describing any steps to conceal the sequence until interventions are assigned	8
Allocation: implementation	#16c	Who will generate the allocation sequence, who will enrol participants, and who will assign participants to interventions	8
Blinding (masking)	#17a	Who will be blinded after assignment to interventions (eg, trial participants, care providers, outcome assessors, data analysts), and how	9
Blinding (masking): emergency unblinding	#17b	If blinded, circumstances under which unblinding is permissible, and procedure for revealing a participant's allocated intervention during the trial	Detailed in supplementary information file
**Methods: Data collection, management, and analysis**			

For peer review only

Data collection plan	#18a	Plans for assessment and collection of outcome, baseline, and other trial data, including any related processes to promote data quality (eg, duplicate measurements, training of assessors) and a description of study instruments (eg, questionnaires, laboratory tests) along with their reliability and validity, if known. Reference to where data collection forms can be found, if not in the protocol	12
Data collection plan: retention	#18b	Plans to promote participant retention and complete follow-up, including list of any outcome data to be collected for participants who discontinue or deviate from intervention protocols	Detailed in supplementary information file
Data management	#19	Plans for data entry, coding, security, and storage, including any related processes to promote data quality (eg, double data entry; range checks for data values). Reference to where details of data management procedures can be found, if not in the protocol	13
Statistics: outcomes	#20a	Statistical methods for analysing primary and secondary outcomes. Reference to where other details of the statistical analysis plan can be found, if not in the protocol	11 and in the statistical analysis plan at ISRCTN62041885
Statistics: additional analyses	#20b	Methods for any additional analyses (eg, subgroup and adjusted analyses)	
Statistics: analysis population and missing data	#20c	Definition of analysis population relating to protocol non-adherence (eg, as randomised analysis), and any statistical methods to handle missing data (eg, multiple imputation)	Statistical analysis plan at ISRCTN62041885
**Methods: Monitoring**			
Data monitoring: formal committee	#21a	Composition of data monitoring committee (DMC); summary of its role and reporting structure; statement of whether it is independent from the sponsor and competing interests; and reference to where further details about its charter can be found, if not in the	Detailed in supplementary information file

For peer review only

		protocol. Alternatively, an explanation of why a DMC is not needed	
Data monitoring: interim analysis	#21b	Description of any interim analyses and stopping guidelines, including who will have access to these interim results and make the final decision to terminate the trial	Detailed in supplementary information file
Harms	#22	Plans for collecting, assessing, reporting, and managing solicited and spontaneously reported adverse events and other unintended effects of trial interventions or trial conduct	Detailed in supplementary information file
Auditing	#23	Frequency and procedures for auditing trial conduct, if any, and whether the process will be independent from investigators and the sponsor	Detailed in supplementary information file
**Ethics and dissemination**			
Research ethics approval	#24	Plans for seeking research ethics committee / institutional review board (REC / IRB) approval	11
Protocol amendments	#25	Plans for communicating important protocol modifications (eg, changes to eligibility criteria, outcomes, analyses) to relevant parties (eg, investigators, REC / IRBs, trial participants, trial registries, journals, regulators)	11
Consent or assent	#26a	Who will obtain informed consent or assent from potential trial participants or authorised surrogates, and how (see Item 32)	Detailed in supplementary information file
Consent or assent: ancillary studies	#26b	Additional consent provisions for collection and use of participant data and biological specimens in ancillary studies, if applicable	Detailed in supplementary information file
Confidentiality	#27	How personal information about potential and enrolled participants will be collected, shared, and maintained in order to protect confidentiality before, during, and after the trial	Detailed in supplementary information file
Declaration of interests	#28	Financial and other competing interests for principal investigators for the overall trial and each study site	15

For peer review only

Data access	#29	Statement of who will have access to the final trial dataset, and disclosure of contractual agreements that limit such access for investigators	n/a
Ancillary and post trial care	#30	Provisions, if any, for ancillary and post-trial care, and for compensation to those who suffer harm from trial participation	Detailed in supplementary information file
Dissemination policy: trial results	#31a	Plans for investigators and sponsor to communicate trial results to participants, healthcare professionals, the public, and other relevant groups (eg, via publication, reporting in results databases, or other data sharing arrangements), including any publication restrictions	11-12
Dissemination policy: authorship	#31b	Authorship eligibility guidelines and any intended use of professional writers	n/a
Dissemination policy: reproducible research	#31c	Plans, if any, for granting public access to the full protocol, participant-level dataset, and statistical code	12
**Appendices**			
Informed consent materials	#32	Model consent form and other related documentation given to participants and authorised surrogates	Supplementary files provided
Biological specimens	#33	Plans for collection, laboratory evaluation, and storage of biological specimens for genetic or molecular analysis in the current trial and for future use in ancillary studies, if applicable	n/a

None The SPIRIT checklist is distributed under the terms of the Creative Commons Attribution License CC-BY-ND 3.0. This checklist can be completed online using https://www.goodreports.org/, a tool made by the EQUATOR Network in collaboration with Penelope.ai

For peer review only
